# Effectiveness and Mechanisms of a Digital Mindfulness–Based Intervention for Subthreshold to Clinical Insomnia Symptoms in Pregnant Women: Randomized Controlled Trial

**DOI:** 10.2196/68084

**Published:** 2025-05-05

**Authors:** Juan Wang, Qiuhong Yang, Naixue Cui, Liuliu Wu, Xuan Zhang, Yaoyao Sun, Yongqi Huang, Fenglin Cao

**Affiliations:** 1 School of Nursing and Rehabilitation Cheeloo College of Medicine Shandong University Jinan China; 2 Affiliated Mental Health Center & Hangzhou Seventh People's Hospital and School of Brain Science and Brain Medicine Zhejiang University School of Medicine Hangzhou China; 3 Department of Obstetrics Jinan Maternity and Child Care Hospital Affiliated to Shandong First Medical University Jinan China; 4 Research Center of Clinical Epidemiology Peking University Third Hospital Beijing China; 5 Peking University Health Science Center Beijing China; 6 Peking University Sixth Hospital, Peking University Institute of Mental Health, NHC Key Laboratory of Mental Health (Peking University), National Clinical Research Center for Mental Disorders (Peking University Sixth Hospital) Beijing China

**Keywords:** digital interventions, mindfulness-based interventions, pregnant women, prenatal insomnia, sleep during pregnancy, randomized controlled trial, mechanisms of change

## Abstract

**Background:**

Prenatal insomnia symptoms are prevalent, debilitating, and largely untreated; yet, there is a lack of empirically supported and accessible interventions. Mindfulness-based interventions have been theoretically hypothesized to alleviate insomnia symptoms by counteracting adverse sleep-related cognitive and behavioral processes, although few studies have tested them.

**Objective:**

This study aimed to examine the effectiveness and potential mechanisms of a digital mindfulness-based intervention targeted at prenatal insomnia (dMBI-PI) in reducing insomnia symptoms.

**Methods:**

A single-blind randomized controlled trial was conducted from October 2021 to February 2023. A total of 160 eligible pregnant women (mean age 30.54, SD 3.86 years) with subthreshold to clinical insomnia symptoms (corresponding to a score of ≥8 on the Insomnia Severity Index) were recruited from obstetrics clinics and then randomized 1:1 into the intervention group (the 6-week dMBI-PI plus standardized care) or the control group (standardized care). The primary outcome was the insomnia symptoms assessed at baseline, immediately after the intervention, 2 months after the intervention (approximately the third trimester), and 42 days post partum. The secondary outcomes included insomnia remission rates and reliable change rates, sleep onset latency, wake after sleep onset, total sleep time, sleep efficiency, sleep quality, fatigue symptoms, daytime sleepiness, anxiety, and depressive symptoms. The hypothesized mediators included sleep-specific rumination, sleep-specific worry, presleep arousal, sleep-related attentional bias, and maladaptive behaviors. All outcomes were self-assessed through web-based questionnaires. Linear mixed model analysis was conducted to examine the dMBI-PI’s effects.

**Results:**

Compared with the control group, the intervention group had significantly greater improvements in insomnia symptoms from baseline to the end of the intervention (mean between-group difference –2.02, 95% CI –3.42 to –0.62; *P*=.005; Cohen *d*=0.46, 95% CI 0.01-0.92) and from baseline to the third trimester (mean between-group difference –2.02, 95% CI –3.42 to –0.61; *P*=.005; Cohen *d*=0.46, 95% CI 0.01-0.92), but a beneficial effect was not observed post partum. The intervention group had a significantly increased likelihood of achieving insomnia remission or reliable change at the third trimester; however, we did not observe significant between-group differences in the changes in most secondary outcomes. The changes in adverse cognitive and behavioral processes (mainly sleep-specific worry and presleep arousal) significantly mediated the dMBI-PI’s effect on prenatal insomnia symptoms.

**Conclusions:**

The dMBIs showed significant short-term benefits for prenatal insomnia symptoms by mitigating sleep-specific worry and presleep arousal and may therefore hold promise as a first-step, pragmatic, and accessible option for pregnant women at risk of insomnia.

**Trial Registration:**

Chinese Clinical Trial Register (ChiCTR) ChiCTR2100052269; https://tinyurl.com/4bb8f7ah

## Introduction

### Background

Insomnia symptoms are common during pregnancy [1] and may adversely affect maternal and child well-being, including increasing the risk of perinatal depression and various adverse pregnancy and perinatal outcomes (eg, pregnancy loss, preeclampsia, gestational hypertension, gestational diabetes mellitus, stillbirth, preterm birth, and large for gestational age) [2,3], as well as leading to poorer neurocognitive function and social-emotional development in offspring [4,5]. However, empirically supported interventions to improve prenatal insomnia symptoms are limited. Several studies have documented the effectiveness of cognitive behavioral therapy for insomnia (CBT-I, the first-line insomnia treatment recommended by many guidelines; with most of the evidence coming from the general population) among pregnant women, but only 34.8% to 63.8% achieved remission from prenatal insomnia symptoms [6-8]. Moreover, the accessibility of CBT-I is significantly hindered by the availability of trained clinicians and high treatment costs [6]. While all patients face challenges in accessing this intervention, individuals with subthreshold insomnia symptoms may be particularly disadvantaged because they are often misperceived as not requiring urgent intervention or assumed to have symptoms that would resolve without formal treatment [9]. Considering that prenatal insomnia symptoms appear to be influenced by several unique factors (eg, hormonal changes, increased uterus size, and frequent urination) in comparison to the general population [10], identifying other effective treatments for prenatal insomnia symptoms is critical to prevent adverse maternal and fetal consequences.

Accumulating evidence has suggested that mindfulness-based interventions (MBIs) may hold promise as an additional treatment option for insomnia symptoms. According to the theoretical model for the effects of mindfulness practices on risk processes for the development and maintenance of sleep disturbances proposed by Shallcross et al [11], the core principles of MBIs, such as present-moment awareness, nonjudgment, and acceptance, may theoretically improve sleep by counteracting the adverse cognitive and behavioral processes implicated in insomnia. These processes have been widely suggested across various etiologic models of insomnia, including sleep-specific rumination (repetitive negative thinking about the causes of current daytime symptoms associated with poor sleep, such as fatigue, poor concentration, and disturbed mood); sleep-specific worry (repetitive negative thinking toward future adverse consequences of poor sleep); presleep arousal (increased presleep cognitive activity or physical arousal similar to that of the acute stress response driven by the activation of the sympathetic nervous system, eg, increased heart rate, elevated body temperature, and muscle tension); sleep-related attentional bias (increased scanning or monitoring toward anything that could be perceived as sleep-related threats, including both internal stimuli, such as bodily sensations, and external stimuli, such as noise, light, or clocks); and maladaptive behaviors (subtle behaviors that individuals engage in to cope with the fear of not being able to sleep and its negative consequences, eg, consuming caffeine to prevent daytime dysfunction, going to bed early to ensure enough time to fall asleep, or looking at the clock to see how long it takes to fall asleep) [12].

Ong et al [13] developed a mindfulness-based therapy for insomnia (MBT-I) in 2008, combining mindfulness meditation practices with the behavior therapy of CBT-I (such as sleep hygiene, stimulus control, and sleep restriction). Because automatic thoughts might be very resistant to change, substituting negative thoughts and beliefs with rational ones has been widely recognized as one of the most challenging steps in CBT-I. In comparison, a mindfulness-based approach emphasizes shifting from automatic reactions to a more detached, process-oriented observation and acceptance toward thoughts and decreasing the struggle with them rather than seeking to change them, which may offer a more acceptable solution for patients [14]. Meanwhile, this approach provides a conceptual framework for effectively managing undesirable mental and physical states associated with insomnia, which could complement and facilitate the implementation of behavioral strategies for insomnia [13]. For example, stimulus control suggests getting out of bed if one realizes sleep may not occur soon. This instruction implicitly conveys the principles of accepting that sleep may not come immediately and giving up the efforts to stay in bed for a period when awake to fall asleep, which are theoretically congruent with the goal of MBIs to help individuals increase awareness of internal states associated with insomnia and to develop more adaptive ways (eg, nonstriving, acceptance, and promoting behavioral changes) to respond to these undesirable states rather than reacting automatically and habitually (eg, trying to stop thinking entirely) [15]. In addition, sleep restriction suggests strictly limiting the amount of time in bed and avoiding daytime naps, which may be challenging in terms of adherence as participants may experience more severe discomforts in a short period (eg, daytime fatigue and related emotional reactions). The mindful approach can foster a nonreactive and accepting attitude toward these unpleasant experiences, thereby motivating individuals to adhere to behaviors that might improve sleep [15]. A few preliminary studies to date on the general population have supported the effectiveness of MBIs (including traditional mindfulness-based stress reduction, mindfulness-based cognitive therapy, and multicomponent interventions that integrated other components within the context of mindfulness principles like MBT-I) on improving insomnia symptoms [16-18]; however, only a few pilot studies and small sample randomized controlled trials (RCTs) have explored the effectiveness of MBIs on prenatal insomnia symptoms [19-21]. More adequately powered RCT studies are still warranted to provide confirmative evidence.

In addition, digital adaptations of psychological interventions have been widely advocated over the past decade to overcome the dissemination barriers prevalent in in-person treatments (eg, a shortage of professional practitioners, treatment costs, and time or location restrictions) [22]. Preliminary research has demonstrated the effectiveness of digital MBIs in improving sleep among nonpregnant populations [23-25]. Regrettably, although there are several theoretical and practical considerations suggesting that such flexible digital delivery may be especially attractive to pregnant women (eg, they are predominantly young adults, they have relatively superior eHealth literacy and technology acceptance [26], they often have numerous medical appointments making it challenging to attend additional face-to-face sessions specifically for insomnia symptoms, and for whom timeliness of access to interventions is critical), few studies have established whether the digital delivery of MBIs can improve prenatal insomnia symptoms. One published protocol for a pilot RCT (with an expected sample size of 50) is planned to assess the effects of a web-based mindfulness meditation intervention on the treatment of insomnia and prevention of depression relapse during pregnancy [27]; however, its results have not yet been publicly reported.

In addition to revealing the intervention effects, investigating the mediators by which MBIs achieve their effects is also important for identifying the most effective components and potentially informing how to refine intervention protocols. However, most studies to date assessing the effectiveness of MBIs fail to include a previous hypothesis about mediators in the modeling of program effects. Moreover, several studies tend to stop the mediation testing and conclude that the mediation is not present because of a nonsignificant total effect [28]. Recent methodological research using empirical simulation has demonstrated that researchers may detect statistically significant mediating effects even when the total effect is not (one possible condition is that the mediating effect is equal to the total effect, and the statistical power to detect the mediating effect is much larger than that of the total effect) [29]; that is, regardless of whether the intervention effect is present, testing mediation in intervention research is valuable to provide researchers additional information to understand how an intervention achieved or failed to achieve its effects as well as which components were successful or unsuccessful [28]. According to the aforementioned theoretical model for the effects of mindfulness practices on risk processes for the development and maintenance of sleep disturbances by Shallcross et al [11], adverse cognitive and behavioral factors may be among the most promising candidates for elucidating the mechanisms by which MBIs improve insomnia. However, to the best of our knowledge, only one well-designed RCT has investigated the mediating roles of rumination and worry in the MBIs’ effects on insomnia symptoms among patients with breast cancer, which found that both rumination and worry were significant mediators underlying the effects of MBIs on insomnia symptoms [30].

### Objectives

The first objective of this study was to evaluate the effectiveness of a digital mindfulness-based intervention targeted at prenatal insomnia (dMBI-PI) in reducing subthreshold to clinical insomnia symptoms through an RCT with a relatively large sample size and long-term follow-up from the second trimester to post partum. Furthermore, to better understand the mechanisms of change, our second objective was to investigate whether the intervention could improve the theoretically hypothesized mediators (including sleep-specific rumination, sleep-specific worry, presleep arousal, sleep-related attentional bias, and maladaptive behaviors) and whether their changes mediated the dMBI-PI’s effect on prenatal insomnia symptoms. We hypothesized that pregnant women randomized to receive dMBI-PI would report significantly greater improvements in insomnia symptoms and adverse cognitive and behavioral processes than those receiving standardized care, and the improvements in adverse cognitive and behavioral processes would play mediating roles in the dMBI-PI’s effect on prenatal insomnia symptoms in both the separate mediation models and a parallel multiple mediation model.

## Methods

### Study Design and Participants

This study was an evaluator-masked, 2-parallel-armed RCT conducted in 2 tertiary hospitals in Shandong, China, from October 2021 to July 2022 (follow-up was completed in February 2023). Eligible participants had to meet the preestablished inclusion and exclusion criteria, as provided in Textbox 1.

**Inclusion and exclusion criteria.**

**Inclusion criteria**

- Aged at least 18 years
- Singleton pregnancy
- Between 12 and 20 weeks of gestation (according to the clinical practices of obstetrics in China, most pregnant women start to receive regular checkups at the hospital after establishing the Maternal and Children’s Health Handbook in the community around the 12th gestational week)
- At least a junior high school education
- Planning to receive regular checkups and deliver at the research hospitals
- A score of ≥8 on the Insomnia Severity Index (corresponding to subthreshold to clinical insomnia symptoms)
- Having a WeChat (Tencent Holdings Limited) account and, after receiving an in-person demonstration or remote guidance via WeChat messages, self-reporting the ability to use the WeChat miniprogram
- Clear consciousness, normal understanding and language expression ability, and ability to fill questionnaires independently
- No use of nondrug treatments for insomnia (eg, massage) in the last 6 months and not on the waiting lists for other interventions

**Exclusion criteria**

- Self-reported severe physical illness (eg, autoimmune diseases; severe heart, liver, or kidney failure; malignant tumors; hematologic disorders; or uncontrolled seizure disorder) or mental illness (eg, lifetime bipolar disorder or schizophrenia, current major depressive disorder, posttraumatic stress disorder, or substance abuse disorders)
- Current use of prescription or nonprescription medications for sleep (eg, benzodiazepines, melatonin, or herbal supplements)
- Self-reported prepregnancy or current diagnosis of sleep disorders (eg, obstructive sleep apnea, periodic limb movement disorder, restless legs syndrome, or nightmare disorder)
- Current shift work or night work
- Suspected severe depressive symptoms (a score of ≥19 on the Edinburgh Postnatal Depression Scale [EPDS])
- Active suicidality (a score of ≥2 on the 10th item of EPDS, indicating that the thoughts of harming themselves occur sometimes or quite often)
- Hospitalized for fetus protection treatments due to severe pregnancy complications
- Previously attended formal mindfulness meditation courses or had regular meditation practice experience (eg, at least 3 times per week)
- Refusal to participate

### Procedures

Random number tables were generated and stored in numbered, sealed, and opaque envelopes by a researcher independent of the research team. When eligible participants completed baseline assessments and provided informed consent, the independent researcher opened the envelope and randomly assigned participants to the dMBI-PI intervention group or the control group using a 1:1 ratio. Because the control group did not receive the same digital intervention courses, it was difficult to keep the treatment implementers and participants blinded to treatment assignments. However, one of the advantages of digital interventions is that they provide a low-touch, structured format that supports the implementation of interventions with high fidelity, that is, ensuring the intervention content is delivered as intended or designed; meanwhile, the outcome assessments during the study period were administered using self-assessed questionnaires, and the assignment was concealed from the data collectors responsible for distributing the questionnaires, which may potentially alleviate the risk of contamination from nonblind treatment implementers. The baseline (time point 1) and follow-up (including the end of the intervention [time point 2], 2 months after the intervention [time point 3], and 42 days post partum [time point 4]) assessments were conducted through a web-based survey platform [31]. After sending a link of the assessment questionnaire, the research staff sent WeChat reminders every 2 days. Participants who refused to participate in the follow-up assessments, deleted the WeChat contact, or failed to complete the questionnaire after 3 WeChat reminders at 2 consecutive time points were considered to have withdrawn from the follow-up.

### Ethical Considerations

This study received approval from the Ethics Committee of the Shandong University School of Nursing and Rehabilitation (2020-R-063). All participants provided electronic informed consent. The trial was registered with the Chinese Clinical Trial Registry (ChiCTR2100052269), and the detailed trial protocol is available in Multimedia Appendix 1 [1-4,6-8,11,16-21,23-25,27,30,32-66]. All data from participants were strictly confidential and protected by privacy law. Personal information was anonymized during the data use and transmission. Participants were not paid for their participation in the study. However, to enhance study participation and retention, all participants had access to free antenatal health consultations provided by experienced obstetricians or trained clinic assistants who were unaware of the study allocation. The consultation content typically included antenatal care tips, interpretations of results from physical examinations, the next checkup schedules and details, and advice on drug use, among others. This study followed the standard guidelines for reporting parallel group RCTs.

### Interventions

**Figure 1:**
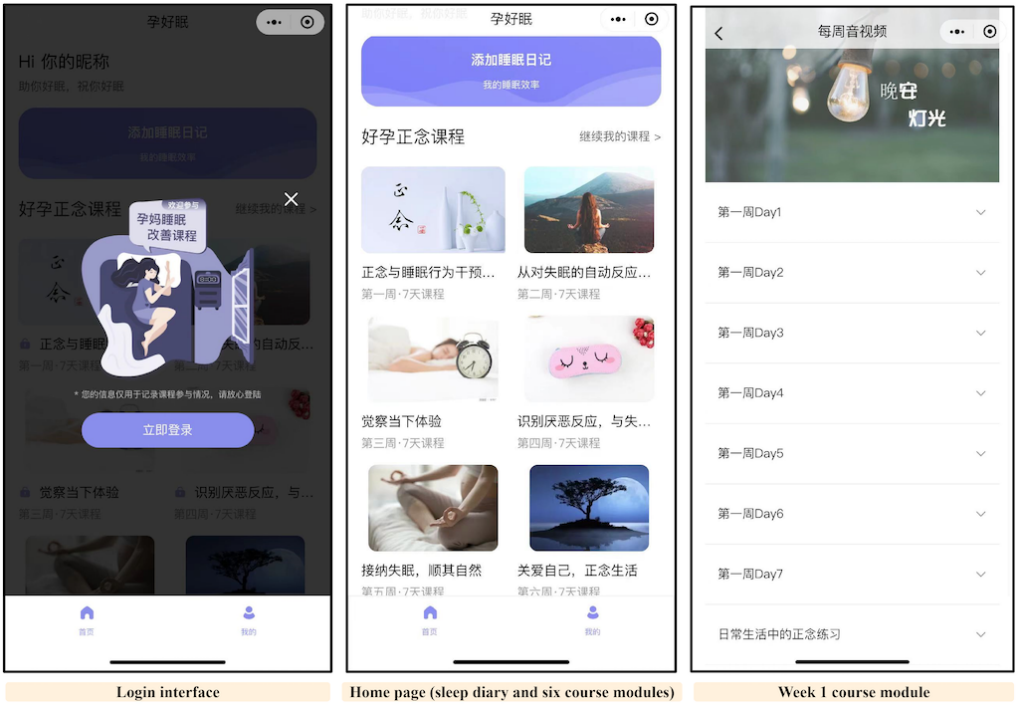
Screenshots of the WeChat miniprogram called “Mom Sleep Well” that was used to deliver the digital mindfulness-based intervention for prenatal insomnia symptoms.

The 6-week dMBI-PI was adapted from the MBT-I manual by Ong [32] and the mindfulness-based cognitive therapy manual by Teasdale et al [33]. An experienced professor, with a doctoral degree in psychological medicine, led the adaptation of the treatment content tailored to pregnant women [34,67,68]. For example, in the thematic courses, examples were adapted to suit pregnant women’s characteristics (eg, when addressing negative thoughts related to insomnia, an example like “If I keep experiencing insomnia symptoms, it will affect my baby’s development. If my baby isn’t healthy, our family will be ruined...” was provided); in the mindfulness practices, we included guidance on mindfulness awareness of fetal movements; for sleep restriction, following previous studies on pregnant women [6], the recommended lowest time in bed was set at 5.5 hours, unlike the 5-hour minimum suggested for the general population in the internationally standardized treatment protocol [69]. The dMBI-PI contained 6 weekly course modules. Each course module consisted of a video-based thematic course and 6 days of audio-based formal mindfulness practices; participants were also encouraged to adhere to informal mindfulness practices (eg, mindful eating, walking, or 3-minute breathing practices) and sleep-promoting behaviors daily (details are provided in Table 1). Participants in the dMBI-PI intervention group could access 6 course modules via a WeChat miniprogram called “Mom Sleep Well” (screenshots of the miniprogram are shown in Figure 1). These modules could be unlocked successively after inputting the invitation code, and for each module, the thematic course could be unlocked first, followed by the 6-day home practices in turn. When each module was unlocked, research staff sent course reminders to participants using standardized WeChat messages.

The control group received standardized care (treatment as usual) from obstetric staff, including regular telephone follow-ups, health education, pregnancy risk assessments and management, and nutrition and lifestyle guidance. There were no limits on the use of nonstudy treatments. To balance potential biases due to researcher contact, participants in the control group were also contacted weekly using standardized WeChat messages.

**Table 1:** The treatment content of the digital mindfulness-based intervention for prenatal insomnia symptoms.

Module and theme	Video-based thematic courses	Home practices		
		Formal practices (6 days per week)	Recommended daily practice	
			Informal practices	Sleep-promoting behaviors
1. Mindfulness and sleep-related behavioral strategies to sleep well during pregnancy	The manifestation and prevalence of prenatal insomnia symptoms and the significance of early intervention Factors associated with the development and persistence of prenatal insomnia symptoms Introduction to the intervention courses The purpose and importance of keeping a sleep diary during the intervention	Body scan	Be mindful in daily life (mindful eating)	—^a^
2. Disengaging from the automatic response to insomnia	Giving examples of our habitual, automatic responses to insomnia Practicing a body scan and getting out of the automatic response The basic principles, significance, and implementation guidance of sleep restriction and stimulus control	Body scan	Be mindful in daily life (mindful eating, brushing, walking, or bathing)	Sleep restriction and stimulus control
3. Being aware of the present experience	Practicing mindful breathing and focusing on the present moment Practicing awareness of attention drift and gently bringing it back Providing guidance on how to adjust time in bed based on sleep diary data	Mindful breathing	Be mindful in daily life (mindful eating, brushing, walking, or bathing)	Sleep restriction and stimulus control
4. Identifying adverse reactions and coexisting with insomnia experience for a short time	Understanding our adverse responses to unpleasant experiences like insomnia Learning to use 3-min breathing practices as the first step in responding to an unpleasant experience Briefly reviewing the methods to further adjust time in bed based on sleep diary data and providing recommendations on sleep hygiene	Mindful sitting meditation	3-min breathing space	Sleep restriction, stimulus control, and sleep hygiene
5. Accepting insomnia and going with the flow	The importance of allowing sleep to be spontaneous Practicing allowing and letting sleep go with the flow Recognizing that insomnia-related thoughts are not facts and learning how to recognize that “thoughts are just thoughts”	Mindfulness meditation for dealing with difficulties (such as insomnia)	3-min breathing space	Sleep restriction, stimulus control, and sleep hygiene
6. Caring for yourself and living mindfully	Reviewing the core content of the courses Why and how to keep practicing mindfulness How to care for ourselves better in daily life and prevent and mitigate the prevalence and persistence of insomnia symptoms	Choose one mindfulness practice from the previous 5 wk	Be mindful in daily life or practice 3-min breathing space	Sleep restriction, stimulus control, and sleep hygiene

^a^Not available.

### Measures

#### Primary Outcome

Insomnia symptoms were assessed using the Insomnia Severity Index (ISI) from time point 1 to time point 4. In prior research [6,8,35], the ISI has been validated as a reliable measure for perinatal insomnia symptoms, and a cutoff score of 8 and 11 has been widely adopted to identify participants experiencing subthreshold insomnia symptoms and clinical insomnia symptoms, respectively. Correspondingly, a total score of <8 has been commonly used as the criterion for remission from insomnia symptoms. In addition, based on the formula for calculating the reliable change (the SE of the measurement × 2.77, while the SE of the measurement=SD×√(1–*r*)), we substituted the ISI pretest SD score and a test-retest reliability of 0.887 [70] into the formula, and finally, a reliable change criterion on the ISI of 2.52 points was calculated [71]. In this trial, the continuous score of ISI was treated as the primary outcome, while the insomnia remission rates and reliable change rates were treated as the secondary outcomes.

#### Secondary Outcomes

In addition to the insomnia remission rates and reliable change rates, 6 continuous secondary outcome measures were chosen following the recommendations for a standard research assessment of insomnia [72]: the Consensus Sleep Diary-Core for subjective sleep patterns (including sleep onset latency [SOL], the time taken to fall asleep after going to bed; wake after sleep onset [WASO], the total time spent awake after initially falling asleep; total sleep time [TST], and sleep efficiency [the percentage of time spent asleep relative to the time spent in bed], which were calculated by averaging data across 1 week; participants who wrote diaries for at least 3 days were eligible; longer SOL or WASO, shorter TST, or lower sleep efficiency were indicators of poorer sleep) [36]; the Pittsburgh Sleep Quality Index (PSQI) for sleep quality [37]; the Flinders Fatigue Scale (FFS) for fatigue symptoms [38]; the Epworth Sleepiness Scale (ESS) for daytime sleepiness [39]; the Generalized Anxiety Disorder-7 (GAD-7) for anxiety symptoms [40]; and the Edinburgh Postnatal Depression Scale (EPDS) for depressive symptoms [41]. Higher scores on the PSQI, FFS, ESS, GAD-7, and EPDS indicate poorer sleep quality, more severe fatigue symptoms, daytime sleepiness, and anxiety and depression symptoms, respectively. These measures have been widely used in perinatal women and demonstrated satisfactory psychometric properties in previous research [73-75]. With the exception of sleep diaries, which were only used at time point 1 and time point 2, other measures were conducted from time point 1 to time point 4.

#### Adherence, Use of Nonstudy Treatments, and Adverse Events

Adherence was assessed by the completion of thematic courses or home practices recorded in the backend management system of the WeChat miniprogram. Referring to previous research among perinatal women [34,42], a module was defined as completed when participants completed the thematic course or at least 3 days of mindfulness practices, and participants who completed at least half (3/6, 50%) of the modules were considered adherent. At the end of the intervention, participants were asked to self-report their use of nonstudy insomnia-related treatments, such as sleep medication or nondrug treatments. Adverse events were gathered through emergency contacts and medical records.

#### Hypothesized Mediators

Guided by the theoretical model for the effects of mindfulness practices on risk processes for the development and maintenance of sleep disturbances [11], we chose the following measures for hypothesized mediators: the Daytime Insomnia Symptom Response Scale (DISRS) for sleep-specific rumination (eg, think, “How do I not have the energy to get through the day?”) [43]; the Anxiety and Preoccupation about Sleep Questionnaire (APSQ) for sleep-specific worry (eg, “I worry about how the amount of sleep I get is going to affect my health”) [44]; the Pre-Sleep Arousal Scale (PSAS) for presleep arousal (eg, “thoughts keep running through your head”; “heart racing, pounding or beating irregularly”) [45]; the brief version of the Sleep-Associated Monitoring Index (SAMI-B) for sleep-related attentional bias (eg, “noticing how long it is taking to fall asleep when falling asleep or getting back to sleep”; “noticing noises in the house when falling asleep or getting back to sleep”) [46]; and the Sleep-Related Behaviors Questionnaire (SRBQ) for sleep-related maladaptive behaviors (eg, “I give myself lots of time to fall asleep by going to bed early”; “I limit myself to mundane chores or tasks during the day/evening”) [47]. In existing studies, the aforementioned continuous measures showed acceptable reliability and validity in perinatal women [44,76,77], with higher scores indicating greater endorsement of these negative cognitive and behavioral processes.

### Statistical Analysis

According to the medium effect of digital MBIs on prenatal depression symptoms observed in our previous study [34] and a similar or larger effect on insomnia symptoms observed in a previous RCT with a small sample size [48], 160 participants were calculated to detect a moderate between-group difference via a 2-sample *t* test of the mean change scores in ISI from baseline to the end of the intervention, based on a statistical power of 80%, a 2-tailed 5% significance level, and a 20% attrition rate. Considering that the mixed-effects approach used for the analysis is more efficient than a 2-sample *t* test, our actual statistical power was expected to be higher than 80%.

All analyses were performed using SPSS (version 25.0; IBM Corp) and R (version 4.2.2; R Foundation for Statistical Computing). Following the intention-to-treat principle, linear mixed model analysis where we included group, time, and group-by-time interactions as the explanatory variables and random intercepts to account for the within-participant correlation of repeated responses was conducted for ISI (primary outcome), a series of continuous secondary outcomes, and hypothesized mediators separately. This method allowed us to include available data from pregnant women who missed the follow-up assessments at any time point, and the group-by-time interactions were used to measure the differences in within-participant outcome changes between the dMBI-PI intervention and control group [49]. Effect sizes (Cohen *d*) were calculated by dividing the between-group differences at the end of the intervention or follow-up by the pooled SDs of the continuous outcomes at baseline, with 0.2, 0.5, and 0.8 corresponding to small, moderate, or large effect sizes, respectively [8]. For the secondary outcomes of remission and reliable change, logistic regression models (with dropouts defined as no remission and no reliable change, respectively) were fitted to assess whether the proportions of participants with remitted insomnia symptoms and achieving reliable change differed between the two groups during follow-up. We corrected for the increased probability of type I error due to multiple testing of multiple secondary outcomes or hypothesized mediators using the Benjamini-Hochberg false discovery rate (FDR) correction. To assess the robustness of the results, sensitivity analyses using datasets after performing multiple imputation, using participants who completed all follow-up assessments (complete cases analysis), or using participants who actually received the treatment (as-treated analysis) were conducted. Moreover, we examined whether there was a difference in the treatment effect (indicated by their remission status at follow-up or reliable change status from baseline to follow-up) for adherent versus nonadherent participants in the intervention group through Chi-squared tests. More details are available in Multimedia Appendix 2 [8,49,78].

To examine the extent to which the dMBI-PI’s effect on perinatal insomnia symptoms was mediated by changing hypothesized mediators (the hypothetical models are shown in Multimedia Appendix 3), potential eligible mediators showing statistically significant differences in with-participant change between the dMBI-PI group and control group after the FDR correction were further included in the single mediation models and a parallel multiple mediation model that were fitted using the PROCESS 3.3. To account for the confounding effects of baseline scores and possible random errors of measurement, the residualized change scores of ISI and hypothesized mediators (from baseline to the end of the intervention) obtained from the linear regression models were used to fit mediation models. Sensitivity analyses using the raw change scores (measured by subtracting the postintervention scores from baseline) were performed to validate the robustness of results. To facilitate attempts to establish causal and temporal correlations, we also repeated the mediation analysis to examine whether the dMBI-PI’s effect on insomnia symptoms at time point 3 could be mediated by hypothesized mediators at time point 2. The bias-corrected bootstrapping (n=5000) was used to test the 95% CI of the indirect effect.

## Results

### Participant Enrollment and Baseline Characteristics

**Figure 2:**
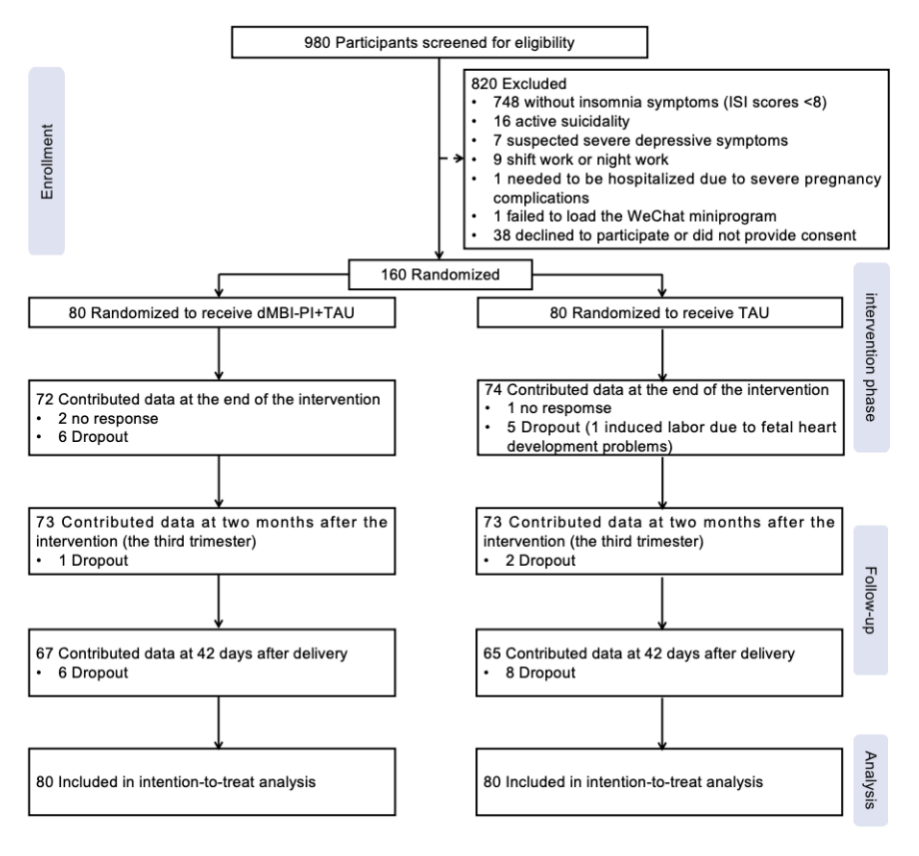
CONSORT (Consolidated Standards for Reporting Trials) 2010 participant flow diagram. dMBI-PI: digital mindfulness-based intervention targeted at prenatal insomnia; ISI: Insomnia Severity Index; TAU: treatment as usual. No response: participants missed or failed to complete an assessment for various reasons, but still remained in the study and participated in subsequent follow-ups. Dropout: participants withdrew from the study and did not take part in any future follow-ups.

A total of 980 pregnant women joined the screening process, of which 160 (16.3%) eligible pregnant women with a mean age of 30.54 (SD 3.86) years were included in this trial. Most participants were excluded as they failed to meet the insomnia criteria (see Figure 2 for further details). The sample characteristics of the dMBI-PI group and control group were balanced at baseline (Table 2). As shown in Figure 2, from time point 2 to time point 4, there were 14 (8.7%) participants at time point 2 (3, 1.9%, participants missed the assessment and 11, 6.9%, dropped out of the study); 14 (8.7%) participants at time point 3 (dropped out of the study); and 28 (17.5%) participants at time point 4 (dropped out of the study) who did not respond to the assessments. With some exceptions (eg, time point 2 assessment noncompleters were more likely to report adverse obstetric histories and had a lower DISRS score at baseline; time point 3 assessment noncompleters had a shorter WASO and a lower DISRS score at baseline), there were no statistically significant differences in most baseline characteristics and intervention assignment among the time point 2, time point 3 or time point 4 assessment completers and noncompleters (Multimedia Appendix 4). Furthermore, the correlation analysis showed that there were small to moderate correlations among the primary outcome, secondary outcomes, and hypothesized mediators at baseline (all correlation coefficients were <0.70; Multimedia Appendix 5), indicating both reasonable overlap and independence among these variables.

**Table 2:** Participant baseline characteristics by intervention assignment.

Baseline characteristics				Total (N=160)		dMBI-PI^a^ and TAU^b^ (n=80)		TAU (n=80)		*P* value	
**Demographic information**											
	Age (y), mean (SD)			30.54 (3.86)		30.08 (4.00)		31.01 (3.69)		.12	
	**Race, n (%)**										.99
		Han	152 (95)		76 (95)		76 (95)				
		National minority	8 (5)		4 (5)		4 (5)				
	**Education, n (%)**										.05
		Junior college or less	45 (28.1)		28 (35)		17 (21.3)				
		Bachelor degree or above	115 (71.9)		52 (65)		63 (78.8)				
	**Marital status, n (%)**										.12
		Married	156 (97.5)		76 (95)		80 (100)				
		Unmarried (cohabitation)	4 (2.5)		4 (5)		0 (0)				
	**Per capita monthly household income (RMB^c^ 1 yuan=US $0.1368), n (%)**										.35
		<3500	11 (6.9)		7 (8.8)		4 (5)				
		≥3500	149 (93.1)		73 (91.3)		76 (95)				
	**Living area, n (%)**										.05
		Urban	150 (93.8)		72 (90)		78 (97.5)				
		Rural	10 (6.3)		8 (10)		2 (2.5)				
	**Work status, n (%)**										.09
		Unemployed	19 (11.9)		13 (16.3)		6 (7.5)				
		Employed	141 (88.1)		67 (83.8)		74 (92.5)				
	**Prepregnancy sleep quality, n (%)**										.50
		Good	108 (67.5)		52 (65)		56 (70)				
		Poor	52 (32.5)		28 (35)		24 (30)				
	Prepregnancy BMI (kg/m^2^), mean (SD)			22.79 (3.99)		22.92 (4.5)		22.67 (3.43)		.70	
	BMI at the time of participation (kg/m^2^), mean (SD)			23.42 (4.06)		23.73 (4.60)		23.12 (3.43)		.34	
**Pregnancy-related information**											
	Gestational age (wk), mean (SD)			15.59 (2.89)		15.86 (2.86)		15.32 (2.91)		.24	
	**Mode of pregnancy, n (%)**										.99
		Natural conception	158 (98.8)		79 (98.7)		79 (98.7)				
		Assisted reproduction	2 (1.3)		1 (1.3)		1 (1.3)				
	**Gravidity^d^, n (%)**										.89
		1	79 (49.4)		38 (47.5)		41 (51.2)				
		2	42 (26.3)		22 (27.5)		20 (25)				
		≥3	39 (24.4)		20 (25)		19 (23.8)				
	**Parity^e^, n (%)**										.24
		Primipara^f^	105 (65.6)		56 (70)		49 (61.3)				
		Multipara^g^	55 (34.4)		24 (30)		31 (38.8)				
	**Adverse obstetric history, n (%)**										.99
		No	116 (72.5)		58 (72.5)		58 (72.5)				
		Yes	44 (27.5)		22 (27.5)		22 (27.5)				
	**Pregnancy complications, n (%)**										.38
		Yes	43 (26.9)		19 (23.8)		24 (30)				
		No	117 (73.1)		61 (76.3)		56 (70)				
	**Planned pregnancy, n (%)**										.60
		Yes	117 (73.1)		60 (75)		57 (71.3)				
		No	43 (26.9)		20 (25)		23 (28.7)				
**Primary outcome**											
	ISI^h^, mean (SD)			10.44 (2.65)		10.84 (2.9)		10.04 (2.33)		.06	
**Secondary outcomes**											
	**Subjective sleep patterns**										
		SOL^i^ (min), median (P25-P75)	27.86 (17.98-41.71)		25.77 (17.32-38.75)		29.23 (18.72-44.82)		.40		
		WASO^j^ (min), median (P25-P75)	10.71 (5.71-20)		10.36 (5.00-19.26)		10.93 (6.67-21.82)		.47		
		TST^k^ (h), mean (SD)	7.97 (0.85)		7.96 (0.78)		7.98 (0.92)		.91		
		Sleep efficiency (%), mean (SD)	0.86 (0.07)		0.85 (0.08)		0.86 (0.06)		.63		
	**Subjective sleep quality**										
		PSQI^l^, mean (SD)	8.33 (2.64)		8.58 (2.76)		8.09 (2.51)		.25		
	**Daytime consequences**										
		FFS^m^, mean (SD)	10.21 (3.88)		9.85 (3.65)		10.58 (4.09)		.24		
		ESS^n^, mean (SD)	9.49 (5.09)		9.29 (5.08)		9.69 (5.12)		.62		
	**Mental health outcomes**										
		GAD-7^o^, mean (SD)	6.29 (3.05)		6.30 (3.58)		6.29 (2.42)		.98		
		EPDS^p^, mean (SD)	9.14 (4.75)		8.58 (4.63)		9.70 (4.63)		.13		
**Hypothesized mediators**											
	DISRS^q^, mean (SD)			36.71 (9.77)		35.58 (10.12)		37.84 (9.34)		.14	
	APSQ^r^, mean (SD)			50.40 (22.56)		50.40 (23.25)		50.4 (22)		.99	
	PSAS^s^, mean (SD)			30.49 (7.83)		30.20 (7.79)		30.79 (7.91)		.64	
	SAMI-B^t^, mean (SD)			20.75 (5.55)		20.84 (6.18)		20.66 (4.88)		.84	
	SRBQ^u^, mean (SD)			38.20 (18.77)		36.50 (19.18)		39.9 (18.32)		.25	


^a^dMBI-PI: digital mindfulness-based intervention for prenatal insomnia symptoms.

^b^TAU: treatment as usual.

^c^RMB: renminbi.

^d^Refers to the total number of pregnancies women have experienced up to now, including the current pregnancy and any previous pregnancies that did not result in a live birth (such as miscarriages).

^e^Refers to the number of times women have given birth to liveborn infants up to now.

^f^Women who have not yet experienced childbirth.

^g^Women who have had one or more deliveries.

^h^ISI: Insomnia Severity Index.

^i^SOL: sleep onset latency.

^j^WASO: wake after sleep onset.

^k^TST: total sleep time.

^l^PSQI: Pittsburgh Sleep Quality Index.

^m^FFS: Flinders Fatigue Scale.

^n^ESS: Epworth Sleepiness Scale.

^o^GAD-7: Generalized Anxiety Disorder-7.

^p^EPDS: Edinburgh Postnatal Depression Scale.

^q^DISRS: Daytime Insomnia Symptom Response Scale.

^r^APSQ: Anxiety and Preoccupation about Sleep Questionnaire.

^s^PSAS: Pre-Sleep Arousal Scale.

^t^SAMI-B: brief version of the Sleep-Associated Monitoring Index.

^u^SRBQ: Sleep-Related Behaviors Questionnaire.

### Effects on the Primary Outcome and Secondary Outcomes

As shown in Table 3 (Multimedia Appendix 6), the total ISI score decreased by 5.38 and 3.37 (all *P*<.001) in the dMBI-PI group and the control group from time point 1 to time point 2, respectively. The reduction in the ISI score from time point 1 to time point 2 was significantly greater in the dMBI-PI group than in the control group (mean between-group difference –2.02, 95% CI –3.42 to –0.62; *P*=.005; Cohen *d*=0.46, 95% CI 0.01-0.92). From time point 1 to time point 3, the reductions for the total ISI score in the dMBI-PI group and control group were 4.45 and 2.43, respectively, with a significantly greater decrease for participants receiving the dMBI-PI (mean between-group difference –2.02, 95% CI –3.42 to –0.61; *P*=.005; Cohen *d*=0.46, 95% CI 0.01-0.92). However, from time point 1 to time point 4, the reductions for the total ISI score in the two groups did not differ from each other (mean between-group difference –1.12, 95% CI –2.57 to 0.33; *P*=.13; Cohen *d*=0.12, 95% CI –0.35 to 0.60). The remission rates were relatively steady between time point 2 and time point 3 in the dMBI-PI group (54/72, 68% and 50/73, 63%) but showed a decrease from 56% (45/74) at time point 2 to 43% (34/73) at time point 3 in the control group. Similarly, the rates of participants who had an ISI decrease of at least 3 points (achieving reliable change) were relatively steady by time point 2 and time point 3 in the dMBI-PI group (53/72, 66% and 50/73, 63%) but showed a decrease from 51% (41/74) at time point 2 to 36% (29/73) at time point 3 in the control group. Logistic regression analysis showed that there were no statistically significant differences in the remission rates and reliable change rates between the two groups at time point 2, but at time point 3, participants in the intervention group were significantly associated with the increased likelihood of achieving insomnia remission or reliable change, even after the FDR correction. At time point 4, the remissions rates and reliable change rates in both groups were largely decreased compared with those at time point 2, and no significant between-group differences were observed.

Although both the intervention group and control group had significant improvements in most secondary outcomes during the study period (eg, in WASO, PSQI, FFS, GAD-7, and EPDS from time point 1 to time point 2, or in PSQI, FFS, GAD-7, and EPDS from time point 1 to time point 3), and only the intervention group showed significant improvements in TST, SE, and ESS from time point 1 to time point 2, no significant group-by-time interaction effects were observed in most secondary outcomes except for SE and PSQI. From time point 1 to time point 2, the improvement in SE was significantly greater in the intervention group compared with the control group (mean between-group difference 0.02, 95% CI 0-0.04; *P*=.04); however, the difference was nonsignificant after performing FDR correction. In addition, participants in the dMBI-PI group had larger improvements in sleep quality than those in the control group from time point 1 to time point 2 (mean between-group difference –1.47, 95% CI –2.57 to –0.37; *P*=.009) and from time point 1 to time point 3 (mean between-group difference –1.30, 95% CI –2.39 to –0.21; *P*=.02), but only the between-group difference from time point 1 to time point 3 survived after FDR correction.

Similar results were obtained when we performed repeated analyses using imputed datasets (Multimedia Appendix 7), complete cases analysis (Multimedia Appendix 8), or as-treated analysis (Multimedia Appendix 9).

**Table 3:** Mixed-effects analysis of changes in primary and secondary outcomes from baseline to follow-up.

Measure	Change from time 1 to time 2 (between-group difference)				Change from time 1 to time 3 (between-group difference)				Change from time 1 to time 4 (between-group difference)		
	β (95% CI)^a^/odds ratio (95% CI)^b^	*P* value	Adjusted *P* value^c^	β (95% CI)^a^/odds ratio (95% CI)^b^		*P* value	Adjusted *P* value^c^	β (95% CI)^a^/odds ratio (95% CI)^b^		*P* value	Adjusted *P* value^c^
Primary outcome: ISI^d^ scores	–2.02 (–3.42 to –0.62)^a^	.005	—^e^	–2.02 (–3.42 to –0.61)^a^		.005	—	–1.12 (–2.57 to 0.33)^a^		.13	—
Secondary outcome: rate of remission from insomnia symptoms	1.68 (0.87 to 3.22)^b^	.12	.30	2.31 (1.21 to 4.39)^b^		.01	.04	1.18 (0.61 to 2.26)^b^		.62	.94
Secondary outcome: rate of achieving reliable change in ISI scores	1.59 (0.80 to 3.13)^b^	.19	.34	2.64 (1.35 to 5.16)^b^		.004	.03	1.48 (0.78 to 2.82)^b^		.23	.54
Secondary outcome: SOL^f^ (mins)	–3.52 (–12.12 to 5.08)^a^	.42	.47	—		—	—	—		—	—
Secondary outcome: WASO^g^ (mins)	–3.31 (–7.66 to 1.04)^a^	.14	.30	—		—	—	—		—	—
Secondary outcome: TST^h^ (h)	0.09 (–0.15 to 0.34)^a^	.47	.47	—		—	—	—		—	—
Secondary outcome: sleep efficiency (%)	0.02 (0.00 to 0.04)^a^	.04	.22	—		—	—	—		—	—
Secondary outcome: PSQI^i^	–1.47 (–2.57 to –0.37)^a^	.009	.10	–1.30 (–2.39 to –0.21)^a^		.02	.047	–1.12 (–2.25 to 0.01)^a^		.053	.25
Secondary outcome: FFS^j^	–0.87 (–2.28 to 0.53)^a^	.23	.35	–0.58 (–1.99 to 0.82)^a^		.42	.49	–0.05 (–1.50 to 1.40)^a^		.94	.94
Secondary outcome: ESS^k^	–0.79 (–2.36 to 0.78)^a^	.32	.40	-0.88 (–2.45 to 0.69)^a^		.27	.38	–0.15 (–1.79 to 1.48)^a^		.86	.94
Secondary outcome: GAD-7^l^	–0.96 (–1.97 to 0.04)^a^	.06	.22	–0.86 (–1.87 to 0.14)^a^		.09	.16	–0.95 (–1.99 to 0.08)^a^		.07	.25
Secondary outcome: EPDS^m^	–0.89 (–2.49 to 0.71)^a^	.28	.38	–0.50 (–2.10 to 1.09)^a^		.54	.54	–0.06 (–1.71 to 1.59)^a^		.94	.94

^a^Estimated between-group differences in changes in Insomnia Severity Index scores over time (group-by-time interactions) from the mixed-effects linear regression model.

^b^Estimated between-group differences in the likelihood of remission or achieving reliable change from the logistic regression model (the Insomnia Severity Index score at baseline was included as a covariate), with dropouts defined as no remission and no reliable change, respectively.

^c^*P* value after controlling for multiple testing due to multiple secondary outcomes using the Benjamini-Hochberg false discovery rate correction.

^d^ISI: Insomnia Severity Index.

^e^Not applicable.

^f^SOL: sleep onset latency.

^g^WASO: wake after sleep onset.

^h^TST: total sleep time.

^i^PSQI: Pittsburgh Sleep Quality Index.

^j^FFS: Flinders Fatigue Scale.

^k^ESS: Epworth Sleepiness Scale.

^l^GAD-7: Generalized Anxiety Disorder-7.

^m^EPDS: Edinburgh Postnatal Depression Scale.

### Intervention Engagement, Use of Nonstudy Treatments, and Adverse Events

Participants randomized to receive the dMBI-PI completed a mean of 2.66 (SD 2.23) modules of 6 total modules, with 65 of 80 participants (81%) completing at least one module, 37 (46%) participants completing at least 3 modules (adherent participants), and 15 (19%) participants completing all modules. Although no statistically significant differences were observed, when compared to nonadherent participants, adherent participants tended to report partial remission from clinical insomnia symptoms or complete remission rather than persistent subthreshold insomnia symptoms or clinical insomnia symptoms or subthreshold to clinical insomnia symptoms during most of the study period; they also tended to report reliable improvement rather than deterioration (Multimedia Appendix 10). Logistic regression analysis showed that, after adjusting for other baseline characteristics, pregnant women with a bachelor degree or higher education demonstrated a significantly higher likelihood of adhering to the intervention. We did not observe significant associations between other baseline characteristics and intervention adherence (Multimedia Appendix 11). There were no significant differences in the use of nonstudy treatments between the intervention and control group (no participant vs 1 participant reported using prescribed sleep medication, respectively; 2 participants vs 2 participants reported receiving massages, respectively). One participant in the control group experienced adverse events (induced labor due to fetal heart development problems); this was irrelevant to study participation because it occurred during the first week of the intervention and the participant received routine care.

### Effects on Hypothesized Mediators and the Mediation Analyses

Compared with the control group, participants receiving the dMBI-PI had significantly greater reductions in APSQ (mean between-group difference –11.64, 95% CI –19.32 to –3.96; *P*=.003), PSAS (mean between-group difference –2.59, 95% CI –4.83 to –0.35; *P*=.03), SAMI-B (mean between-group difference –2.26, 95% CI –4.23 to –0.29; *P*=.03) and SRBQ (mean between-group difference –7.27, 95% CI –13.29 to –1.25; *P*=.02) from baseline to postintervention study, even after the adjustment of multiple testing using the FDR correction (Table 4). Most of these results remained robust in the sensitive analysis (Multimedia Appendices 12-14).

**Figure 3:**
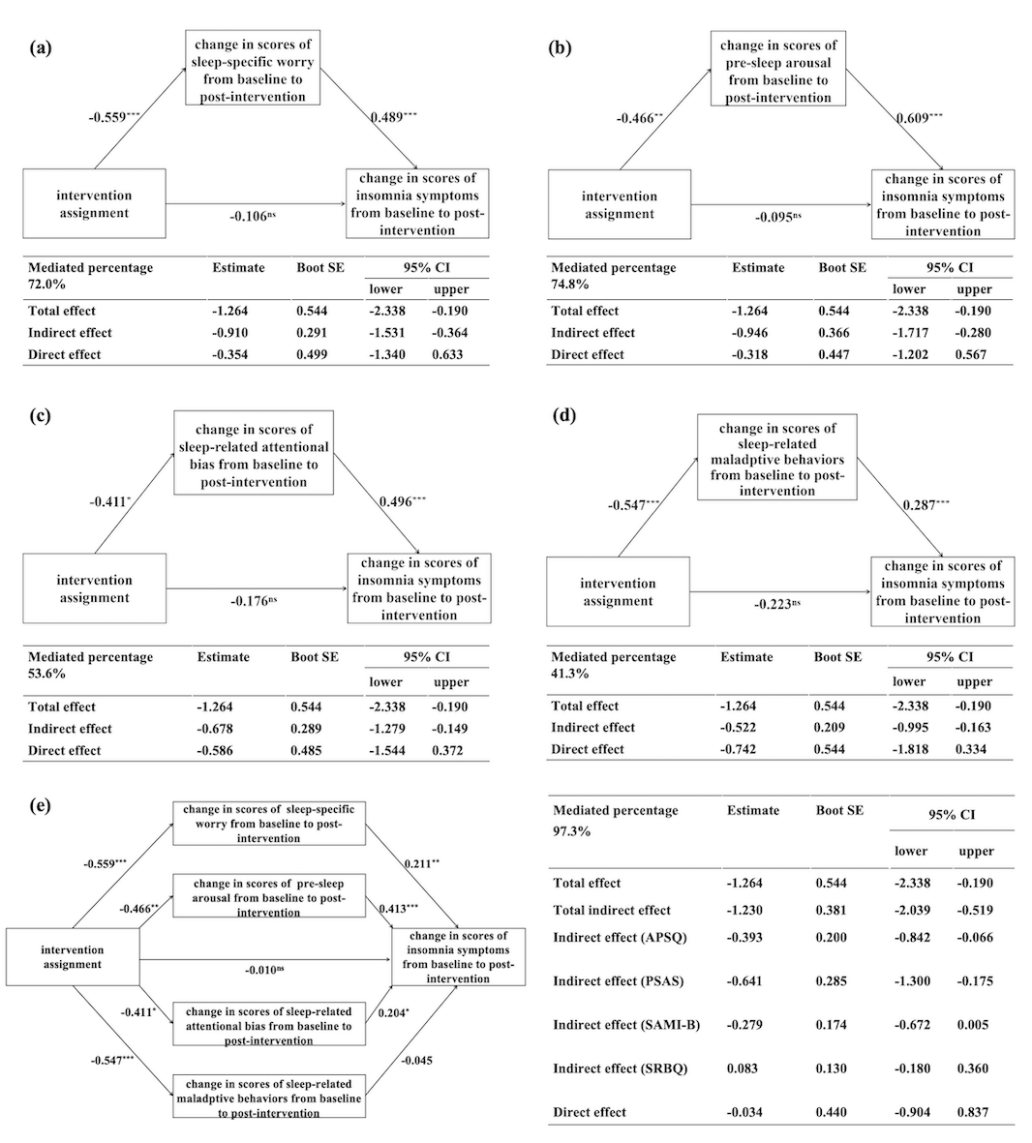
The mediation effects of adverse sleep-related cognitive and behavioral factors (residualized change scores from baseline to the end of the intervention) on the relationship between treatment assignment and residualized change scores in Insomnia Severity Index from baseline to the end of the intervention. *P<.05; **P<.01; ***P<.001; ns: statistically not significant; APSQ: Anxiety and Preoccupation About Sleep Questionnaire; PSAS: Pre-Sleep Arousal Scale; SAMI-B: brief version of the Sleep-Associated Monitoring Index; SRBQ: Sleep-Related Behaviors Questionnaire.

Furthermore, single mediation analyses showed that improvements in the APSQ, PSAS, SAMI-B, and SRBQ from time point 1 to time point 2 were significant mediators for the effect of intervention assignment on insomnia symptoms (Figure 3); they accounted for 72%, 74.8%, 53.6%, and 41.3% of the total intervention effect, respectively. In the multiple mediation analyses, the mediating effects of the APSQ and PSAS remained significant and the total indirect effect accounted for 97.3% of the dMBI-PI’s effect on insomnia symptoms. When we fitted mediation analyses using raw difference scores from time point 1 to time point 2, similar results were obtained except that the mediating effect of the SRBQ was nonsignificant in the single mediation analyses (Multimedia Appendix 15). Meanwhile, when we considered the temporal associations between the hypothesized mediators and outcome variable, the results showed that the indirect pathways of the intervention assignment linked to insomnia symptoms at time point 3 through the APSQ, PSAS, SAMI-B, and SRBQ at time point 2 were statistically significant, although the intervention effect on insomnia symptoms at time point 3 was nonsignificant (Multimedia Appendix 16).

**Table 4:** Mixed-effects analysis of change in hypothesized mediators from baseline to the end of the intervention.

Measure			Values, mean (SE)^a^				Within-group^b^					Between-group difference^c^							
			Time 1 (baseline)		Time 2 (postintervention study)		Change in score		*P* value		*β* (95% CI)				*P* value			Adjusted *P* value^d^	
**DISRS^e^**													–2.29 (–5.16 to 0.58)			.12			.12
	dMBI-PI^f^ and TAU^g^	35.60 (1.05)		29.00 (1.09)		–6.53		<.001											
	TAU	37.80 (1.05)		33.60 (1.08)		–4.24		<.001											
**APSQ^h^**													–11.64 (–19.32 to –3.96)			.003			.02
	dMBI-PI and TAU	50.40 (2.51)		32.30 (2.62)		–18.12		<.001											
	TAU	50.40 (2.51)		43.90 (2.59)		–6.48		.02											
**PSAS^i^**													–2.59 (–4.83 to –0.35)			.03			.03
	dMBI-PI and TAU	30.20 (0.86)		25.20 (0.90)		–4.98		<.001											
	TAU	30.80 (0.86)		28.40 (0.89)		–2.39		.004											
**SAMI-B^j^**													–2.26 (–4.23 to –0.29)			.03			.03
	dMBI-PI and TAU	20.80 (0.62)		16.40 (0.65)		–4.45		<.001											
	TAU	20.70 (0.62)		18.50 (0.65)		–2.19		.003											
**SRBQ^k^**													–7.27 (–13.29 to –1.25)			.02			.03
	dMBI-PI and TAU	36.50 (2.07)		28.70 (2.16)		–7.82		<.001											
	TAU	39.90 (2.07)		39.40 (2.13)		–0.54		.80											

^a^Mean (SE) presented is least squares mean (SE) from the mixed-effects linear regression model.

^b^Estimated within-group change and *P* value from the mixed-effects linear regression model.

^c^Estimated between-group differences in changes in Insomnia Severity Index scores over time (group-by-time interactions) from the mixed-effects linear regression model.

^d^*P* value after controlling for multiple testing due to multiple hypothesized mediators using the Benjamini-Hochberg false discovery rate correction.

^e^DISRS: Daytime Insomnia Symptom Response Scale.

^f^dMBI-PI: digital mindfulness-based intervention for prenatal insomnia.

^g^TAU: treatment as usual.

^h^APSQ: Anxiety and Preoccupation about Sleep Questionnaire.

^i^PSAS: Pre-Sleep Arousal Scale.

^j^SAMI-B: brief version of the Sleep-Associated Monitoring Index.

^k^SRBQ: Sleep-Related Behaviors Questionnaire.

## Discussion

### Principal Findings

To our knowledge, this is the first published RCT with a relatively large sample size to examine the effectiveness of the dMBI-PI in reducing subthreshold to clinical insomnia symptoms in pregnant women and to explore whether the dMBI-PI achieved its effects through alleviating theoretically hypothesized mediators—adverse cognitive and behavioral processes. The findings of this study indicated that compared with pregnant women in the control group, those in the dMBI-PI group had significantly greater improvements in insomnia symptoms from baseline to the end of the intervention and 2 months after the intervention (approximately the third trimester), but a beneficial effect was not observed post partum. In secondary outcomes, this study found that pregnant women in the intervention group were significantly associated with the increased likelihood of achieving remission or reliable change in insomnia symptoms at the third trimester; however, we did not observe significant differences in the changes of SOL, WASO, TST, fatigue symptoms, daytime sleepiness, and anxiety and depressive symptoms over time between the intervention group and control group, except that the intervention group showed greater improvements in sleep efficiency from baseline to the end of the intervention and greater improvements in sleep quality from baseline to the end of the intervention and 2 months after the intervention (only the between-group difference in the changes of sleep quality from baseline to the third trimester remained significant after adjusting for multiple testing). In addition, consistent with the prior theoretical model for the effects of mindfulness practices on risk processes for the development and maintenance of sleep disturbances [11], our results indicated that the sleep-related adverse cognitive and behavioral processes (mainly sleep-specific worry and presleep arousal) played a significant intermediary role in the dMBI-PI’s effect on prenatal insomnia symptoms, which may provide important information for understanding how MBIs work for insomnia.

Overall, this study provided preliminary support for the short-term benefits of the dMBI-PI in promoting greater improvements in subthreshold to clinical insomnia symptoms from baseline to the end of the intervention and the third trimester, which were prevalent during pregnancy and may worsen as pregnancy progressed without timely intervention [1,79]. However, compared with the large effect size for sleep quality reported in a recent meta-analysis (included 5 RCTs about face-to-face MBIs) among general patients with insomnia [50], it is difficult to tell whether the small to moderate effect size for prenatal insomnia symptoms detected in our trial, especially the absence of beneficial effects on most secondary outcomes, was due to the limited treatment effects of the dMBI-PI or other reasons. The following are several possible explanations. First, to benefit more women at risk of insomnia and prevent the persistence or deterioration of insomnia symptoms with the pregnancy progressed (especially in the third trimester), we used a relative relaxed screening criterion rather than stringent diagnostic criteria to include pregnant women with subthreshold to clinical insomnia symptoms. However, limited space for improvement may increase participants’ chances of achieving insomnia remission in both a natural state or when receiving standardized care (as suggested by the fact that 45 of 74, 50%, women in the control group achieved remission at the end of the intervention, although the remission rate in this group decreased largely at the third trimester and was significantly lower than that in the intervention group), making it difficult to highlight the superiority of the dMBI-PI for prenatal insomnia symptoms and leading to floor effects. Second, it may be unrealistic to expect self-help interventions without therapist guidance to achieve the same treatment effects as face-to-face treatments. In fact, the small to moderate effect size of this study is slightly lower than but quite close to the moderate effect size (Hedges *g*=0.595) reported in a recent meta-analysis on eHealth interventions for the treatment of insomnia during pregnancy [80]. It also aligns with our earlier trial results, where we found smartphone-based MBIs could significantly improve prenatal depression symptoms with an effect size of 0.47 [34]. Third, as indicated by a previous systematic review of digital MBIs [81] and similar trials among perinatal women [34,42], adherence to digital interventions remained a thorny issue. Nonadherence may impair statistical power and lead to an underestimation of the intervention effects. In this study, only 46% (37/80) of the participants completed at least 3 modules and were considered as adherent, which is close to the 52.4% and 56.76% adherence rate (based on the same criteria) reported in studies by Sun et al [34] and Felder et al [42], respectively, about the effectiveness of digital MBIs on perinatal depressive symptoms. This lack of compliance, on the one hand, may be related to the small dose, once-a-week course reminder, and the related lack of supervision and incentives for continuous practices in this study; however, it may truly portray the application compliance of self-help MBIs under real-world conditions. On the other hand, it may be related to the fact that many participants reported subthreshold insomnia symptoms at baseline and for whom only a small dose of intervention was needed to achieve remission. Future studies are warranted to validate our findings in pregnant women diagnosed with insomnia, using more frequent practice reminders or other adherence promotion measures (eg, creating more engaging videos, shortening videos and daily practice lengths, incorporating game-like elements, providing human support via videoconferences, or increasing personalized feedback to foster motivation). Notably, consistent with previous research showing that educational attainment positively influences adherence to mobile health apps for the self-management of noncommunicable diseases [82], our study found that a higher education level significantly correlated with an increased likelihood of adherence to the dMBI-PI. It may be because highly educated pregnant women often possess better cognitive and technical skills, digital literacy levels, and social support systems and are therefore more likely to adhere to health-promoting behavioral changes. To promote better adherence for socioeconomically disadvantaged individuals, further digital psychological interventions should be tailored to meet the needs of lower-educated vulnerable groups (eg, using humorous, interesting, and easy-to-understand terminology to deliver intervention content, as well as adopting user-friendly interfaces). Overall, this study may be an important step in demonstrating that the dMBI-PI may hold promise as first-step, self-help treatment options for pregnant women at risk of insomnia, freeing up limited health care resources for pregnant women who urgently need high-intensity treatments.

It is important to note that this study failed to observe the benefits of the dMBI-PI on insomnia symptoms post partum. This might result from a lack of prodding and incentives for continued practice. It was unclear whether and to what extent participants in the intervention group would continue to adhere to mindfulness practices as we did not design to collect related information. However, given the advantages of digital interventions (which allow for repetitive practices), further research should be of great significance to elucidate the long-term effects of the dMBI-PI on insomnia symptoms post partum by adding a maintenance plan to ensure continued support and home practices in addition to the 6-week intensive intervention. In addition, the lack of long-term beneficial effects might also be related to the fact that the intervention was not tailored specifically for postpartum sleep challenges. Previous research suggested that for postpartum women, infant sleep patterns and feeding schedules may pose a major challenge to their sleep and lead to multiple nighttime awakenings and serious disruption of sleep opportunities, which may occur beyond their voluntary control [83]. This may underscore the need for future research to tailor digital MBIs specifically for postpartum women by incorporating psychological education on postpartum feeding or strategies for promoting the development of regular sleep-wake patterns of infants.

Our study reinforces and extends limited literature on the cross-sectional and longitudinal associations of adverse cognitive and behavioral processes with insomnia symptoms [84,85] and supports the theoretical hypothesis proposed by Shallcross et al [11] that adverse cognitive and behavioral processes can be targeted by MBIs and may represent the key mechanisms by which MBIs improve insomnia symptoms. Indeed, limited studies to date on the mediating roles of adverse cognitive and behavioral factors in the effects of insomnia psychological interventions have focused on CBT-I; there is scarce evidence to elucidate whether MBIs may also work on insomnia symptoms through counteracting these sleep-related adverse cognitive and behavioral processes. Although further research is needed to elucidate their respective mechanisms in improving psychosomatic health problems, our findings suggest possible conceptual differences between CBT-I and MBIs. Unlike CBT-I, which focuses on eliminating and changing sleep-related dysfunctional beliefs and maladaptive behaviors [86,87], mindfulness-based practices are aimed at cultivating nonjudging and accepting awareness to undesirable experiences of insomnia, encouraging participants to release strong attachments to solving sleep problems, and promoting a more flexible response to insomnia experiences rather than excessive worry and psychosomatic activation [11]. While beyond the scope of this study, it must be acknowledged that our study design did not allow us to examine to what degree intervention components (such as the mindful approach or behavioral strategies) were effective in improving insomnia symptoms and which adverse cognitive and behavioral factors were specific mechanisms targeted by these intervention components. Future studies are warranted to use more complex designs with more intensive assessments of hypothesized mediators and outcomes to delineate the temporal relationships and mechanisms through which specific intervention components contribute to changes in adverse cognitive and behavioral factors and ultimately lead to improvements in insomnia symptoms.

### Strengths and Limitations

Our study had several notable strengths, including the rigorous RCT design, relatively low dropout rate, long-term follow-up across the second trimester to post partum, and a thorough testing of mechanisms of change guided by the theoretical model. Our study also had a few limitations to be noted. First, the participants were relatively homogeneous; most lived in cities, were employed, and were highly educated. Second, insomnia symptoms were measured by the self-reported assessment tool rather than the clinical diagnostic interview and objective measures (eg, actigraphy); nevertheless, self-reported insomnia symptoms may still be clinically significant because they are robust predictors of perinatal health outcomes [88] and closely related to help-seeking behaviors. Third, we used a relatively relaxed screening criterion to include pregnant women with subthreshold to clinical insomnia symptoms. Their relatively higher likelihood of insomnia remission in a natural state or when receiving standardized care and their lower motivation to adhere to the intervention may bias our results toward the null hypothesis. However, given that insomnia symptoms may worsen as pregnancy progressed, identifying the first-step, self-help treatment options have significant implications for preventing or curbing the prevalence of insomnia symptoms in the third trimester (the most rapid and sensitive periods of fetal brain development). Fourth, as in previous similar trials for perinatal women [34,42], the relatively poor adherence in digital programs may impair statistical power and lead to an underestimation of the intervention effects. However, compliance was documented objectively and not susceptible to self-report bias; this study may document a relatively conservative, practical estimate of the intervention effect, with the limited adherence close to that in real-world conditions. More importantly, the main and sensitivity analyses robustly identified the benefits of the dMBI-PI from baseline to the end of the intervention and the third trimester. Fifth, in the exploratory mediation analysis, the theoretically hypothesized mediators and insomnia symptoms were assessed simultaneously; thus, it is difficult to tell whether the improvements in mediators preceded the improvement in insomnia—further research is required. Sixth, the exclusion criteria for concurrent mental disorders and sleep disorders were collected via self-reported rather than more validated measures like the Structured Clinical Interview for DSM-5, which may lead to misreporting; however, self-reported illness diagnosis has been widely used in epidemiological studies and validated for acceptable consistency with medical records. Seventh, although participants in the intervention group retained access to the intervention courses during follow-up, due to the initial idea of the study to determine treatment adherence based on the completion status of thematic courses or daily exercises at the end of the intervention, the WeChat miniprogram was designed with a rule that once participants completed the thematic courses or daily audio practices, the progress bar indicated that the goal has been completed. Even if they repeatedly watched the videos or followed the audio practices, the backend still showed the status as completed and did not record the number of times participants rewatched the courses or repeated the audio practices. This setup prevented us from directly obtaining objective data on participants’ continuous engagement in mindfulness practices after the intervention, which may affect estimates of the long-term effects of the dMBI-PI during pregnancy on postpartum outcomes.

### Conclusions

Our findings provided the first RCT evidence to support the short-term benefits of digital MBIs on prenatal insomnia symptoms from baseline to the end of the intervention and the third trimester. We also revealed potential mechanisms for how digital MBIs achieved their effects, specifically, through alleviating sleep-related adverse cognitive and behavioral processes (mainly sleep-specific worry and presleep arousal). Given that prenatal insomnia symptoms are widespread, debilitating, and largely untreated, this study may be of great clinical significance to provide a pragmatic and accessible option to help address the prevention and intervention dilemma using an existing, widely used app (WeChat) that allowed participants to access the intervention packages of MBIs anytime and anywhere by logging into WeChat on their smartphones or PCs and practice repeatedly when needed. Although it appeared to be less effective than therapist-supported CBT-I, digital MBIs may hold promise as a highly accessible and scalable first-step treatment option for pregnant women at risk of insomnia—critically, they can free up limited resources for pregnant women who urgently need high-intensity treatment. Further research is encouraged to validate the results of this trial and continue the investigations of other mechanisms of change as well as the moderators of the intervention effects to inform how to refine the intervention programs for the pregnant women who are most likely to benefit from the intervention, promoting a more personalized and precise treatment.

## Appendix

Multimedia Appendix 1 Study protocol.

Multimedia Appendix 2 Statistical analysis.

Multimedia Appendix 3 The hypothetical mediation models.

Multimedia Appendix 4 Baseline characteristics of participants who completed and who missed the assessments at each follow-up.

Multimedia Appendix 5 Correlations between primary outcome, secondary outcomes, and hypothesized mediators at baseline.

Multimedia Appendix 6 Mixed-effects analysis of changes in primary and secondary outcomes from baseline to follow-up.

Multimedia Appendix 7 Mixed-effects analysis of changes in primary and secondary outcomes from baseline to follow-up using imputed datasets.

Multimedia Appendix 8 Mixed-effects analysis of changes in primary and secondary outcomes from baseline to follow-up (complete cases analysis).

Multimedia Appendix 9 Mixed-effects analysis of changes in primary and secondary outcomes from baseline to follow-up (as-treated analysis).

Multimedia Appendix 10 Differences in the change status of insomnia symptoms during the study period between adherent and nonadherent participants.

Multimedia Appendix 11 Associations between baseline characteristics and intervention adherence.

Multimedia Appendix 12 Mixed-effects analysis of changes in hypothesized mediators from baseline to the end of the intervention using imputed datasets.

Multimedia Appendix 13 Mixed-effects analysis of changes in hypothesized mediators from baseline to the end of the intervention (complete cases analysis).

Multimedia Appendix 14 Mixed-effects analysis of changes in hypothesized mediators from baseline to the end of the intervention (as-treated analysis).

Multimedia Appendix 15 The mediation effects of adverse sleep-related cognitive and behavioral factors (raw difference scores from baseline to the end of the intervention) on the relationship between treatment assignment and raw difference scores in Insomnia Severity Index from baseline to the end of the intervention.

Multimedia Appendix 16 The mediation effects of adverse sleep-related cognitive and behavioral factors at the end of the intervention on the relationship between treatment assignment and insomnia symptoms at 2 months after the intervention.

Multimedia Appendix 17 CONSORT-eHEALTH checklist (V1.6.1).

## Abbreviations

APSQ: Anxiety and Preoccupation About Sleep Questionnaire
CBT-I: cognitive behavioral therapy for insomnia
DISRS: Daytime Insomnia Symptom Response Scale
dMBI-PI: digital mindfulness-based intervention targeted at prenatal insomnia
EPDS: Edinburgh Postnatal Depression Scale
ESS: Epworth Sleepiness Scale
FDR: false discovery rate
FFS: Flinders Fatigue Scale
GAD-7: Generalized Anxiety Disorder-7
ISI: Insomnia Severity Index
MBI: mindfulness-based intervention
MBT-I: mindfulness-based therapy for insomnia
PSAS: Pre-Sleep Arousal Scale
PSQI: Pittsburgh Sleep Quality Index
RCT: randomized controlled trial
SAMI-B: brief version of the Sleep-Associated Monitoring Index
SOL: sleep onset latency
SRBQ: Sleep-Related Behaviors Questionnaire
TST: total sleep time
WASO: wake after sleep onset

## Data Availability

The datasets used and analyzed during this study are available from the corresponding author on reasonable request.
