# Supplementary material for: Effectiveness and Mechanisms of a Digital Mindfulness–Based Intervention for Subthreshold to Clinical Insomnia Symptoms in Pregnant Women: Randomized Controlled Trial
Source: J Med Internet Res. 2025 May 5;27:e68084. doi: 10.2196/68084 (PMC12089866; doi:10.2196/68084)
Supplement: Multimedia Appendix 1 [file jmir_v27i1e68084_app1.doc]

**Study protocol**

**Project name:** The effectiveness and mechanisms of a digital mindfulness-based intervention on the treatment of prenatal insomnia symptoms: a randomized clinical trial

**Primary Investigator:** Fenglin Cao, PhD, Professor, School of Nursing and Rehabilitation, Shandong University

**Research Unit:** the School of Nursing and Rehabilitation, Shandong University

**Introduction**

Insomnia symptoms are common during pregnancy, with an estimated prevalence of 38.2% in a recent meta-analysis [1]. Although not all women with self-reported insomnia symptoms met the diagnostic criteria for the insomnia disorder, research has indicated that insomnia symptoms were associated with an increased risk of perinatal depression, low birth weight and impaired fetal neurodevelopment [2-4]. To date, research on interventions to improve perinatal insomnia symptoms are severely limited. As the guideline recommended first line treatment, the effectiveness of cognitive behavioral therapy for insomnia (CBT-I) among pregnant women has been documented by several recent randomized controlled trials (RCT) [5-7]. Regrettably, only 34.8% to 63.8% of pregnant women achieved insomnia remission in these trials [5-7]. Moreover, the availability of trained clinicians and cost have resulted in limited access for pregnant women, especially those experiencing subthreshold insomnia symptoms that cannot always be assumed to achieve remission without intervention [5]. Hence, identifying additional treatments for the treatment of subclinical and clinical prenatal insomnia symptoms is critical for protecting maternal and fetal well-being.

Recently, theoretical considerations and empirical evidence suggest that mindfulness-based interventions (MBIs) may offer one promising approach. As Shallcross proposed in the theoretical framework for mindfulness and sleep disturbance [8], the core principles of MBIs (present-moment awareness, non-judging and acceptance) may target each of the adverse cognitive and behavioral processes (e.g., rumination, worry, pre-sleep arousal, sleep-related attentional bias or maladaptive behaviors) associated with insomnia, and thus relieving insomnia symptoms. Burgeoning research in a variety of populations has begun to support the benefits of MBIs for the treatment of insomnia symptoms [9-11]. However, a literature search yielded only a limited number of pilot studies or small sample randomized controlled trials examining the effectiveness of MBIs on prenatal insomnia symptoms [12-14]. More adequately powered RCT studies are still warranted to provide more confirmative conclusions about the effectiveness of MBIs during pregnancy.

Considering the dissemination/access barriers prevalent in most in-person psychological treatments for insomnia (e.g., a shortage of professional practitioners, treatment cost, time and space restrictions), alternative delivery of MBIs is also an issue worthy of concern. In recent years, the worldwide expansion of instant message platforms has facilitated clinical innovations that leverage digital technologies to enhance the accessibility, scalability, or affordability of the MBIs. For example, one mobile social app (WeChat) has more than one billion monthly active users and is one of the most popular instant message services worldwide [15], with immense potential to impact public health. Preliminary clinical trials have demonstrated effectiveness for digital MBIs in improving sleep among non-pregnant populations [16-18]. For pregnant women, such digital delivery that maximizes flexibility may be of particular interest, as they (predominantly young people) have limited personal time off from work, great eHealth literacy [19], and for whom timely accessibility of intervention may be of particular importance. Nonetheless, few studies have established digital delivery of MBIs to improve insomnia symptoms during pregnancy. Only one published pilot RCT protocol planned to test the feasibility and effectiveness of digital MBIs for prenatal sleep outcomes [20]; and the results have not yet been reported.

In addition to efforts toward clarifying treatment benefits, research on treatment mechanisms is valuable for informing future refinement of treatment protocols and optimization of treatment outcomes. In the prior proposed conceptual models integrating theoretical model of insomnia with the principles and processes of MBIs, adverse cognitive and behavioral factors (e.g., rumination, worry, pre-sleep arousal, sleep-related attentional bias or maladaptive behaviors) seem to be among the most promising candidates for elucidating the processes by which MBIs lead to insomnia improvement [8, 21]. However, to date, limited studies on the mediating roles of adverse cognitive and behavioral factors in the effects of insomnia psychological interventions has focused on CBT-I; only one published RCT has tested the mediating roles of rumination and worry in the effectiveness of MBIs on insomnia symptoms among patients with breast cancer [22]. Little is known about whether MBIs could lead to significant changes in multiple adverse cognitive and behavioral factors among pregnant women and whether these changes could mediate MBIs’ treatment effects for prenatal insomnia symptoms.

Therefore, based on a WeChat mini program “Mom Sleep Well”, the present study aimed to conduct a rigorous RCT to explore the effectiveness of a digital mindfulness-based intervention tailored for prenatal insomnia symptoms (dMBI-PI). Further, this study would investigate whether the improvements in adverse cognitive and behavioral factors could mediate the dMBI-PI’s effect on prenatal insomnia symptoms.

**Research Objectives**

1. to examine the effectiveness of dMBI-PI on subthreshold to clinical insomnia symptoms among pregnant women;
2. to explore the effects of dMBI-PI on a series of secondary outcomes (including insomnia remission rates and reliable change rates, subjective sleep patterns, sleep quality, fatigue symptoms, daytime sleepiness, anxiety symptoms and depressive symptoms);
3. to explore whether the improvements in adverse cognitive and behavioral factors could mediate the dMBI-PI’ s effect on prenatal insomnia symptoms.

**Methods**

**Research design**

This study is a single-blind, two-parallel-armed RCT.

**Participant recruitment**

*Recruitment sites*

Participants were recruited from the obstetric outpatient departments of two tertiary hospitals in [Blinded for review].

*Recruitment methods*

- face-to-face contact with the researcher at the obstetric outpatient departments
- Referral from the obstetricians

*Inclusion criteria*

- Aged ≥ 18 years.
- Singleton pregnancy.
- 12-20 weeks of pregnancy.
- Junior high school education level or above.
- Regular obstetric examinations at the research hospital and planned delivery at the hospital.
- Insomnia Severity Index (ISI) score ≥ 8 (corresponding to sub-threshold to clinical insomnia symptoms).
- Have a mobile WeChat account and be able to use WeChat mini-programs to participate in research.
- Clear consciousness, normal understanding and language expression ability, and able to fill in questionnaires independently.
- Have not received non-drug treatment for insomnia (such as massage, acupuncture or psychological intervention, etc.) in the last 6 months and not on the waiting list for other interventions.

*Exclusion criteria*

- Severe physical or mental illness before or now.
- There are current prescription or non-prescription drugs that affect sleep such as sleeping class.
- Diagnosis of organic sleep disorders before pregnancy or now (such as obstructive sleep apnea syndrome, narcolepsy, restless legs syndrome, etc.).
- Currently working on shifts or night shifts.
- Suspected severe depressive symptoms (EPDS ≥ 19 points).
- Suicidal tendencies, with a score of ≥ 2 on the 10th item of EPDS.
- Patients with severe pregnancy complications or complications need to be hospitalized for miscarriage protection.
- Previous experience in mindfulness meditation.
- Refuse to participate in research.

**Interventions**

*dMBI-PI*

The dMBI-PI treatment protocol was adapted from the Mindfulness-Based Therapy for Insomnia (MBTI) manual developed by Jason C. Ong[23] and the Mindfulness Behavioral Cognitive Therapy (MBCT) manual devised by John Teasdale, Mark Williams, and Zindel Segal [24]. An experienced mindfulness researcher (professor) with a doctoral degree in mental health led the adaptation of the treatment content tailored to pregnant women. The primary components of dMBI-PI included general education about the prevalence of prenatal insomnia symptoms and the significance of early intervention; mindfulness practices of awareness and acceptance; the applications of mindfulness for coping with insomnia; and insomnia-related behavioral strategies (stimulus control, sleep restriction and sleep hygiene) conducted under the principles and framework of mindfulness [25]. The main modifications made to accommodate pregnant women were as follows: (1) tailored examples to pregnant women; (2) during the mindfulness exercises, participants were instructed to increase their awareness and acceptance of physical discomfort and fetal movement; (3) with reference to other perinatal behavioral intervention practices, the recommended minimum time in bed was 5.5 hours with sleep restrictions (rather than 5 hours in the general population) [5]; (4) consistent with prior perinatal intervention trials [26] and the critical interpretive synthesis towards the instructional design components for effective mindfulness-based interventions [27], weekly thematic sessions and daily home practices were shorten to 10-30 minutes. The dMBI-PI is delivered through 6 weekly course modules. Each course module consists of a video-based thematic course and six days of audio-based home practices (formal mindfulness practices, such as body scan, mindful breathing, sitting mediation, etc.); participants were also encouraged to adhere to informal mindfulness practices (e.g., mindful eating, walking or 3-minute breathing practices) and sleep-promoting behaviors daily. All intervention materials were accessed via a WeChat mini program called “Mom Sleep Well”. Six course modules would be unlocked successively after inputting the invitation code. For each module, the thematic course would be unlocked first, followed by the six day home practices in turn.

*Standardized care (treatment as usual [TAU])*

All participants received TAU provided by obstetric staff, including regular telephone follow-ups, health education delivered through pregnancy school (such as common physical and mental problems, fetal movement count, precautions for labor and delivery, postpartum breastfeeding, etc.), pregnancy risk assessment and management, and nutrition and lifestyle guidance (such as weight gain, exercise). There were no limits on the use of non-study treatments for participants.

**Research procedures**

- Potentially eligible pregnant women (between 12 and 20 weeks of gestation) were informed about the research by a researcher (an experienced outpatient assistant). Women interested in participating were invited to add the WeChat account of the researcher, provide the online informed consent for the screening procedures, and then complete a short questionnaire (including general information, the ISI, and EPDS) to assess their eligibility to participate.
- Eligible individuals were contacted within three days to confirm their willingness to participate, and directed to fill in a baseline assessment (T1) via an online survey platform (wjx.cn). Participants were also instructed to complete the online sleep diaries for seven consecutive days through the WeChat mini program.
- After collecting the informed consent and baseline assessments, the researcher required a randomized assignment from the independent researcher based on a pre-generated random sequence. Participants randomized into receiving dMBI-PI plus TAU were informed of the invitation codes and then invited to log in to the system and proceed to the six courses modules. The research team members sent weekly course reminders to them via WeChat (“Hello, mothers-to-be, Week X courses have begun. Please remember to complete the course and home practices on time”). To balance the potential biases due to the researcher contact, participants randomized into receiving TAU were also contacted weekly using a standardized WeChat message (“Hello, mothers-to-be, how have you been this week? Please remember to stay in a good mood and receive check-ups on time”).
- At post-intervention (T2), participants were sent a questionnaire battery including primary outcome, secondary outcome and potential mechanism measures. At two months after post-intervention (T3) and 42 days postpartum (T4), participants were requested to complete all study measures except the sleep diaries. To promote retention, all participants who completed the follow-up assessments were provided with free health consultation through WeChat or internet hospitals (mainly including the contents or precautions of routine antenatal examination, outpatient appointments, and the interpretation of inspection results).

**Randomization and blinding**

A researcher independent of the research team generated the random number sequence using the random number generators in the SPSS 25.0. The random number rankings were stored in numbered, sealed, and opaque envelops. When the eligible participants completed baseline assessments and provided informed consent, the independent researcher opened the envelope in the order of enrollment and informed the researcher team of the allocation assignment. Because the control group did not receive the same period of digital intervention courses, it was difficult to keep the treatment implementers and participants blinded to the treatment assignment. However, the advantages of standardization and high fidelity in the digital interventions and the use of online self-administered assessments for participants may potentially alleviate the consequences of unblinding to treatment implementers. The assignment was only concealed from the data collectors.

**Measures**

*Primary outcome*

- *Insomnia Severity Index (ISI)*

The 7-item ISI was conducted to assess the severity of self-reported insomnia symptoms over the past two weeks [28]. In prior research [5, 7, 29], the ISI has been validated as a reliable measure for perinatal insomnia symptoms, and a cutoff score of 8 and 11 has been widely adopted to identify participants suffering from sub-threshold and clinical insomnia symptoms, respectively. Correspondingly, a total score of less than 8 has been commonly used as the criterion for remission from insomnia symptoms.

*Secondary outcomes*

- *Consensus Sleep diary-Core*

The online Consensus Sleep Diary-Core (nested in the WeChat mini program) was used to assess the subjective sleep patterns, including the time of going to bed, the time of getting ready for sleep, time taken to fall asleep, duration of awakenings, the time of morning awakening, and rise time [30]. By averaging data across one week, sleep onset latency (SOL), wake after sleep onset (WASO), total sleep time (TST) and sleep efficiency (SE) were derived from the sleep diaries [31]. The SE (percentage) was calculated by dividing TST by time in bed (duration from going to bed to getting up) and then multiplying the result by 100.

- *Pittsburgh Sleep Quality Index (PSQI)*

The PSQI was used to assess self-reported sleep quality over the previous month [32]. It contains 7 components, and each component is rated from 0 to 3. The total score ranges from 0 to 21, with higher scores indicating poorer sleep quality.

- *Flinders Fatigue Scale (FFS)*

Fatigue symptom severity (over the past two weeks) was assessed using the FFS, which includes 7 items and is rated on a range from 0 to 31, with higher scores indicating more severe fatigue symptoms. This scale has been widely used in insomnia research and has displayed great psychometric properties and sensitivity to treatment responses [33, 34].

- *Epworth Sleepiness Scale (ESS)*

Daytime sleepiness was measured using the ESS. This scale comprises 8 items scored from 0 to 3 and the total score range from 0 to 24, with higher scores indicating greater daytime sleepiness [35].

- *Generalized Anxiety Disorder-7 (GAD-7)*

GAD-7 is a 7-item self-reported measure assessing the anxiety symptom severity, and each item is scored from 0 to 3 points. Total scores range from 0 to 21 points, with higher scores representing more severe anxiety symptoms [36, 37].

- *Edinburgh Postnatal Depression Scale (EPDS)*

Depressive symptom severity over the past one week was assessed via the EPDS. The scale contains 10 items with scores ranging from 0 to 3 points, with all item scores summed to create a total score ranging from 0 to 30 points. Participants with higher EPDS scores display more severe depressive symptoms [38, 39].

- *Pregnancy-Related Anxiety Questionnaire (PRAQ)*

The 13-item PRAQ was used to assess pregnancy-related anxiety symptoms. Each item is scored from 1 (“never worry”) to 4 (“always worry”) points. The total score ranges from 13 to 52 points, with higher scores representing higher levels of pregnancy-related anxiety [40].

*Potential mechanisms*

- *Daytime Insomnia Symptom Response Scale (DISRS)*

The DISRS was used to assess the sleep-specific ruminative tendencies in pregnant women experiencing insomnia symptoms [41]. The 20-item scale mainly queries how frequently participants may engage in repetitive thinking when feeling tired (e.g., “thinking about how hard it is to concentrate”, or “thinking about how tired you feel”). Each item is scored on a scale ranging from 1 (“Almost Never”) to 4 (“Almost Always”). A total score ranging from 20 to 80 is derived by adding all items, with higher scores indicating higher ruminative tendencies towards sleep disruption.

- *Anxiety and Preoccupation about Sleep Questionnaire (APSQ)*

The 10-item APSQ was conducted to measure the severity of sleep-specific worry (e.g., “I worry about my loss of control over sleep”) [42].The response alternatives for each item range from 1 (“Strongly Disagree”) to 10 (“Strongly Agree”) points, with the total score ranging from 10 to 100 points. Higher scores indicate more severe worry about sleep.

- *Pre-Sleep Arousal Scale (PSAS)*

The 16-item PSAS was conducted to capture the cognitive (e.g., “being mentally alert, active”) and physical arousal symptoms (e.g., “A tight feeling in muscles”) before falling asleep [43, 44]. Each item is rated on a five-point scale from 1 (“not at all”) to 5 (“extremely”) points, with the total scores ranging from 16 to 80 points. Higher scores indicate higher levels of pre-sleep arousal.

- *Brief Version of the Sleep-Associated Monitoring Index (SAMI-B)*

The 8-item SAMI-B was used to assess the tendency of selective attention and monitoring towards sleep-related threat cues [45]. Each item is scored from 1 (“not at all”) to 5 (“all the time”), and the total scores thus range from 8 to 40 points, with higher scores indicating higher levels of sleep-related attentional bias.

- *Sleep-Related Behaviors Questionnaire (SRBQ)*

The SRBQ is a 32-item self-reported measure assessing how frequently respondents engage in sleep-related safety behaviors to cope with disturbed sleep [46]. Each item is rated from 0 (“almost never”) to 4 (“almost always”) points. Total scores range from 0 to 128 points, with higher scores representing more frequent use of sleep-related maladaptive behaviors.

*Additional indicators*

- *Mindfulness Attention Awareness Scale (MAAS)*

The MAAS is a 15-item self-report scale that assesses the levels of mindfulness awareness [47, 48]. Each item is rated on a scale ranging from 1 (“always”) to 6 (“never”) points. Higher scores indicate greater levels of mindfulness awareness in daily life.

- *Dysfunctional Beliefs and Attitudes about Sleep Scale 16 version (DBAS-16)*

The DBAS-16 is a 16-item questionnaire designed to assess sleep-related dysfunctional cognitions [49]. Each item is rated from 1 (“strongly disagree”) to 5 (“strongly agree”) points. The total score range is thus 16 to 80 points, with a higher score in DBAS-16 indicating a more dysfunctional belief towards sleep.

*Adherence, Use of Non-study Treatments and Adverse Events*

- Adherence was assessed by the completion of thematic courses/home practices recorded in the back-end management system of the WeChat mini program. Referring to previous research among perinatal women [26, 50], a module was defined as completed when participants completed the thematic course or at least 3 days of mindfulness practices, and participants completed at least half (3/6) of the modules were considered adherent.
- At post-intervention, participants were asked to self-report their use of non-study insomnia-related treatments, such as prescribed sleep medication, or over-the-counter sleep aids (e.g., melatonin).
- Adverse events were gathered through emergency contact information provided by the research team and medical records.

**Sample size calculation**

In a previous meta-analysis, the mindfulness-based intervention group displayed a significantly greater improvement in insomnia (measured by the Pittsburgh Sleep Quality Index) than the control group, with a standardized mean difference of -1.01 (95% CI: -1.28 to -0.75) [51]. Considering the self-help nature of dMBI-PI, the medium effect of digital MBIs on prenatal depression symptoms observed in our previous study [26] and a similar or larger effect on insomnia symptoms observed in a prior RCT with small sample size [52], we conservatively estimated a moderate between-group difference in the mean change scores in ISI from baseline to post-intervention, the targeted recruitment sample was 80 in each condition (160 overall) based on a statistical power of 80%, a two-tailed 5% significance level and an anticipated study attrition of 20%.

**Ethical Consideration**

- This study protocol is subject to obtain approval from the Ethics Committee. All participants are required to provide electronic informed consent.
- For pregnant women who were excluded due to suspected severe depressive symptoms or active suicidality, the researcher informed their obstetricians and provided referral recommendations and assistance.
- This study did not place any limits on access to non-study treatments (including the use of sleep medication or non-drug treatments). At study completion, on the premise that the intervention was effective, participants in the control group were also provided access to dMBI-PI courses.

**Statistical analysis plan**

- The analyses were performed using SPSS version 26.0 and Stata 15.1 under the intention-to-treat (ITT) approach. A *p* value less than 0.05 (2-sided) indicated statistical significance.
- Differences in baseline characteristics (including general information, primary outcome, secondary outcomes, and potential mechanisms) between groups (the intervention group vs the control group; follow-up sample vs drop-out sample) were compared using independent sample *t* tests, Mann-Whitney U tests, or χ^2^ tests. In the analysis of intervention effects, baseline characteristics with significant differences between groups will be treated as covariates.
- Linear mixed-effects models were conducted to compare differences in changes in outcomes between participants in the intervention and control group. This method allowed us to included available data from pregnant women who missed the follow-up assessments at any time point [53]. These analyses included each outcome measure (primary outcome, secondary outcomes, or potential mechanisms) as dependent variables, time, intervention assignment, and their two-way interactions as the independent variables, along with random intercepts to accommodate the within-participant correlation of repeated responses; a significant time-by-group interaction indicated that there was significant group difference in the changes in outcomes. In addition, logistic regression models were fitted to assess whether the proportions of participants with remitted insomnia symptoms and achieving reliable change differed between two groups during follow-up.
- Effect sizes (Cohen *d*) were calculated by dividing the between-group differences at post-intervention or follow-up by the pooled standard deviations (SD) of the continuous outcomes at baseline, with 0.2, 0.5, and 0.8 corresponding to small, moderate or large effect sizes, respectively.
- Further, mediation models were fitted using the PROCESS in SPSS 26.0 to examine the extent to which the effect of dMBI-PI on prenatal insomnia symptoms was mediated by improved adverse cognitive and behavioral processes. Only adverse cognitive and behavioral factors that displayed significant differences in changes over time between the intervention and control groups were included. Using the intervention assignment as the independent variable, the change scores of adverse cognitive and behavioral factors from baseline to post-intervention as potential mediators, and the change scores of insomnia symptom severity from baseline to post-intervention as dependent variables, single mediation analyses were performed separately for each potential mediator. Then, multiple mediation analysis was performed to examine the parallel mediating effects of adverse cognitive and behavioral factors in the association between intervention assignment and improvement in prenatal insomnia symptoms. The bias-corrected bootstrapping approach (*n* = 5000) was used to test the 95% confidence interval (CI) of the indirect effect. This procedure was preferred since it did not require the normal distribution of the indirect effect, and was considered to provide a more accurate estimate for confidence intervals [54].

**References**

1. Sedov ID, Anderson NJ, Dhillon AK, Tomfohr-Madsen LM: Insomnia symptoms during pregnancy: A meta-analysis. J Sleep Res 2021, 30(1):e13207.

2. Chang JJ, Pien GW, Duntley SP, Macones GA: Sleep deprivation during pregnancy and maternal and fetal outcomes: Is there a relationship? Sleep Medicine Reviews 2010, 14(2):107-114.

3. Lu Q, Zhang X, Wang Y, Li J, Xu Y, Song X, Su S, Zhu X, Vitiello MV, Shi J et al: Sleep disturbances during pregnancy and adverse maternal and fetal outcomes: A systematic review and meta-analysis. Sleep Med Rev 2021, 58:101436.

4. Lavonius M, Railo H, Karlsson L, Wikström V, Tuulari JJ, Scheinin NM, Paavonen EJ, Polo-Kantola P, Karlsson H, Huotilainen M: Maternal sleep quality during pregnancy is associated with neonatal auditory ERPs. Scientific reports 2020, 10(1):7228.

5. Manber R, Bei B, Simpson N, Asarnow L, Rangel E, Sit A, Lyell D: Cognitive Behavioral Therapy for Prenatal Insomnia: A Randomized Controlled Trial. Obstet Gynecol 2019, 133(5):911-919.

6. Kalmbach DA, Cheng P, O'Brien LM, Swanson LM, Sangha R, Sen S, Guille C, Cuamatzi-Castelan A, Henry AL, Roth T et al: A randomized controlled trial of digital cognitive behavioral therapy for insomnia in pregnant women. Sleep Med 2020, 72:82-92.

7. Felder JN, Epel ES, Neuhaus J, Krystal AD, Prather AA: Efficacy of Digital Cognitive Behavioral Therapy for the Treatment of Insomnia Symptoms Among Pregnant Women: A Randomized Clinical Trial. JAMA Psychiatry 2020.

8. Shallcross AJ, Visvanathan PD, Sperber SH, Duberstein ZT: Waking up to the problem of sleep: can mindfulness help? A review of theory and evidence for the effects of mindfulness for sleep. Curr Opin Psychol 2019, 28:37-41.

9. Ong JC, Manber R, Segal Z, Xia Y, Shapiro S, Wyatt JK: A randomized controlled trial of mindfulness meditation for chronic insomnia. Sleep 2014, 37(9):1553-1563.

10. Wong SY, Zhang DX, Li CC, Yip BH, Chan DC, Ling YM, Lo CS, Woo DM, Sun YY, Ma H et al: Comparing the Effects of Mindfulness-Based Cognitive Therapy and Sleep Psycho-Education with Exercise on Chronic Insomnia: A Randomised Controlled Trial. Psychother Psychosom 2017, 86(4):241-253.

11. Rusch HL, Rosario M, Levison LM, Olivera A, Livingston WS, Wu T, Gill JM: The effect of mindfulness meditation on sleep quality: a systematic review and meta-analysis of randomized controlled trials. Ann N Y Acad Sci 2019, 1445(1):5-16.

12. Beddoe AE, Lee KA, Weiss SJ, Kennedy HP, Yang CPP: Effects of Mindful Yoga on Sleep in Pregnant Women: A Pilot Study. Biol Res Nurs 2010, 11(4):363-370.

13. Felder JN, Laraia B, Coleman-Phox K, Bush N, Suresh M, Thomas M, Adler N, Epel E, Prather AA: Poor Sleep Quality, Psychological Distress, and the Buffering Effect of Mindfulness Training During Pregnancy. Behav Sleep Med 2018, 16(6):611-624.

14. Field T, Diego M, Delgado J, Medina L: Tai chi/yoga reduces prenatal depression, anxiety and sleep disturbances. Complement Ther Clin 2013, 19(1):6-10.

15. Ding Y, Lu X, Xie Z, Jiang T, Song C, Wang Z: Evaluation of a Novel WeChat Applet for Image-Based Dietary Assessment among Pregnant Women in China. Nutrients 2021, 13(9).

16. Nourian M, Nikfarid L, Khavari AM, Barati M, Allahgholipour AR: The Impact of an Online Mindfulness-Based Stress Reduction Program on Sleep Quality of Nurses Working in COVID-19 Care Units: A Clinical Trial. 2021, 35(5):257-263.

17. Low T, Conduit R, Varma P, Meaklim H, Jackson ML: Treating subclinical and clinical symptoms of insomnia with a mindfulness-based smartphone application: A pilot study. Internet Interv 2020, 21:100335.

18. Wahbeh H: Internet Mindfulness Meditation Intervention (IMMI) Improves Depression Symptoms in Older Adults. Medicines (Basel, Switzerland) 2018, 5(4).

19. Sayakhot P, Carolan-Olah M: Internet use by pregnant women seeking pregnancy-related information: a systematic review. BMC Pregnancy Childbirth 2016, 16:65.

20. Kantrowitz-Gordon I, McCurry SM, Landis CA, Lee R, Wi D: Online prenatal trial in mindfulness sleep management (OPTIMISM): protocol for a pilot randomized controlled trial. Pilot and feasibility studies 2020, 6:128.

21. Ong JC, Smith CE: Using Mindfulness for the Treatment of Insomnia. Curr Sleep Med Rep 2017, 3(2):57-65.

22. Shao D, Zhang H, Cui N, Sun J, Li J, Cao F: The efficacy and mechanisms of a guided self-help intervention based on mindfulness in patients with breast cancer: A randomized controlled trial. Cancer 2021, 127(9):1377-1386.

23. Ong JC: Mindfulness-Based Therapy for Insomnia, 1st Edition edn. Washington: American Psychological Association; 2016.

24. Teasdale. J, Williams. M, Segal. Z: The mindful way workbook: An 8-week program to free yourself from depression and emotional distress. New York: Guilford Publications; 2014.

25. Edinger JD, Arnedt JT, Bertisch SM, Carney CE, Harrington JJ, Lichstein KL, Sateia MJ, Troxel WM, Zhou ES, Kazmi U et al: Behavioral and psychological treatments for chronic insomnia disorder in adults: an American Academy of Sleep Medicine systematic review, meta-analysis, and GRADE assessment. J Clin Sleep Med 2021, 17(2):263-298.

26. Sun Y, Li Y, Wang J, Chen Q, Bazzano AN, Cao F: Effectiveness of Smartphone-Based Mindfulness Training on Maternal Perinatal Depression: Randomized Controlled Trial. J Med Internet Res 2021, 23(1):e23410.

27. Lippmann M, Laudel H, Heinzle M, Narciss S: Relating Instructional Design Components to the Effectiveness of Internet-Based Mindfulness Interventions: A Critical Interpretive Synthesis. J Med Internet Res 2019, 21(11):e12497.

28. Bastien CH, Vallieres A, Morin CM: Validation of the Insomnia Severity Index as an outcome measure for insomnia research. Sleep Medicine 2001, 2(4):297-307.

29. Kalmbach DA, Cheng P, Roth A, Roth T, Swanson LM, O'Brien LM, Fresco DM, Harb NC, Cuamatzi-Castelan AS, Reffi AN et al: DSM-5 insomnia disorder in pregnancy: associations with depression, suicidal ideation, and cognitive and somatic arousal, and identifying clinical cutoffs for detection. Sleep advances : a journal of the Sleep Research Society 2022, 3(1):zpac006.

30. Carney CE, Buysse DJ, Ancoli-Israel S, Edinger JD, Krystal AD, Lichstein KL, Morin CM: The consensus sleep diary: standardizing prospective sleep self-monitoring. Sleep 2012, 35(2):287-302.

31. Natale V, Léger D, Bayon V, Erbacci A, Tonetti L, Fabbri M, Martoni M: The consensus sleep diary: quantitative criteria for primary insomnia diagnosis. Psychosom Med 2015, 77(4):413-418.

32. Buysse DJ, Reynolds CF, 3rd, Monk TH, Berman SR, Kupfer DJ: The Pittsburgh Sleep Quality Index: a new instrument for psychiatric practice and research. Psychiatry Res 1989, 28(2):193-213.

33. Gradisar M, Lack L, Richards H, Harris J, Gallasch J, Boundy M, Johnston A: The Flinders Fatigue Scale: preliminary psychometric properties and clinical sensitivity of a new scale for measuring daytime fatigue associated with insomnia. J Clin Sleep Med 2007, 3(7):722-728.

34. Cameron K, Williamson P, Short MA, Gradisar M: Validation of the Flinders Fatigue Scale as a measure of daytime fatigue. Sleep Med 2017, 30:105-112.

35. Johns MW: A new method for measuring daytime sleepiness: the Epworth sleepiness scale. Sleep 1991, 14(6):540-545.

36. Spitzer RL, Kroenke K, Williams JBW, Löwe B: A brief measure for assessing generalized anxiety disorder: the GAD-7. Arch Intern Med 2006, 166(10):1092-1097.

37. Simpson W, Glazer M, Michalski N, Steiner M, Frey BN: Comparative efficacy of the generalized anxiety disorder 7-item scale and the Edinburgh Postnatal Depression Scale as screening tools for generalized anxiety disorder in pregnancy and the postpartum period. Canadian journal of psychiatry Revue canadienne de psychiatrie 2014, 59(8):434-440.

38. Cox JL, Holden JM, Sagovsky R: Detection of Postnatal Depression - Development of the 10-Item Edinburgh Postnatal Depression Scale. Brit J Psychiat 1987, 150:782-786.

39. Wang Y, Guo X, Lau Y, Chan KS, Yin L, Chen J: Psychometric evaluation of the Mainland Chinese version of the Edinburgh Postnatal Depression Scale. Int J Nurs Stud 2009, 46(6):813-823.

40. Xiao L, Tao F, Zhang J, Hao J, Xu S, Wang H, Su P, Zhu P: Development and reliability evaluation of a pregnancy-related anxiety questionnaire. Chinese Journal of Public Health 2012, 28(3):275-277.

41. Carney CE, Harris AL, Falco A, Edinger JD: The Relation between Insomnia Symptoms, Mood, and Rumination about Insomnia Symptoms. Journal of Clinical Sleep Medicine 2013, 9(6):567-575.

42. Jansson-Fröjmark M, Harvey AG, Lundh L-G, Norell-Clarke A, Linton SJ: Psychometric Properties of an Insomnia-Specific Measure of Worry: The Anxiety and Preoccupation about Sleep Questionnaire. Cognitive Behaviour Therapy 2011, 40(1):65-76.

43. Nicassio PM, Mendlowitz DR, Fussell JJ, Petras L: The phenomenology of the pre-sleep state: the development of the pre-sleep arousal scale. Behaviour research and therapy 1985, 23(3):263-271.

44. Jan Y-W, Chen C-W, Yang C-M, Lin S-C: Validation of the Chinese Version of the Pre-Sleep Arousal Scale (PSAS). Archives of Clinical Psychology 2009, 4(1):51-58.

45. Jansson-Fröjmark M, Sunnhed R: Psychometric Properties of Two Brief Versions of Cognitive, Insomnia-Specific Measures: The Anxiety and Preoccupation About Sleep Questionnaire and the Sleep-Associated Monitoring Index. Psychol Rep 2020, 123(3):966-982.

46. Ree MJ, Harvey AG: Investigating safety behaviours in insomnia: The development of the sleep-related behaviours questionnaire (SRBQ). Behav Change 2004, 21(1):26-36.

47. Carlson LE, Brown KW: Validation of the Mindful Attention Awareness Scale in a cancer population. J Psychosom Res 2005, 58(1):29-33.

48. Deng Y-Q, Li S, Tang Y-Y, Zhu L-H, Ryan R, Brown K: Psychometric Properties of the Chinese Translation of the Mindful Attention Awareness Scale (MAAS). Mindfulness 2012, 3(1):10-14.

49. Chen C, Jan Y, Yang C, Lin S: Dysfunctional beliefs and atitudes about sleep (DBAS): validation of the Chinese version. Archives of Clinical Psychology 2009, 4(1):59-67.

50. Felder JN, Segal Z, Beck A, Sherwood NE, Goodman SH, Boggs J, Lemon E, Dimidjian S: An Open Trial of Web-Based Mindfulness-Based Cognitive Therapy for Perinatal Women at Risk for Depressive Relapse. Cognitive and Behavioral Practice 2017, 24(1):26-37.

51. Wang YY, Wang F, Zheng W, Zhang L, Ng CH, Ungvari GS, Xiang YT: Mindfulness-Based Interventions for Insomnia: A Meta-Analysis of Randomized Controlled Trials. Behav Sleep Med 2020, 18(1):1-9.

52. Kennett L, Bei B, Jackson ML: A Randomized Controlled Trial to Examine the Feasibility and Preliminary Efficacy of a Digital Mindfulness-Based Therapy for Improving Insomnia Symptoms. Mindfulness (N Y) 2021, 12(10):2460-2472.

53. Zhou ES, Ritterband LM, Bethea TN, Robles YP, Heeren TC, Rosenberg L: Effect of Culturally Tailored, Internet-Delivered Cognitive Behavioral Therapy for Insomnia in Black Women: A Randomized Clinical Trial. JAMA Psychiatry 2022, 79(6):538-549.

54. Hayes AF: Introduction to mediation, moderation, and conditional process analysis: A regression-based approach. New York, NY, US: Guilford Press; 2013.
