# Supplementary material for: Effectiveness and Mechanisms of a Digital Mindfulness–Based Intervention for Subthreshold to Clinical Insomnia Symptoms in Pregnant Women: Randomized Controlled Trial
Source: J Med Internet Res. 2025 May 5;27:e68084. doi: 10.2196/68084 (PMC12089866; doi:10.2196/68084)
Supplement: Multimedia Appendix 2 [file jmir_v27i1e68084_app2.doc]

**Statistical analysis**

All analyses were performed using SPSS version 25.0 and R version 4.2.2. Unless otherwise stated, a *p* value less than 0.05 (2-sided) indicated statistical significance. Independent sample *t* tests, *χ*^2^ tests, or Mann-Whitney U tests were conducted to examine whether there were differences in baseline characteristics (including general information, primary outcome, secondary outcomes, and hypothesized mediators) between the intervention group and control group, and between participants who completed and missed the follow-up assessments at each time point.

Following the intention-to-treat (ITT) principle, linear-mixed model analysis where we included group, time, and group-by-time interactions as the explanatory variables and random intercepts to account for the within-participant correlation of repeated responses, was conducted for ISI (primary outcome), a series of continuous secondary outcomes and hypothesized mediators separately. This method allowed us to included available data from pregnant women who missed the follow-up assessments at any time point, and the group-by-time interactions were used to measure the differences in within-participant outcome changes between the dMBI-PI intervention and control group [1]. Effect sizes (Cohen *d*) were calculated by dividing the between-group differences at post-intervention or follow-up by the pooled standard deviations (SD) of the continuous outcomes at baseline, with 0.2, 0.5, and 0.8 corresponding to small, moderate or large effect sizes, respectively [2]. For the secondary outcomes of remission and reliable change, logistic regression models (with dropouts defined as no remission and no reliable change, respectively) were fitted to assess whether the proportions of participants with remitted insomnia symptoms and achieving reliable change differed between two groups during follow-up. We corrected for the increased probability of type I error due to multiple testing of multiple secondary outcomes or hypothesized mediators using the Benjamini-Hochberg false discovery rate (FDR) correction. To assess the robustness of the results, a series of sensitivity analyses were conducted. First, to reduce the potential bias caused by missing data, multivariate imputation by chained equations (MCIE) was performed to generate 10 imputed datasets, and the main analyses were rerun using imputed datasets. Second, we reanalyzed the intervention effects of dMBI-PI by only including participants who completed all assessments (complete cases analysis), or who actually received the treatment (as-treated analysis). Third, to examine the relationship between the number of intervention modules completed and the changes of insomnia symptoms for pregnant women in the intervention group, we classified participants into four groups based on their insomnia symptom severity at baseline and follow-up (including post-intervention, the third trimester, and 42 days postpartum): partial remission from clinical insomnia symptoms (whose baseline ISI scores were ≥ 11 points, and the ISI scores at follow-up were ≥ 8 points but less than 11 points), complete remission (whose ISI scores at follow-up were less than 8 points), persistent sub-threshold or clinical insomnia symptoms (whose ISI scores at baseline and follow-up were both ≥ 11 points or ≥ 8 points), and progression from sub-threshold to clinical insomnia symptoms (whose baseline ISI scores were ≥ 8 points, but the ISI scores at follow-up were ≥ 11 points); similarly, we also classified participants into three groups based on whether their changes in scores of ISI from baseline to the follow-up met the criterion of reliable change: significant deterioration (whose ISI scores at follow-up increased by at least 3 points compared to baseline), no significant change (whose changes in scores of ISI from baseline to follow-up were within 3 points), and significant improvement (whose ISI scores at follow-up decreased by at least 3 points compared to baseline). Next, we examined whether there was a difference in the treatment effects (indicated by above remission status or reliable change status from baseline to follow-up) for adherent vs non-adherent participants in the intervention group through χ^2^ tests.

To examine the extent to which the dMBI-PI’s effect on perinatal insomnia symptoms was mediated by changing hypothesized mediators, potential eligible mediators showing significant differences in with-participant change between the dMBI-PI intervention and control group after the FDR correction were further included in single mediation models and a parallel multiple mediation model fitted using the PROCESS 3.3. To account for the confounding effects of baseline scores and possible random errors of measurement, the residualized change scores of ISI and hypothesized mediators (from baseline to post-intervention) obtained from the linear regression models were used to fit mediation models. Compared with the raw change scores (which are unconditional for baseline scores), the residualized change scores aim to capture the part of post-intervention scores that cannot be predicted by the baseline scores, which are statistically equivalent to using the post-intervention score as the outcome and including the baseline score as the covariate [3]. Sensitivity analyses using the raw change scores (measured by subtracting the post-intervention scores from baseline) were also performed to validate the robustness of results. To facilitate attempts to establish causal and temporal correlations, we also repeated the mediation analysis to examine whether the dMBI-PI’s effect on insomnia symptoms at T3 could be mediated by hypothesized mediators at T2. The bias-corrected bootstrapping (n = 5000) was used to test the 95% confidence interval (CI) of the indirect effect, and the mediating effect was considered as statistically significant if the 95% *CI* of the indirect effect did not contain 0.

**References**

1. Zhou ES, Ritterband LM, Bethea TN, Robles YP, Heeren TC, Rosenberg L. Effect of Culturally Tailored, Internet-Delivered Cognitive Behavioral Therapy for Insomnia in Black Women: A Randomized Clinical Trial. JAMA Psychiatry. 2022; 79(6):538-549. PMID: 35442432

2. Felder JN, Epel ES, Neuhaus J, Krystal AD, Prather AA. Efficacy of Digital Cognitive Behavioral Therapy for the Treatment of Insomnia Symptoms Among Pregnant Women: A Randomized Clinical Trial. JAMA Psychiatry. 2020; 77(5):484-492. PMID: 31968068

3. Valente MJ, MacKinnon DP. Comparing models of change to estimate the mediated effect in the pretest-posttest control group design. Struct Equ Modeling. 2017; 24(3):428-450. PMID: 28845097
