# Supplementary figures and images for: Effectiveness and Mechanisms of a Digital Mindfulness–Based Intervention for Subthreshold to Clinical Insomnia Symptoms in Pregnant Women: Randomized Controlled Trial
Source: J Med Internet Res. 2025 May 5;27:e68084. doi: 10.2196/68084 (PMC12089866; doi:10.2196/68084)

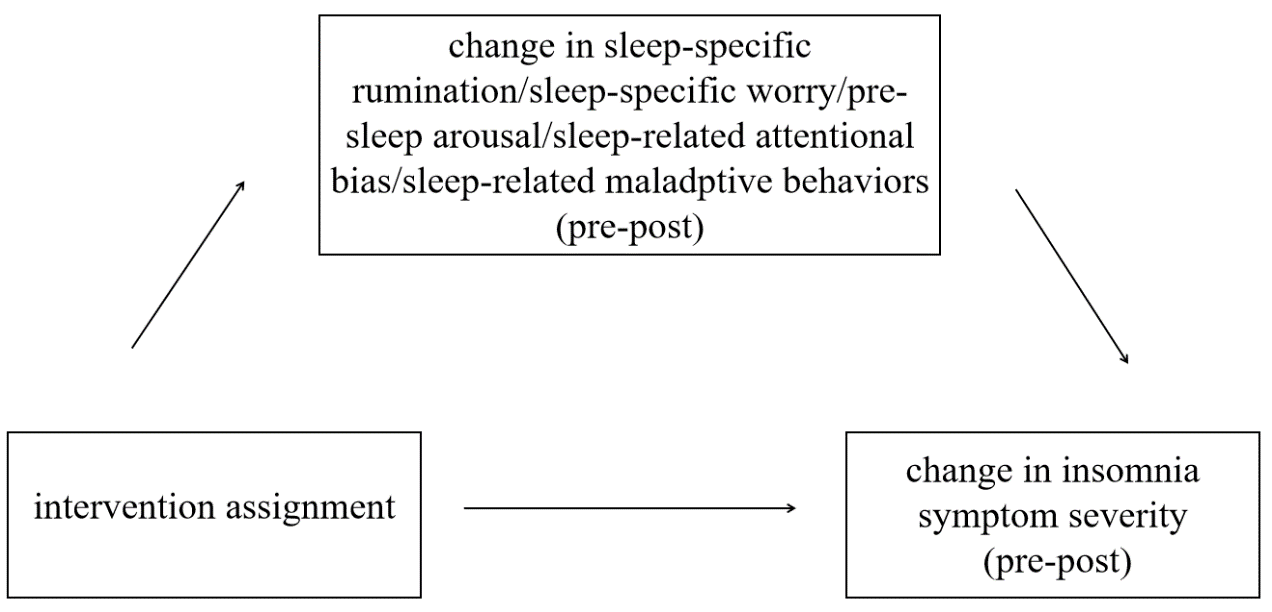


The hypothetical mediation models

Supplement: Multimedia Appendix 3 [file jmir_v27i1e68084_app3.doc]
