# Supplementary material for: Effectiveness and Mechanisms of a Digital Mindfulness–Based Intervention for Subthreshold to Clinical Insomnia Symptoms in Pregnant Women: Randomized Controlled Trial
Source: J Med Internet Res. 2025 May 5;27:e68084. doi: 10.2196/68084 (PMC12089866; doi:10.2196/68084)
Supplement: Multimedia Appendix 4 [file jmir_v27i1e68084_app4.doc]

Baseline characteristics of participants who completed and who missed the assessments at each follow-up

| Baseline characteristics | Time 2 (post-intervention) | | |  | Time 3 (two-months after post-intervention) | | |  | Time 3 (42 days postpartum) | | |
| --- | --- | --- | --- | --- | --- | --- | --- | --- | --- | --- | --- |
|  | Completers (n=146) | Noncompleters  (n=14) | *P* |  | Completers (n=146) | Noncompleters  (n=14) | *P* |  | Completers (n=132) | Noncompleters  (n=28) | *P* |
| **Intervention assignment** |  |  | 0.576 |  |  |  | 1.000 |  |  |  | 0.677 |
| dMBI-PI+TAU | 72 (49.3) | 8 (57.1) |  |  | 73 (50.0) | 7 (50.0) |  |  | 67 (50.8) | 13 (46.4) |  |
| TAU | 74 (50.7) | 6 (42.9) |  |  | 73 (50.0) | 7 (50.0) |  |  | 65 (49.2) | 15 (53.6) |  |
| **Demographic information** |  |  |  |  |  |  |  |  |  |  |  |
| **Age (years), mean (SD)** | 30.62±3.73 | 29.79±5.16 | 0.444 |  | 30.58±3.70 | 30.14±5.49 | 0.686 |  | 30.58±3.56 | 30.36±5.15 | 0.826 |
| **Race, n (%)** |  |  | 0.528 |  |  |  | 0.528 |  |  |  | 1.000 |
| Han | 139 (95.2) | 13 (92.9) |  |  | 139 (95.2) | 13 (92.9) |  |  | 125 (94.7) | 27 (96.4) |  |
| Minority | 7 (4.8) | 1 (7.1) |  |  | 7 (4.8) | 1 (7.1) |  |  | 7 (5.3) | 1 (3.6) |  |
| **Education, n (%)** |  |  | 0.539 |  |  |  | 1.000 |  |  |  | 0.325 |
| Junior college or less | 40 (27.4) | 5 (35.7) |  |  | 41 (28.1) | 4 (28.6) |  |  | 35 (26.5) | 10 (35.7) |  |
| Bachelor’s degree or above | 106 (72.6) | 9 (64.3) |  |  | 105 (71.9) | 10 (71.4) |  |  | 97 (73.5) | 18 (64.3) |  |
| **Marital Status, n (%)** |  |  | 1.000 |  |  |  | 0.309 |  |  |  | 0.540 |
| Married | 142 (97.3) | 14 (100.0) |  |  | 143 (97.9) | 13 (92.9) |  |  | 129 (97.7) | 27 (96.4) |  |
| Unmarried (cohabitation) | 4 (2.7) | 0 (0.0) |  |  | 3 (2.1) | 1 (7.1) |  |  | 3 (2.3) | 1 (3.6) |  |
| **Per capita monthly household Income (RMB), n (%)** |  |  | 1.000 |  |  |  | 0.248 |  |  |  | 0.409 |
| <3500 | 10 (6.81) | 1 (7.1) |  |  | 9 (6.2) | 2 (14.3) |  |  | 8 (6.1) | 3 (10.7) |  |
| ≥3500 | 136 (93.2) | 13 (92.9) |  |  | 137 (93.8) | 12 (85.7) |  |  | 124 (93.9) | 25 (89.3) |  |
| **Living area, n (%)** |  |  | 0.602 |  |  |  | 1.000 |  |  |  | 0.688 |
| Urban | 136 (93.2) | 14 (100.0) |  |  | 137 (93.8) | 13 (92.9) |  |  | 124 (93.9) | 26 (92.9) |  |
| Rural | 10 (6.8) | 0 (0.0) |  |  | 9 (6.2) | 1 (7.1) |  |  | 8 (6.1) | 2 (7.1) |  |
| **Work status, n (%)** |  |  | 0.674 |  |  |  | 0.221 |  |  |  | 0.747 |
| Unemployed | 17 (11.6) | 2 (14.3) |  |  | 16 (11.0) | 3 (21.4) |  |  | 15 (11.4) | 4 (14.3) |  |
| Employed | 129 (88.4) | 12 (85.7) |  |  | 130 (89.0) | 11 (78.6) |  |  | 117 (88.6) | 24 (85.7) |  |
| **Pre-pregnancy sleep quality, n (%)** |  |  | 1.000 |  |  |  | 0.772 |  |  |  | 0.689 |
| Good | 98 (67.1) | 10 (71.4) |  |  | 99 (67.8) | 9 (64.3) |  |  | 90 (68.2) | 18 (64.3) |  |
| Poor | 48 (32.9) | 4 (28.6) |  |  | 47 (32.2) | 5 (35.7) |  |  | 42 (31.8) | 10 (35.7) |  |
| **Pre-pregnancy BMI (kg/m^2^), mean (SD)** | 22.69±4.07 | 23.84±2.99 | 0.305 |  | 22.67±4.07 | 24.06±2.83 | 0.214 |  | 22.88±4.09 | 22.40±3.49 | 0.568 |
| **BMI at the time of participation (kg/m^2^), mean (SD)** | 23.31±4.14 | 24.61±2.98 | 0.252 |  | 23.29±4.14 | 24.78±2.87 | 0.193 |  | 23.43±4.16 | 23.38±3.59 | 0.955 |
| **Pregnancy-related information** |  |  |  |  |  |  |  |  |  |  |  |
| **Gestational age (weeks), mean (SD)** | 15.66±2.86 | 14.81±3.20 | 0.290 |  | 15.60±2.86 | 15.52±3.31 | 0.926 |  | 15.58±2.87 | 15.63±3.04 | 0.939 |
| **Mode of pregnancy, n (%)** |  |  | 1.000 |  |  |  | 1.000 |  |  |  | 1.000 |
| Natural conception | 144 (98.6) | 14 (100.0) |  |  | 144 (98.6) | 14 (100.0) |  |  | 130 (98.5) | 28 (100.0) |  |
| Assisted reproduction | 2 (1.4) | 0 (0.00) |  |  | 2 (1.4) | 0 (0.0) |  |  | 2 (1.5) | 0 (0.0) |  |
| **Gravidity, n (%)** |  |  | 0.237 |  |  |  | 0.817 |  |  |  | 0.082 |
| 1 | 75 (51.4) | 4 (28.6) |  |  | 73 (50.0) | 6 (42.9) |  |  | 66 (50.0) | 13 (46.4) |  |
| 2 | 37 (25.3) | 5 (35.7) |  |  | 38 (26.0) | 4 (28.6) |  |  | 38 (28.8) | 4 (14.3) |  |
| ≥3 | 34 (23.3) | 5 (35.7) |  |  | 35 (24.0) | 4 (28.6) |  |  | 28 (21.2) | 11 (39.3) |  |
| **Parity, n (%)** |  |  | 0.773 |  |  |  | 1.000 |  |  |  | 0.870 |
| Primipara | 95 (65.1) | 10 (71.4) |  |  | 96 (65.8) | 9 (64.3) |  |  | 87 (65.9) | 18 (64.3) |  |
| Multipara | 51 (34.9) | 4 (28.6) |  |  | 50 (34.2) | 5 (35.7) |  |  | 45 (34.1) | 10 (35.7) |  |
| **Adverse obstetric** **history, n (%)** |  |  | **0.023** |  |  |  | 0.212 |  |  |  | 0.284 |
| No | 110 (75.3) | 6 (42.9) |  |  | 108 (74.0) | 8 (57.1) |  |  | 98 (74.2) | 18 (64.3) |  |
| Yes | 36 (24.7) | 8 (57.1) |  |  | 38 (26.0) | 6 (42.9) |  |  | 34 (25.8) | 10 (35.7) |  |
| **Pregnancy complications, n (%)** |  |  | 1.000 |  |  |  | 0.355 |  |  |  | 0.824 |
| Yes | 39 (26.7) | 4 (28.6) |  |  | 41 (28.1) | 2 (14.3) |  |  | 35 (26.5) | 8 (28.6) |  |
| No | 107 (73.3) | 10 (71.4) |  |  | 105 (71.9) | 12 (85.7) |  |  | 97 (73.5) | 20 (71.4) |  |
| **Planned pregnancy, n (%)** |  |  | 0.528 |  |  |  | 0.205 |  |  |  | 0.245 |
| Yes | 108 (74.0) | 9 (64.3) |  |  | 109 (74.7) | 8 (57.1) |  |  | 99 (75.0) | 18 (64.3) |  |
| No | 38 (26.0) | 5 (35.7) |  |  | 37 (25.3) | 6 (42.9) |  |  | 33 (25.0) | 10 (35.7) |  |
| **Primary outcome** |  |  |  |  |  |  |  |  |  |  | 0.653 |
| ISI, mean (SD) | 10.50*±*2.71 | 9.79±1.93 | 0.337 |  | 10.43±2.69 | 10.50±2.31 | 0.927 |  | 10.39*±*2.72 | 10.64±2.33 |  |
| **Secondary outcomes** |  |  |  |  |  |  |  |  |  |  |  |
| **Subjective sleep patterns** |  |  |  |  |  |  |  |  |  |  |  |
| SOL (minutes), median (P_25_, P_75_) | 27.86 (18.21,41.13) | 23.48 (11.19,45.75) | 0.438 |  | 27.86 (18.21,41.13) | 25.63 (11.96, 45.75) | 0.604 |  | 27.86 (18.72,40.80) | 25.63 (12.04,43.93) | 0.488 |
| WASO (minutes), median (P_25_, P_75_) | 11.43 (5.80,21.61) | 6.90 (3.84,12.63) | 0.057 |  | 11.43 (5.96,21.61) | 6.33 (3.84,9.18) | **0.013** |  | 11.43 (5.79,21.28) | 8.57 (5.00,16.88) | 0.162 |
| TST (hours), mean (SD) | 7.93*±*0.84 | 8.36±0.89 | 0.073 |  | 7.94±0.82 | 8.26±1.05 | 0.176 |  | 7.95*±*0.82 | 8.07±0.98 | 0.490 |
| SE (%), mean (SD) | 0.85*±*0.07 | 0.89±0.05 | 0.092 |  | 0.85*±*0.07 | 0.87±0.11 | 0.355 |  | 0.86*±*0.07 | 0.86*±*0.09 | 0.705 |
| **Subjective sleep quality** |  |  |  |  |  |  |  |  |  |  |  |
| PSQI, mean (SD) | 8.39*±*2.68 | 7.71±2.16 | 0.362 |  | 8.43*±*2.50 | 7.57±2.31 | 0.261 |  | 8.31*±*2.68 | 8.43*±*2.50 | 0.831 |
| **Daytime consequences** |  |  |  |  |  |  |  |  |  |  |  |
| FFS, mean (SD) | 10.26*±*3.93 | 9.64±3.46 | 0.567 |  | 10.24*±*3.86 | 9.93±4.23 | 0.775 |  | 10.26*±*3.85 | 10.00*±*4.11 | 0.751 |
| ESS, mean (SD) | 9.49*±*4.95 | 9.50±6.55 | 0.992 |  | 9.54*±*4.97 | 8.93±6.40 | 0.668 |  | 9.39*±*5.10 | 9.96*±*5.10 | 0.587 |
| **Mental health outcomes** |  |  |  |  |  |  |  |  |  |  |  |
| GAD-7, mean (SD) | 6.34*±*3.11 | 5.79±2.42 | 0.516 |  | 6.37*±*3.04 | 5.50±3.16 | 0.310 |  | 6.27*±*3.10 | 6.39*±*2.85 | 0.851 |
| EPDS, mean (SD) | 9.29*±*4.81 | 7.50±3.86 | 0.178 |  | 9.27*±*4.76 | 7.79±4.59 | 0.266 |  | 9.17*±*4.83 | 9.00*±*4.41 | 0.867 |
| **Hypothesized mediators** |  |  |  |  |  |  |  |  |  |  |  |
| DISRS, mean (SD) | 37.20*±*9.83 | 31.57±7.69 | **0.039** |  | 37.23*±*9.82 | 31.29±7.60 | **0.029** |  | 37.34*±*9.89 | 33.71*±*8.76 | 0.074 |
| APSQ, mean (SD) | 50.81*±*22.58 | 46.14±22.75 | 0.462 |  | 51.38*±*22.69 | 40.14±18.92 | 0.075 |  | 51.16*±*22.95 | 46.82*±*20.64 | 0.357 |
| PSAS, mean (SD) | 30.82*±*7.94 | 27.14±5.80 | 0.094 |  | 30.82*±*7.96 | 27.14±5.55 | 0.094 |  | 30.47*±*7.89 | 30.61*±*7.70 | 0.933 |
| SAMI-B, mean (SD) | 20.93*±*5.66 | 18.86±3.84 | 0.182 |  | 20.90*±*5.60 | 19.21±4.92 | 0.280 |  | 20.91*±*5.53 | 20.00*±*5.68 | 0.433 |
| SRBQ, mean (SD) | 38.74*±*18.69 | 32.57±19.42 | 0.241 |  | 38.50*±*18.62 | 35.07±20.80 | 0.516 |  | 38.52*±*18.83 | 36.68*±*18.77 | 0.638 |

Abbreviations: dMBI-PI, digital mindfulness-based intervention for prenatal insomnia symptoms; TAU, treatment as usual; BMI, body mass index; ISI, Insomnia Severity Index; SOL, sleep onset latency; WASO, wake after sleep onset; TST, total sleep time; SE, sleep efficiency; PSQI, Pittsburgh Sleep Quality Index; FFS, Flinders Fatigue Scale; ESS, Epworth Sleepiness Scale; GAD-7, Generalized Anxiety Disorder-7; EPDS, Edinburgh Postnatal Depression Scale; DISRS, Daytime Insomnia Symptom Response Scale; APSQ, Anxiety and Preoccupation about Sleep Questionnaire; PSAS, Pre-Sleep Arousal Scale; SAMI-B, Brief Version of the Sleep-Associated Monitoring Index; SRBQ, Sleep-Related Behaviors Questionnaire.
