# Supplementary material for: Effectiveness and Mechanisms of a Digital Mindfulness–Based Intervention for Subthreshold to Clinical Insomnia Symptoms in Pregnant Women: Randomized Controlled Trial
Source: J Med Internet Res. 2025 May 5;27:e68084. doi: 10.2196/68084 (PMC12089866; doi:10.2196/68084)
Supplement: Multimedia Appendix 5 [file jmir_v27i1e68084_app5.doc]

Correlations between primary outcome, secondary outcomes, and hypothesized mediators at baseline

|  | ISI | SOL | WASO | TST | SE | PSQI | FFS | ESS | GAD-7 | EPDS | DISRS | APSQ | PSAS | SAMI-B | SRBQ |
| --- | --- | --- | --- | --- | --- | --- | --- | --- | --- | --- | --- | --- | --- | --- | --- |
| ISI | 1 |  |  |  |  |  |  |  |  |  |  |  |  |  |  |
| SOL | 0.280^***^ | 1 |  |  |  |  |  |  |  |  |  |  |  |  |  |
| WASO | 0.147 | -0.046 | 1 |  |  |  |  |  |  |  |  |  |  |  |  |
| TST | -0.257^**^ | -0.248^**^ | -0.301^***^ | 1 |  |  |  |  |  |  |  |  |  |  |  |
| SE | -0.346^***^ | -0.697^***^ | -0.387^***^ | 0.588^***^ | 1 |  |  |  |  |  |  |  |  |  |  |
| PSQI | 0.595^***^ | 0.106 | 0.177^*^ | -0.234^**^ | -0.288^**^ | 1 |  |  |  |  |  |  |  |  |  |
| FFS | 0.272^***^ | -0.073 | 0.002 | -0.163^*^ | -0.008 | 0.283^***^ | 1 |  |  |  |  |  |  |  |  |
| ESS | 0.095 | 0.087 | 0.045 | -0.131 | -0.128 | 0.075 | 0.194^*^ | 1 |  |  |  |  |  |  |  |
| GAD-7 | 0.344^***^ | -0.125 | 0.012 | -0.191^*^ | 0.000 | 0.343^***^ | 0.494^***^ | 0.349^***^ | 1 |  |  |  |  |  |  |
| EPDS | 0.158^*^ | 0.054 | -0.019 | -0.178^*^ | -0.135 | 0.208^**^ | 0.448^***^ | 0.433^***^ | 0.554^***^ | 1 |  |  |  |  |  |
| DISRS | 0.394^***^ | 0.094 | 0.028 | -0.124 | -0.154 | 0.403^***^ | 0.432^***^ | 0.299^***^ | 0.577^***^ | 0.490^***^ | 1 |  |  |  |  |
| APSQ | 0.355^***^ | -0.020 | 0.209^**^ | -0.196^*^ | -0.172^*^ | 0.437^***^ | 0.257^**^ | 0.285^***^ | 0.298^***^ | 0.386^***^ | 0.382^***^ | 1 |  |  |  |
| PSAS | 0.399^***^ | 0.141 | 0.081 | -0.127 | -0.232^**^ | 0.393^***^ | 0.444^***^ | 0.321^***^ | 0.566^***^ | 0.486^***^ | 0.694^***^ | 0.478^***^ | 1 |  |  |
| SAMI-B | 0.304^***^ | 0.041 | 0.134 | -0.101 | -0.132 | 0.300^***^ | 0.437^***^ | 0.470^***^ | 0.429^***^ | 0.515^***^ | 0.401^***^ | 0.486^***^ | 0.518^***^ | 1 |  |
| SRBQ | 0.244^**^ | 0.158^*^ | 0.113 | -0.168^*^ | -0.235^**^ | 0.204^**^ | 0.395^***^ | 0.473^***^ | 0.418^***^ | 0.513^***^ | 0.494^***^ | 0.494^***^ | 0.539^***^ | 0.630^***^ | 1 |

Note: ^*^ represents *p* <0.05; ^**^ represents *p* <0.01; ^***^ represents *p* <0.001. ISI, Insomnia Severity Index; SOL, sleep onset latency; WASO, wake after sleep onset; TST, total sleep time; SE, sleep efficiency; PSQI, Pittsburgh Sleep Quality Index; FFS, Flinders Fatigue Scale; ESS, Epworth Sleepiness Scale; GAD-7, Generalized Anxiety Disorder-7; EPDS, Edinburgh Postnatal Depression Scale; DISRS, Daytime Insomnia Symptom Response Scale; APSQ, Anxiety and Preoccupation about Sleep Questionnaire; PSAS, Pre-Sleep Arousal Scale; SAMI-B, Brief Version of the Sleep-Associated Monitoring Index; SRBQ, Sleep-Related Behaviors Questionnaire.
