# Supplementary material for: Effectiveness and Mechanisms of a Digital Mindfulness–Based Intervention for Subthreshold to Clinical Insomnia Symptoms in Pregnant Women: Randomized Controlled Trial
Source: J Med Internet Res. 2025 May 5;27:e68084. doi: 10.2196/68084 (PMC12089866; doi:10.2196/68084)
Supplement: Multimedia Appendix 6 [file jmir_v27i1e68084_app6.docx]

Mixed-effects analysis of changes in primary and secondary outcomes from baseline to follow-up

|  | Mean (SE) ^a^/ n (%) ^b^ | | | |  | Change from time 1 to time 2 | | | | |  | Change from time 1 to time 3 | | | | |  | Change from time 1 to time 4 | | | | |
| --- | --- | --- | --- | --- | --- | --- | --- | --- | --- | --- | --- | --- | --- | --- | --- | --- | --- | --- | --- | --- | --- | --- |
|  |  |  |  |  |  | within-group ^c^ | | between-group difference | | |  | within-group ^c^ | | between-group difference | | |  | within-group ^c^ | | between-group difference | | |
| Measure | Time 1 (baseline) | Time 2 (post-intervention) | Time 3 (two months after post-intervention) | Time 4 (42 days postpartum) |  | change in score | *P* value | *β* (95% *CI*) ^d^/  *OR* (95% *CI*) ^e^ | *P* value | Adjusted *P* value ^f^ |  | change in score | *P* value | *β* (95% *CI*) ^d^/  *OR* (95% *CI*) ^e^ | *P* value | Adjusted *P* value ^f^ |  | change in score | *P* value | *β* (95% *CI*) ^d^/  *OR* (95% *CI*) ^e^ | *P* value | Adjusted *P* value ^f^ |
| **Primary outcome: ISI scores ^a^** | | | | | | | | | | | | | | | | | | | | | | |
| dMBI-PI+TAU | 10.84 (0.41) | 5.45 (0.43) | 6.39 (0.43) | 7.83 (0.45) |  | -5.38 | <0.001 | -2.02 (-3.42 to -0.62) | 0.005 | NA |  | -4.45 | <0.001 | -2.02 (-3.42 to -0.61) | 0.005 | NA |  | -3.01 | <0.001 | -1.12 (-2.57 to 0.33) | 0.131 | NA |
| TAU | 10.04 (0.41) | 6.67 (0.43) | 7.61 (0.43) | 8.15 (0.45) |  | -3.37 | <0.001 |  |  |  |  | -2.43 | <0.001 |  |  |  |  | -1.89 | <0.001 |  |  |  |
| **Secondary outcome: rate of remission from insomnia symptoms ^b^** | | | | | | | | | | | | | | | | | | | | | | |
| dMBI-PI+TAU | NA | 54 (67.5) | 50 (62.5) | 32 (40.0) |  | NA | NA | 1.68 (0.87 to 3.22) | 0.122 | 0.304 |  | NA | NA | 2.31 (1.21 to 4.39) | 0.011 | 0.039 |  | NA | NA | 1.18 (0.61 to 2.26) | 0.624 | 0.944 |
| TAU | NA | 45 (56.3) | 34 (42.5) | 31 (38.8) |  | NA | NA |  |  |  |  | NA | NA |  |  |  |  | NA | NA |  |  |  |
| **Secondary outcome: rate of achieving reliable change in ISI scores ^b^** | | | | | | | | | | | | | | | | | | | | | | |
| dMBI-PI+TAU | NA | 53 (66.3) | 50 (62.5) | 38 (47.5) |  | NA | NA | 1.59 (0.80 to 3.13) | 0.185 | 0.339 |  | NA | NA | 2.64 (1.35 to 5.16) | 0.004 | 0.028 |  | NA | NA | 1.48 (0.78 to 2.82) | 0.230 | 0.537 |
| TAU | NA | 41 (51.3) | 29 (36.3) | 29 (36.3) |  | NA | NA |  |  |  |  | NA | NA |  |  |  |  | NA | NA |  |  |  |
| **Secondary outcome: SOL (mins) ^a^** | | | | | | | | | | | | | | | | | | | | | | |
| dMBI-PI+TAU | 34.8 (3.42) | 29.9 (3.60) | NA | NA |  | -4.85 | 0.127 | -3.52 (-12.12 to 5.08) | 0.424 | 0.465 |  | NA | NA | NA | NA | NA |  | NA | NA | NA | NA | NA |
| TAU | 35.1 (3.42) | 33.8 (3.55) | NA | NA |  | -1.33 | 0.669 |  |  |  |  | NA | NA |  |  |  |  | NA | NA |  |  |  |
| **Secondary outcome: WASO (mins) ^a^** | | | | | | | | | | | | | | | | | | | | | | |
| dMBI-PI+TAU | 16.06 (1.44) | 9.31 (1.53) | NA | NA |  | -6.75 | <0.001 | -3.31 (-7.66 to 1.04) | 0.138 | 0.304 |  | NA | NA | NA | NA | NA |  | NA | NA | NA | NA | NA |
| TAU | 15.52 (1.44) | 12.08 (1.51) | NA | NA |  | -3.45 | 0.030 |  |  |  |  | NA | NA |  |  |  |  | NA | NA |  |  |  |
| **Secondary outcome: TST (hours) ^a^** | | | | | | | | | | | | | | | | | | | | | | |
| dMBI-PI+TAU | 7.96 (0.10) | 8.19 (0.10) | NA | NA |  | 0.23 | 0.012 | 0.09 (-0.15 to 0.34) | 0.465 | 0.465 |  | NA | NA | NA | NA | NA |  | NA | NA | NA | NA | NA |
| TAU | 7.98 (0.10) | 8.12 (0.10) | NA | NA |  | 0.14 | 0.120 |  |  |  |  | NA | NA |  |  |  |  | NA | NA |  |  |  |
| **Secondary outcome: SE (%) ^a^** | | | | | | | | | | | | | | | | | | | | | | |
| dMBI-PI+TAU | 0.85 (0.01) | 0.89 (0.01) | NA | NA |  | 0.03 | <0.001 | 0.02 (0.00 to 0.04) | 0.044 | 0.220 |  | NA | NA | NA | NA | NA |  | NA | NA | NA | NA | NA |
| TAU | 0.86 (0.01) | 0.87 (0.01) | NA | NA |  | 0.01 | 0.085 |  |  |  |  | NA | NA |  |  |  |  | NA | NA |  |  |  |
| **Secondary outcome: PSQI ^a^** | | | | | | | | | | | | | | | | | | | | | | |
| dMBI-PI+TAU | 8.57 (0.33) | 5.40 (0.35) | 6.31 (0.34) | 8.75 (0.36) |  | -3.17 | <0.001 | -1.47 (-2.57 to -0.37) | 0.009 | 0.099 |  | -2.27 | <0.001 | -1.30 (-2.39 to -0.21) | 0.020 | 0.047 |  | 0.18 | 0.665 | -1.12 (-2.25 to 0.01) | 0.053 | 0.252 |
| TAU | 8.09 (0.33) | 6.39 (0.34) | 7.12 (0.34) | 9.38 (0.36) |  | -1.70 | <0.001 |  |  |  |  | -0.97 | 0.015 |  |  |  |  | 1.30 | 0.002 |  |  |  |
| **Secondary outcome: FFS ^a^** | | | | | | | | | | | | | | | | | | | | | | |
| dMBI-PI+TAU | 9.85 (0.46) | 7.12 (0.48) | 8.25 (0.48) | 10.39 (0.49) |  | -2.73 | <0.001 | -0.87 (-2.28 to 0.53) | 0.225 | 0.354 |  | -1.60 | 0.002 | -0.58 (-1.99 to 0.82) | 0.416 | 0.485 |  | 0.54 | 0.302 | -0.05 (-1.50 to 1.40) | 0.944 | 0.944 |
| TAU | 10.57 (0.46) | 8.72 (0.48) | 9.56 (0.48) | 11.17 (0.50) |  | -1.85 | <0.001 |  |  |  |  | -1.01 | 0.048 |  |  |  |  | 0.60 | 0.262 |  |  |  |
| **Secondary outcome: ESS ^a^** | | | | | | | | | | | | | | | | | | | | | | |
| dMBI-PI+TAU | 9.29 (0.61) | 7.85 (0.63) | 8.41 (0.62) | 10.12 (0.64) |  | -1.44 | 0.013 | -0.79 (-2.36 to 0.78) | 0.323 | 0.395 |  | -0.88 | 0.124 | -0.88 (-2.45 to 0.69) | 0.272 | 0.381 |  | 0.83 | 0.158 | -0.152 (-1.79 to 1.48) | 0.855 | 0.944 |
| TAU | 9.69 (0.61) | 9.04 (0.62) | 9.69 (0.63) | 10.67 (0.65) |  | -0.65 | 0.253 |  |  |  |  | 0.00 | 0.997 |  |  |  |  | 0.99 | 0.100 |  |  |  |
| **Secondary outcome: GAD-7 ^a^** | | | | | | | | | | | | | | | | | | | | | | |
| dMBI-PI+TAU | 6.30 (0.36) | 4.16 (0.37) | 4.73 (0.37) | 4.97 (0.38) |  | -2.14 | <0.001 | -0.96 (-1.97 to 0.04) | 0.060 | 0.220 |  | -1.57 | <0.001 | -0.86 (-1.87 to 0.14) | 0.093 | 0.163 |  | -1.33 | <0.001 | -0.95 (-1.99 to 0.08) | 0.072 | 0.252 |
| TAU | 6.29 (0.36) | 5.11 (0.37) | 5.58 (0.37) | 5.91 (0.38) |  | -1.18 | 0.001 |  |  |  |  | -0.71 | 0.052 |  |  |  |  | -0.38 | 0.322 |  |  |  |
| **Secondary outcome: EPDS ^a^** | | | | | | | | | | | | | | | | | | | | | | |
| dMBI-PI+TAU | 8.57 (0.57) | 6.57 (0.60) | 6.90 (0.59) | 7.57 (0.61) |  | -2.00 | <0.001 | -0.89 (-2.49 to 0.71) | 0.277 | 0.381 |  | -1.68 | 0.004 | -0.50 (-2.10 to 1.09) | 0.537 | 0.537 |  | -1.01 | 0.093 | -0.06 (-1.71 to 1.59) | 0.942 | 0.944 |
| TAU | 9.70 (0.57) | 8.59 (0.59) | 8.53 (0.59) | 8.76 (0.61) |  | -1.11 | 0.056 |  |  |  |  | -1.17 | 0.044 |  |  |  |  | -0.94 | 0.118 |  |  |  |

Abbreviations: dMBI-PI, digital mindfulness-based intervention for prenatal insomnia symptoms; TAU, treatment as usual; NA, not applicable; ISI, Insomnia Severity Index; SOL, sleep onset latency; WASO, wake after sleep onset; TST, total sleep time; SE, sleep efficiency; PSQI, Pittsburgh Sleep Quality Index; FFS, Flinders Fatigue Scale; ESS, Epworth Sleepiness Scale; GAD-7, Generalized Anxiety Disorder-7; EPDS, Edinburgh Postnatal Depression Scale. ^a^ Mean (SE) presented is least squares mean (standard error) from mixed-effects linear regression model. ^b^ n (%) presented is the number (proportion) of remission and reliable change with dropouts defined as no remission from insomnia symptoms and no reliable change in ISI score. ^c^ Estimated within-group change and *P* value from mixed-effects linear regression model. ^d^ Estimated between-group differences in changes in ISI scores over time (group × time interactions) from mixed-effects linear regression model. ^e^ Estimated between-group differences in the likelihood of remission or achieving reliable change from logistic regression model (ISI score at baseline was included as a covariate), with dropouts defined as no remission and no reliable change, respectively. ^f^ *P* value after controlling for multiple testing due to multiple secondary outcomes using the Benjamini-Hochberg (BH) false discovery rate correction.
