# Supplementary material for: Effectiveness and Mechanisms of a Digital Mindfulness–Based Intervention for Subthreshold to Clinical Insomnia Symptoms in Pregnant Women: Randomized Controlled Trial
Source: J Med Internet Res. 2025 May 5;27:e68084. doi: 10.2196/68084 (PMC12089866; doi:10.2196/68084)
Supplement: Multimedia Appendix 7 [file jmir_v27i1e68084_app7.doc]

Mixed-effects analysis of change in primary and secondary outcomes from baseline to follow-up using imputed datasets

|  | Mean (SE) ^a^/ % (SE) ^b^ | | | |  | Change from time 1 to time 2 | | | | |  | Change from time 1 to time 3 | | | | |  | Change from time 1 to time 4 | | | | |
| --- | --- | --- | --- | --- | --- | --- | --- | --- | --- | --- | --- | --- | --- | --- | --- | --- | --- | --- | --- | --- | --- | --- |
|  |  |  |  |  |  | within-group ^c^ | | between-group difference | | |  | within-group ^c^ | | between-group difference | | |  | within-group ^c^ | | between-group difference | | |
| Measure | Time 1 (baseline) | Time 2 (post-intervention) | Time 3 (two months after post-intervention) | Time 4 (42 days postpartum) |  | change in score | *P* value | *β* (95% *CI*) ^d^/  *OR* (95% *CI*) ^e^ | *P* value | Adjusted *P* value ^f^ |  | change in score | *P* value | *β* (95% *CI*) ^d^/  *OR* (95% *CI*) ^e^ | *P* value | Adjusted *P* value ^f^ |  | change in score | *P* value | *β* (95% *CI*) ^d^/  *OR* (95% *CI*) ^e^ | *P* value | Adjusted *P* value ^f^ |
| **Primary outcome: ISI scores ^a^** | | | | | | | | | | | | | | | | | | | | | | |
| dMBI-PI+TAU | 10.97 (0.47) | 5.57 (0.47) | 6.38 (0.47) | 7.89 (0.47) |  | -5.40 | <0.001 | -2.22 (-3.76 to -0.67) | 0.005 | NA |  | -4.59 | <0.001 | -2.28(-3.82 to -0.73) | 0.0064 | NA |  | -3.08 | <0.001 | -1.31 (-2.85 to 0.23) | 0.097 | NA |
| TAU | 9.89 (0.47) | 6.71 (0.47) | 7.58 (0.47) | 8.12 (0.47) |  | -3.19 | <0.001 |  |  |  |  | -2.31 | <0.001 |  |  |  |  | -1.77 | 0.009 |  |  |  |
| **Secondary outcome: rate of remission from insomnia symptoms ^b^** | | | | | | | | | | | | | | | | | | | | | | |
| dMBI-PI+TAU | NA | 75.1 (0.05) | 68.4 (0.05) | 47.6 (0.05) |  | NA | NA | 1.98 (0.94 to 4.17) | 0.071 | 0.242 |  | NA | NA | 2.50 (1.25 to 4.99) | 0.010 | 0.035 |  | NA | NA | 1.06 (0.51 to 2.22) | 0.870 | 0.996 |
| TAU | NA | 61.9 (0.06) | 47.1 (0.06) | 48.2 (0.06) |  | NA | NA |  |  |  |  | NA | NA |  |  |  |  | NA | NA |  |  |  |
| **Secondary outcome: rate of achieving** **reliable change in ISI scores ^b^** | | | | | | | | | | | | | | | | | | | | | | |
| dMBI-PI+TAU | NA | 73.1 (0.05) | 68.9 (0.05) | 56.1 (0.05) |  | NA | NA | 1.79 (0.84 to 3.83) | 0.129 | 0.284 |  | NA | NA | 2.95 (1.43 to 6.10) | 0.004 | 0.028 |  | NA | NA | 1.30 (0.64 to 2.65) | 0.463 | 0.996 |
| TAU | NA | 56.4 (0.06) | 40.5 (0.06) | 46.6 (0.06) |  | NA | NA |  |  |  |  | NA | NA |  |  |  |  | NA | NA |  |  |  |
| **Secondary outcome: SOL (mins)** **^a^** | | | | | | | | | | | | | | | | | | | | | | |
| dMBI-PI+TAU | 34.8 (3.42) | 31.2 (3.66) | NA | NA |  | -3.57 | 0.280 | -1.90 (-10.98 to7.19) | 0.681 | 0.681 |  | NA | NA | NA | NA |  |  | NA | NA | NA | NA | NA |
| TAU | 35.1 (3.42) | 33.4 (3.53) | NA | NA |  | -1.67 | 0.596 |  |  |  |  | NA | NA |  |  |  |  | NA | NA |  |  |  |
| **Secondary outcome: WASO (mins) ^a^** | | | | | | | | | | | | | | | | | | | | | | |
| dMBI-PI+TAU | 16.10 (1.41) | 9.70 (1.43) | NA | NA |  | -6.37 | <0.001 | -2.98 (-7.16 to -1.20) | 0.162 | 0.297 |  | NA | NA | NA | NA |  |  | NA | NA | NA | NA | NA |
| TAU | 15.50 (1.41) | 12.10 (1.43) | NA | NA |  | -3.39 | 0.027 |  |  |  |  | NA | NA |  |  |  |  | NA | NA |  |  |  |
| **Secondary outcome: TST (hours) ^a^** | | | | | | | | | | | | | | | | | | | | | | |
| dMBI-PI+TAU | 7.96 (0.10) | 8.16 (0.10) | NA | NA |  | 0.20 | 0.038 | 0.06 (-0.21 to 0.32) | 0.674 | 0.681 |  | NA | NA | NA | NA |  |  | NA | NA | NA | NA | NA |
| TAU | 7.98 (0.10) | 8.12 (0.10) | NA | NA |  | 0.14 | 0.134 |  |  |  |  | NA | NA |  |  |  |  | NA | NA |  |  |  |
| **Secondary outcome: SE (%) ^a^** | | | | | | | | | | | | | | | | | | | | | | |
| dMBI-PI+TAU | 0.85 (0.01) | 0.89 (0.01) | NA | NA |  | 0.03 | <0.001 | 0.02 (0.00 to 0.04) | 0.088 | 0.242 |  | NA | NA | NA | NA |  |  | NA | NA | NA | NA | NA |
| TAU | 0.86 (0.01) | 0.87 (0.01) | NA | NA |  | 0.01 | 0.056 |  |  |  |  | NA | NA |  |  |  |  | NA | NA |  |  |  |
| **Secondary outcome: PSQI ^a^** | | | | | | | | | | | | | | | | | | | | | | |
| dMBI-PI+TAU | 8.57 (0.33) | 5.45 (0.35) | 6.25 (0.34) | 8.68 (0.35) |  | -3.12 | <0.001 | -1.38 (-2.49 to -0.28) | 0.014 | 0.154 |  | -2.32 | <0.001 | -1.28 (-2.38 to -0.17) | 0.024 | 0.056 |  | 0.11 | 0.791 | -1.20 (-2.31 to -0.08) | 0.036 | 0.252 |
| TAU | 8.09 (0.33) | 6.35 (0.34) | 7.04 (0.34) | 9.39 (0.35) |  | -1.74 | <0.001 |  |  |  |  | -1.05 | 0.009 |  |  |  |  | 1.30 | 0.002 |  |  |  |
| **Secondary outcome: FFS ^a^** | | | | | | | | | | | | | | | | | | | | | | |
| dMBI-PI+TAU | 9.85 (0.47) | 7.05 (0.48) | 8.26 (0.48) | 10.49 (0.53) |  | -2.80 | <0.001 | -0.94 (-2.37 to 0.50) | 0.199 | 0.313 |  | -1.59 | 0.003 | -0.49 (1.95 to 0.97) | 0.511 | 0.545 |  | 0.64 | 0.261 | 0.00 (-1.67 to 1.68) | 0.996 | 0.996 |
| TAU | 10.57 (0.47) | 8.71 (0.48) | 9.48 (0.49) | 11.21 (0.52) |  | -1.86 | <0.001 |  |  |  |  | -1.10 | 0.039 |  |  |  |  | 0.63 | 0.256 |  |  |  |
| **Secondary outcome: ESS ^a^** | | | | | | | | | | | | | | | | | | | | | | |
| dMBI-PI+TAU | 9.29 (0.61) | 7.82 (0.64) | 8.35 (0.63) | 9.98 (0.70) |  | -1.47 | 0.017 | -0.78 (-2.47 to 0.90) | 0.361 | 0.441 |  | -0.94 | 0.126 | -0.86 (-2.53 to 0.81) | 0.312 | 0.437 |  | 0.69 | 0.305 | -0.15 (-2.22 to 1.93) | 0.886 | 0.996 |
| TAU | 9.69 (0.61) | 9.00 (0.62) | 9.61 (0.63) | 10.53 (0.75) |  | -0.69 | 0.251 |  |  |  |  | -0.08 | 0.899 |  |  |  |  | 0.84 | 0.252 |  |  |  |
| **Secondary outcome: GAD-7 ^a^** | | | | | | | | | | | | | | | | | | | | | | |
| dMBI-PI+TAU | 6.30 (0.36) | 4.13 (0.39) | 4.76 (0.37) | 5.09 (0.39) |  | -2.17 | <0.001 | -0.94 (-2.00 to 0.13) | 0.085 | 0.242 |  | -1.54 | <0.001 | -0.78 (-1.82 to 0.27) | 0.144 | 0.252 |  | -1.21 | 0.003 | -0.80 (-1.92 to 0.33) | 0.165 | 0.578 |
| TAU | 6.29 (0.36) | 5.05 (0.37) | 5.53 (0.37) | 5.87 (0.40) |  | -1.23 | 0.001 |  |  |  |  | -0.76 | 0.049 |  |  |  |  | -0.42 | 0.305 |  |  |  |
| **Secondary outcome: EPDS ^a^** | | | | | | | | | | | | | | | | | | | | | | |
| dMBI-PI+TAU | 8.57 (0.57) | 6.54 (0.58) | 6.91 (0.58) | 7.69 (0.60) |  | -2.03 | <0.001 | -0.92 (-2.54 to 0.70) | 0.264 | 0.363 |  | -1.67 | 0.005 | -0.49 (-2.09 to 1.11) | 0.545 | 0.545 |  | -0.89 | 0.144 | 0.13 (-1.59 to 1.84) | 0.885 | 0.996 |
| TAU | 9.70 (0.57) | 8.59 (0.58) | 8.53 (0.58) | 8.69 (0.60) |  | -1.11 | 0.057 |  |  |  |  | -1.18 | 0.045 |  |  |  |  | -1.01 | 0.094 |  |  |  |

Abbreviations: dMBI-PI, digital mindfulness-based intervention for prenatal insomnia symptoms; TAU, treatment as usual; NA, not applicable; ISI, Insomnia Severity Index; SOL, sleep onset latency; WASO, wake after sleep onset; TST, total sleep time; SE, sleep efficiency; PSQI, Pittsburgh Sleep Quality Index; FFS, Flinders Fatigue Scale; ESS, Epworth Sleepiness Scale; GAD-7, Generalized Anxiety Disorder-7; EPDS, Edinburgh Postnatal Depression Scale. ^a^ Mean (SE) presented is least squares mean (standard error) from mixed-effects linear regression model. ^b^ n (%) presented is the pooled proportion and standard error of remission from insomnia symptoms and reliable change in ISI score according to Wald acorss multiply imputed datasets. ^c^ Estimated within-group change and *P* value from mixed-effects linear regression model. ^d^ Estimated between-group differences in changes in ISI scores over time (group × time interactions) from mixed-effects linear regression model. ^e^ Estimated between-group differences in the likelihood of remission or achieving reliable change from logistic regression model (ISI score at baseline was included as a covariate). ^f^ *P* value after controlling for multiple testing due to multiple secondary outcomes using the Benjamini-Hochberg (BH) false discovery rate correction.
