# Supplementary material for: Effectiveness and Mechanisms of a Digital Mindfulness–Based Intervention for Subthreshold to Clinical Insomnia Symptoms in Pregnant Women: Randomized Controlled Trial
Source: J Med Internet Res. 2025 May 5;27:e68084. doi: 10.2196/68084 (PMC12089866; doi:10.2196/68084)
Supplement: Multimedia Appendix 8 [file jmir_v27i1e68084_app8.doc]

Mixed-effects analysis of change in primary and secondary outcomes from baseline to follow-up (complete cases analysis)

|  | Mean (SE) ^a^/ n (%) ^b^ | | | |  | Change from time 1 to time 2 | | | | |  | Change from time 1 to time 3 | | | | |  | Change from time 1 to time 4 | | | | |
| --- | --- | --- | --- | --- | --- | --- | --- | --- | --- | --- | --- | --- | --- | --- | --- | --- | --- | --- | --- | --- | --- | --- |
|  |  |  |  |  |  | within-group ^c^ | | between-group difference | | |  | within-group ^c^ | | between-group difference | | |  | within-group ^c^ | | between-group difference | | |
| Measure | Time 1 (baseline) | Time 2 (post-intervention) | Time 3 (two months after post-intervention) | Time 4 (42 days postpartum) |  | change in score | *P* value | *β* (95% *CI*) ^d^/  *OR* (95% *CI*) ^e^ | *P* value | Adjusted *P* value ^f^ |  | change in score | *P* value | *β* (95% *CI*) ^d^/  *OR* (95% *CI*) ^e^ | *P* value | Adjusted *P* value ^f^ |  | change in score | *P* value | *β* (95% *CI*) ^d^/  *OR* (95% *CI*) ^e^ | *P* value | Adjusted *P* value ^f^ |
| **Primary outcome: ISI scores ^a^** | | | | | | | | | | | | | | | | | | | | | | |
| dMBI-PI+TAU | 10.84 (0.42) | 5.49 (0.42) | 6.39 (0.44) | 7.95 (0.49) |  | -5.35 | <0.001 | -1.89 (-3.31 to -0.47) | 0.009 | NA |  | -4.45 | <0.001 | -2.00 (-3.43 to -0.57) | 0.006 | NA |  | -2.89 | <0.001 | -0.84 (-2.43 to 0.75) | 0.299 | NA |
| TAU | 10.04 (0.42) | 6.57 (0.43) | 7.59 (0.44) | 7.99 (0.46) |  | -3.46 | <0.001 |  |  |  |  | -2.45 | <0.001 |  |  |  |  | -2.05 | <0.001 |  |  |  |
| **Secondary outcome: rate of remission from insomnia symptoms ^b^** | | | | | | | | | | | | | | | | | | | | | | |
| dMBI-PI+TAU | NA | 48 (73.80) | 44 (67.70) | 31 (47.70) |  | NA | NA | 2.02 (0.93 to 4.39) | 0.078 | 0.215 |  | NA | NA | 2.39 (1.15 to 4.96) | 0.019 | 0.067 |  | NA | NA | 1.16 (0.57 to 2.38) | 0.678 | 1.000 |
| TAU | NA | 40 (61.50) | 31 (47.70) | 31 (47.70) |  | NA | NA |  |  |  |  | NA | NA |  |  |  |  | NA | NA |  |  |  |
| **Secondary outcome: rate of achieving reliable change in ISI scores ^b^** | | | | | | | | | | | | | | | | | | | | | | |
| dMBI-PI+TAU | NA | 48 (73.80) | 46 (70.80) | 37 (56.90) |  | NA | NA | 2.11 (0.97 to 4.61) | 0.060 | 0.220 |  | NA | NA | 3.34 (1.55 to 7.18) | 0.002 | 0.014 |  | NA | NA | 1.45 (0.71 to 2.96) | 0.305 | 0.712 |
| TAU | NA | 34 (52.30) | 25 (38.50) | 29 (44.60) |  | NA | NA |  |  |  |  | NA | NA |  |  |  |  | NA | NA |  |  |  |
| **Secondary outcome: SOL (mins) ^a^** | | | | | | | | | | | | | | | | | | | | | | |
| dMBI-PI+TAU | 31.60 (3.39) | 29.40 (3.44) | NA | NA |  | -2.21 | 0.459 | -0.54 (-8.68 to7.60) | 0.896 | 0.896 |  | NA | NA | NA | NA |  |  | NA | NA | NA | NA | NA |
| TAU | 36.50 (3.39) | 34.90 (3.43) | NA | NA |  | -1.67 | 0.573 |  |  |  |  | NA | NA |  |  |  |  | NA | NA |  |  |  |
| **Secondary outcome: WASO (mins) ^a^** | | | | | | | | | | | | | | | | | | | | | | |
| dMBI-PI+TAU | 17.00 (1.60) | 9.70 (1.63) | NA | NA |  | -7.34 | <0.001 | -4.06 (-8.90 to 0.78) | 0.103 | 0.227 |  | NA | NA | NA | NA |  |  | NA | NA | NA | NA | NA |
| TAU | 15.60 (1.60) | 12.30 (1.62) | NA | NA |  | -3.28 | 0.064 |  |  |  |  | NA | NA |  |  |  |  | NA | NA |  |  |  |
| **Secondary outcome: TST (hours) ^a^** | | | | | | | | | | | | | | | | | | | | | | |
| dMBI-PI+TAU | 7.94 (0.11) | 8.15 (0.11) | NA | NA |  | 0.21 | 0.024 | 0.06 (-0.21 to 0.32) | 0.674 | 0.741 |  | NA | NA | NA | NA |  |  | NA | NA | NA | NA | NA |
| TAU | 7.96 (0.11) | 8.17 (0.11) | NA | NA |  | 0.21 | 0.025 |  |  |  |  | NA | NA |  |  |  |  | NA | NA |  |  |  |
| **Secondary outcome: SE (%) ^a^** | | | | | | | | | | | | | | | | | | | | | | |
| dMBI-PI+TAU | 0.86 (0.01) | 0.89 (0.01) | NA | NA |  | 0.03 | <0.001 | 0.02 (0.00 to 0.04) | 0.129 | 0.237 |  | NA | NA | NA | NA |  |  | NA | NA | NA | NA | NA |
| TAU | 0.86 (0.01) | 0.87 (0.01) | NA | NA |  | 0.01 | 0.067 |  |  |  |  | NA | NA |  |  |  |  | NA | NA |  |  |  |
| **Secondary outcome: PSQI ^a^** | | | | | | | | | | | | | | | | | | | | | | |
| dMBI-PI+TAU | 8.60 (0.37) | 5.36 (0.38) | 6.37 (0.37) | 8.81 (0.37) |  | -3.24 | <0.001 | -1.53 (-2.72 to -0.33) | 0.013 | 0.143 |  | -2.23 | <0.001 | -1.28 (-2.46 to -0.10) | 0.024 | 0.056 |  | 0.21 | 0.965 | -1.13 (-2.32 to 0.06) | 0.064 | 0.224 |
| TAU | 8.00 (0.37) | 6.29 (0.37) | 7.05 (0.37) | 9.33 (0.37) |  | -1.71 | <0.001 |  |  |  |  | -0.95 | 0.124 |  |  |  |  | 1.33 | 0.012 |  |  |  |
| **Secondary outcome: FFS ^a^** | | | | | | | | | | | | | | | | | | | | | | |
| dMBI-PI+TAU | 9.85 (0.52) | 6.97 (0.52) | 8.23 (0.52) | 10.35 (0.52) |  | -2.88 | <0.001 | -1.02 (-2.55 to 0.52) | 0.195 | 0.306 |  | -1.62 | 0.021 | -0.66 (2.55 to 0.52) | 0.399 | 0.466 |  | 0.51 | 0.800 | -0.08 (-1.61 to 1.46) | 0.922 | 0.922 |
| TAU | 10.65 (0.52) | 8.78 (0.52) | 9.69 (0.52) | 11.23 (0.52) |  | -1.86 | 0.005 |  |  |  |  | -0.95 | 0.320 |  |  |  |  | 0.59 | 0.721 |  |  |  |
| **Secondary outcome: ESS ^a^** | | | | | | | | | | | | | | | | | | | | | | |
| dMBI-PI+TAU | 8.97 (0.68) | 7.59 (0.68) | 8.17 (0.68) | 9.83 (0.68) |  | -1.38 | 0.111 | -0.94 (-2.62 to 0.75) | 0.276 | 0.380 |  | -0.80 | 0.556 | -1.01 (-2.69 to 0.67) | 0.241 | 0.337 |  | 0.87 | 0.493 | -0.24 (-1.94 to 1.45) | 0.777 | 1.000 |
| TAU | 9.62 (0.68) | 9.17 (0.68) | 9.82 (0.68) | 10.72 (0.68) |  | -0.45 | 0.885 |  |  |  |  | 0.21 | 0.987 |  |  |  |  | 1.11 | 0.275 |  |  |  |
| **Secondary outcome: GAD-7 ^a^** | | | | | | | | | | | | | | | | | | | | | | |
| dMBI-PI+TAU | 6.34 (0.40) | 4.03 (0.40) | 4.69 (0.40) | 4.88 (0.40) |  | -2.31 | <0.001 | -1.32 (-2.40 to -0.25) | 0.016 | 0.088 |  | -1.65 | <0.001 | -1.20 (-2.27 to -0.12) | 0.029 | 0.051 |  | -1.46 | 0.001 | -1.23 (-2.30 to -0.16) | 0.025 | 0.175 |
| TAU | 6.17 (0.40) | 5.18 (0.40) | 5.72 (0.40) | 5.94 (0.40) |  | -0.99 | 0.058 |  |  |  |  | -0.45 | 0.663 |  |  |  |  | -0.23 | 0.935 |  |  |  |
| **Secondary outcome: EPDS ^a^** | | | | | | | | | | | | | | | | | | | | | | |
| dMBI-PI+TAU | 8.54 (0.64) | 6.48 (0.65) | 6.85 (0.64) | 7.42 (0.64) |  | -2.06 | 0.007 | -0.89 (-2.62 to 0.85) | 0.317 | 0.387 |  | -1.69 | 0.037 | -0.69 (-2.42 to 1.03) | 0.432 | 0.432 |  | -1.12 | 0.280 | -0.20 (-1.93 to 1.53) | 0.821 | 0.958 |
| TAU | 9.74 (0.64) | 8.57 (0.65) | 8.74 (0.64) | 8.82 (0.64) |  | -1.17 | 0.249 |  |  |  |  | -1.00 | 0.384 |  |  |  |  | -0.92 | 0.457 |  |  |  |

Abbreviations: dMBI-PI, digital mindfulness-based intervention for prenatal insomnia symptoms; TAU, treatment as usual; NA, not applicable; ISI, Insomnia Severity Index; SOL, sleep onset latency; WASO, wake after sleep onset; TST, total sleep time; SE, sleep efficiency; PSQI, Pittsburgh Sleep Quality Index; FFS, Flinders Fatigue Scale; ESS, Epworth Sleepiness Scale; GAD-7, Generalized Anxiety Disorder-7; EPDS, Edinburgh Postnatal Depression Scale. In the complete cases analysis, 65 participants in the intervention group and 65 participants in the control group were included. ^a^ Mean (SE) presented is least squares mean (standard error) from mixed-effects linear regression model. ^b^ n (%) presented is the number (proportion) of remission from insomnia symptoms and achieving reliable change in ISI score. ^c^ Estimated within-group change and *P* value from mixed-effects linear regression model. ^d^ Estimated between-group differences in changes in ISI scores over time (group × time interactions) from mixed-effects linear regression model. ^e^ Estimated between-group differences in the likelihood of remission or achieving reliable change from logistic regression model (ISI score at baseline was included as a covariate). ^f^ *P* value after controlling for multiple testing due to multiple secondary outcomes using the Benjamini-Hochberg (BH) false discovery rate correction.
