# Supplementary material for: Effectiveness and Mechanisms of a Digital Mindfulness–Based Intervention for Subthreshold to Clinical Insomnia Symptoms in Pregnant Women: Randomized Controlled Trial
Source: J Med Internet Res. 2025 May 5;27:e68084. doi: 10.2196/68084 (PMC12089866; doi:10.2196/68084)
Supplement: Multimedia Appendix 9 [file jmir_v27i1e68084_app9.doc]

Mixed-effects analysis of change in primary and secondary outcomes from baseline to follow-up (as-treated analysis)

|  | Mean (SE) ^a^/ n (%) ^b^ | | | |  | Change from time 1 to time 2 | | | | |  | Change from time 1 to time 3 | | | | |  | Change from time 1 to time 4 | | | | |
| --- | --- | --- | --- | --- | --- | --- | --- | --- | --- | --- | --- | --- | --- | --- | --- | --- | --- | --- | --- | --- | --- | --- |
|  |  |  |  |  |  | within-group ^c^ | | between-group difference | | |  | within-group ^c^ | | between-group difference | | |  | within-group ^c^ | | between-group difference | | |
| Measure | Time 1 (baseline) | Time 2 (post-intervention) | Time 3 (two months after post-intervention) | Time 4 (42 days postpartum) |  | change in score | *P* value | *β* (95% *CI*) ^d^/  *OR* (95% *CI*) ^e^ | *P* value | Adjusted *P* value ^f^ |  | change in score | *P* value | *β* (95% *CI*) ^d^/  *OR* (95% *CI*) ^e^ | *P* value | Adjusted *P* value ^f^ |  | change in score | *P* value | *β* (95% *CI*) ^d^/  *OR* (95% *CI*) ^e^ | *P* value | Adjusted *P* value ^f^ |
| **Primary outcome: ISI scores ^a^** | | | | | | | | | | | | | | | | | | | | | | |
| dMBI-PI+TAU | 11.08 (0.60) | 5.32 (0.60) | 6.70 (0.60) | 7.44 (0.61) |  | -5.76 | <0.001 | -2.39 (-4.05 to -0.73) | 0.005 | NA |  | -4.38 | <0.001 | -1.95 (-3.61 to -0.28) | 0.022 | NA |  | -3.64 | <0.001 | -1.95 (-3.44 to -0.05) | 0.044 | NA |
| TAU | 10.04 (0.41) | 6.67 (0.42) | 7.61 (0.42) | 8.15 (0.45) |  | -3.37 | <0.001 |  |  |  |  | -2.43 | <0.001 |  |  |  |  | -1.89 | 0.001 |  |  |  |
| **Secondary outcome: rate of remission from insomnia symptoms ^b^** | | | | | | | | | | | | | | | | | | | | | | |
| dMBI-PI+TAU | NA | 29 (78.40) | 25 (67.60) | 20 (54.10) |  | NA | NA | 2.92 (1.11 to 7.73) | 0.031 | 0.084 |  | NA | NA | 2.60 (1.11 to 6.12) | 0.028 | 0.098 |  | NA | NA | 1.52 (0.65 to 3.56) | 0.331 | 0.463 |
| TAU | NA | 45 (56.30) | 34 (42.50) | 31 (38.80) |  | NA | NA |  |  |  |  | NA | NA |  |  |  |  | NA | NA |  |  |  |
| **Secondary outcome: rate of achieving reliable change in ISI scores ^b^** | | | | | | | | | | | | | | | | | | | | | | |
| dMBI-PI+TAU | NA | 31 (83.80) | 26 (70.30) | 21 (56.80) |  | NA | NA | 3.53 (1.26 to 9.94) | 0.017 | 0.084 |  | NA | NA | 3.07 (1.26 to 7.47) | 0.014 | 0.098 |  | NA | NA | 1.56 (0.67 to 3.63) | 0.305 | 0.463 |
| TAU | NA | 41 (51.20) | 29 (36.30) | 29 (36.30) |  | NA | NA |  |  |  |  | NA | NA |  |  |  |  | NA | NA |  |  |  |
| **Secondary outcome: SOL (mins) ^a^** | | | | | | | | | | | | | | | | | | | | | | |
| dMBI-PI+TAU | 28.00 (3.85) | 25.1 (3.85) | NA | NA |  | -2.84 | 0.424 | -1.53 (-9.97 to 6.90) | 0.722 | 0.722 |  | NA | NA | NA | NA |  |  | NA | NA | NA | NA | NA |
| TAU | 35.1 (2.62) | 33.8 (2.73) | NA | NA |  | -1.30 | 0.607 |  |  |  |  | NA | NA |  |  |  |  | NA | NA |  |  |  |
| **Secondary outcome: WASO (mins) ^a^** | | | | | | | | | | | | | | | | | | | | | | |
| dMBI-PI+TAU | 18.85 (2.16) | 9.66 (2.16) | NA | NA |  | -9.19 | <0.001 | -5.77 (-11.32 to -0.22) | 0.044 | 0.084 |  | NA | NA | NA | NA |  |  | NA | NA | NA | NA | NA |
| TAU | 15.52 (1.47) | 12.10 (1.55) | NA | NA |  | -3.43 | 0.041 |  |  |  |  | NA | NA |  |  |  |  | NA | NA |  |  |  |
| **Secondary outcome: TST (hours) ^a^** | | | | | | | | | | | | | | | | | | | | | | |
| dMBI-PI+TAU | 7.85 (0.14) | 8.05 (0.14) | NA | NA |  | 0.20 | 0.097 | 0.06 (-0.22 to 0.34) | 0.675 | 0.722 |  | NA | NA | NA | NA |  |  | NA | NA | NA | NA | NA |
| TAU | 7.98 (0.10) | 8.12 (0.10) | NA | NA |  | 0.14 | 0.106 |  |  |  |  | NA | NA |  |  |  |  | NA | NA |  |  |  |
| **Secondary outcome: SE (%) ^a^** | | | | | | | | | | | | | | | | | | | | | | |
| dMBI-PI+TAU | 0.85 (0.01) | 0.89 (0.01) | NA | NA |  | 0.04 | <0.001 | 0.03 (0.00 to 0.05) | 0.023 | 0.084 |  | NA | NA | NA | NA |  |  | NA | NA | NA | NA | NA |
| TAU | 0.86 (0.01) | 0.87 (0.01) | NA | NA |  | 0.01 | 0.065 |  |  |  |  | NA | NA |  |  |  |  | NA | NA |  |  |  |
| **Secondary outcome: PSQI ^a^** | | | | | | | | | | | | | | | | | | | | | | |
| dMBI-PI+TAU | 8.68 (0.47) | 5.48 (0.48) | 6.43 (0.47) | 8.81 (0.48) |  | -3.20 | <0.001 | -1.50 (-2.82 to -0.18) | 0.027 | 0.084 |  | -2.24 | <0.001 | -1.27 (-2.59 to 0.05) | 0.059 | 0.138 |  | 0.14 | 0.995 | -1.16 (-2.51 to 0.19) | 0.094 | 0.329 |
| TAU | 8.09 (0.32) | 6.39 (0.33) | 7.12 (0.34) | 9.38 (0.36) |  | -1.70 | <0.001 |  |  |  |  | -0.97 | 0.064 |  |  |  |  | 1.30 | 0.009 |  |  |  |
| **Secondary outcome: FFS ^a^** | | | | | | | | | | | | | | | | | | | | | | |
| dMBI-PI+TAU | 10.49 (0.68) | 7.32 (0.68) | 8.41 (0.68) | 10.15 (0.68) |  | -3.16 | <0.001 | -1.30 (-3.01 to 0.40) | 0.135 | 0.212 |  | -2.08 | 0.021 | -1.07 (-2.77 to 0.64) | 0.222 | 0.281 |  | -0.34 | 0.966 | -0.93 (-2.67 to 0.81) | 0.294 | 0.463 |
| TAU | 10.57 (0.46) | 8.72 (0.48) | 9.56 (0.48) | 11.17 (0.50) |  | -1.86 | 0.002 |  |  |  |  | -1.02 | 0.188 |  |  |  |  | 0.59 | 0.673 |  |  |  |
| **Secondary outcome: ESS ^a^** | | | | | | | | | | | | | | | | | | | | | | |
| dMBI-PI+TAU | 9.11 (0.86) | 7.82 (0.87) | 7.86 (0.86) | 10.44 (0.87) |  | -1.29 | 0.341 | -0.94 (-2.62 to 0.75) | 0.276 | 0.380 |  | -1.24 | 0.367 | -1.01 (-2.69 to 0.67) | 0.241 | 0.281 |  | 1.34 | 0.318 | -0.24 (-1.94 to 1.45) | 0.777 | 0.907 |
| TAU | 9.69 (0.58) | 9.04 (0.60) | 9.69 (0.60) | 10.67 (0.63) |  | -0.65 | 0.621 |  |  |  |  | 0.00 | 1.000 |  |  |  |  | 0.98 | 0.307 |  |  |  |
| **Secondary outcome: GAD-7 ^a^** | | | | | | | | | | | | | | | | | | | | | | |
| dMBI-PI+TAU | 6.41 (0.51) | 4.00 (0.51) | 4.76 (0.51) | 4.76 (0.51) |  | -2.41 | <0.001 | -1.23 (-2.43 to -0.03) | 0.046 | 0.084 |  | -1.64 | 0.008 | -0.93 (-2.14 to 0.28) | 0.131 | 0.229 |  | -1.64 | 0.008 | -1.27 (-2.49 to -0.04) | 0.043 | 0.301 |
| TAU | 6.29 (0.35) | 5.11 (0.36) | 5.58 (0.36) | 5.91 (0.37) |  | -1.18 | 0.005 |  |  |  |  | -0.71 | 0.192 |  |  |  |  | -0.37 | 0.742 |  |  |  |
| **Secondary outcome: EPDS ^a^** | | | | | | | | | | | | | | | | | | | | | | |
| dMBI-PI+TAU | 8.51 (0.64) | 7.03 (0.84) | 7.00 (0.84) | 7.49 (0.84) |  | -1.49 | 0.226 | -0.36 (-2.21 to 1.49) | 0.703 | 0.722 |  | -1.51 | 0.212 | -0.33 (-2.18 to 1.52) | 0.724 | 0.724 |  | -1.03 | 0.559 | -0.08 (-1.96 to 1.81) | 0.937 | 0.937 |
| TAU | 9.70 (0.57) | 8.57 (0.59) | 8.52 (0.59) | 8.75 (0.61) |  | -1.13 | 0.171 |  |  |  |  | -1.18 | 0.139 |  |  |  |  | -0.95 | 0.342 |  |  |  |

Abbreviations: dMBI-PI, digital mindfulness-based intervention for prenatal insomnia symptoms; TAU, treatment as usual; NA, not applicable; ISI, Insomnia Severity Index; SOL, sleep onset latency; WASO, wake after sleep onset; TST, total sleep time; SE, sleep efficiency; PSQI, Pittsburgh Sleep Quality Index; FFS, Flinders Fatigue Scale; ESS, Epworth Sleepiness Scale; GAD-7, Generalized Anxiety Disorder-7; EPDS, Edinburgh Postnatal Depression Scale. In the as-treated analysis, 37 participants in the intervention group and 80 participants in the control group were included. ^a^ Mean (SE) presented is least squares mean (standard error) from mixed-effects linear regression model. ^b^ n (%) presented is the number (proportion) of remission from insomnia symptoms and achieving reliable change in ISI score. ^c^ Estimated within-group change and *P* value from mixed-effects linear regression model. ^d^ Estimated between-group differences in changes in ISI scores over time (group × time interactions) from mixed-effects linear regression model. ^e^ Estimated between-group differences in the likelihood of remission or achieving reliable change from logistic regression model (ISI score at baseline was included as a covariate). ^f^ *P* value after controlling for multiple testing due to multiple secondary outcomes using the Benjamini-Hochberg (BH) false discovery rate correction.
