# Supplementary material for: Effectiveness and Mechanisms of a Digital Mindfulness–Based Intervention for Subthreshold to Clinical Insomnia Symptoms in Pregnant Women: Randomized Controlled Trial
Source: J Med Internet Res. 2025 May 5;27:e68084. doi: 10.2196/68084 (PMC12089866; doi:10.2196/68084)
Supplement: Multimedia Appendix 10 [file jmir_v27i1e68084_app10.doc]

Differences in the change status of insomnia symptoms during the study period between adherent and non-adherent participants

|  | Non-adherent participants  n(%) | Adherent participants  n(%) | *χ*^2^ value | *P* value |
| --- | --- | --- | --- | --- |
| Remission/deterioration/persistence of insomnia symptoms from Time 1 to Time 2^a^ |  |  | 4.117 | 0.199 |
| Partial remission from clinical insomnia symptoms | 1 (2.9) | 4 (10.8) |  |  |
| Complete remission | 25 (71.4) | 29 (78.4) |  |  |
| Persistent sub-threshold or clinical insomnia symptoms | 8 (22.9) | 4 (10.8) |  |  |
| Progression from sub-threshold to clinical insomnia symptoms | 1 (2.9) | 0 (0.00) |  |  |
| Remission/deterioration/persistence of insomnia symptoms from Time 1 to Time 3^a^ |  |  | 5.441 | 0.128 |
| Partial remission from clinical insomnia symptoms | 2 (5.6) | 2 (5.4) |  |  |
| Complete remission | 25 (69.4) | 25 (67.6) |  |  |
| Persistent sub-threshold or clinical insomnia symptoms | 5 (13.9) | 10 (27.0) |  |  |
| Progression from sub-threshold to clinical insomnia symptoms | 4 (11.1) | 0 (0.0) |  |  |
| Remission/deterioration/persistence of insomnia symptoms from Time 1 to Time 4^a^ |  |  | 3.064 | 0.385 |
| Partial remission from clinical insomnia symptoms | 4 (12.9) | 5 (13.9) |  |  |
| Complete remission | 12 (38.7) | 20 (55.6) |  |  |
| Persistent sub-threshold or clinical insomnia symptoms | 10 (32.3) | 9 (25.0) |  |  |
| Progression from sub-threshold to clinical insomnia symptoms | 5 (16.1) | 2 (5.6) |  |  |
| Reliable change of insomnia symptoms from Time 1 to Time 2^b^ |  |  | 4.519 | 0.088 |
| Significant deterioration | 2 (5.7) | 0 (0.0) |  |  |
| No significant change | 11 (31.4) | 6 (16.2) |  |  |
| Significant improvement | 22 (62.9) | 31 (83.8) |  |  |
| Reliable change of insomnia symptoms from Time 1 to Time 3^b^ |  |  | 0.361 | 0.930 |
| Significant deterioration | 3 (8.3) | 2 (5.4) |  |  |
| No significant change | 9 (25.0) | 9 (24.3) |  |  |
| Significant improvement | 24 (66.7) | 26 (70.3) |  |  |
| Reliable change of insomnia symptoms from Time 1 to Time 4^b^ |  |  | 0.431 | 0.884 |
| Significant deterioration | 5 (16.1) | 4 (11.1) |  |  |
| No significant change | 9 (29.0) | 11 (30.6) |  |  |
| Significant improvement | 17 (54.8) | 21 (58.3) |  |  |

Note: Time 1, baseline; Time 2, post-intervention; Time 3, two months after post-intervention; Time 4, 42 days postpartum. ^a^ According to the severtiy of insomnia symptoms at baseline and follow up, participants were classified into four groups: partial remission from clinical insomnia symptoms (who reported clinical insomnia symptoms (ISI scores ≥ 11) at baseline and sub-threshold insomnia symptoms (8 ≤ ISI socres <11) at follow-up), complete remission (who reported sub-threshold to clinical insomnia symptoms at baseline, and remission from insomnia symptoms (ISI socres <8) at follow up), persistent sub-threshold or clinical insomnia symptoms (who reported sub-threshold or clinical insomnia symptoms at both baseline and follow-up), and progression from sub-threshold to clinical insomnia symptoms (who reported sub-threshold insomnia symptoms at baseline, and clinical insomnia symptoms at follow-up). ^b^ According to the change in ISI scores from baseline to follow-up, participants were classified into four groups: significant deterioration (who reported a higher ISI score at follow-up than baseline, and the difference exceeded the reliable change criterion of 2.52 points), no significant change (whose changes in ISI scores from baseline to follow-up did not exceed the reliable change criterion), and significant improvement (who reported a lower ISI score at follow-up than baseline, and the difference exceeded the reliable criterion).
