# Supplementary material for: Effectiveness and Mechanisms of a Digital Mindfulness–Based Intervention for Subthreshold to Clinical Insomnia Symptoms in Pregnant Women: Randomized Controlled Trial
Source: J Med Internet Res. 2025 May 5;27:e68084. doi: 10.2196/68084 (PMC12089866; doi:10.2196/68084)
Supplement: Multimedia Appendix 11 [file jmir_v27i1e68084_app11.doc]

Associations between baseline characteristics and intervention adherence

| Baseline characteristics | | *B* | *SE* | *Wald* | *P* | *OR* (95% *CI*) |
| --- | --- | --- | --- | --- | --- | --- |
| Age (years) | | 0.09 | 0.09 | 0.99 | 0.319 | 1.10 (0.91 to 1.32) |
| Education | Junior college or less | Reference | | | | |
|  | **Bachelor’s degree or above** | **1.99** | **0.80** | **6.20** | **0.013** | **7.28 (1.53 to 34.74)** |
| Marital Status | Unmarried (cohabitation) | Reference | | | | |
|  | Married | 0.51 | 1.64 | 0.10 | 0.758 | 1.66 (0.07 to 41.37) |
| BMI at the time of participation (kg/m2) | | -0.07 | 0.07 | 1.05 | 0.305 | 0.93 (0.81 to 1.07) |
| Per capita monthly household Income (RMB) | <3500 | Reference | | | | |
|  | ≥3500 | -0.37 | 1.02 | 0.13 | 0.715 | 0.69 (0.09 to 5.07) |
| Living area | Urban | Reference | | | | |
|  | Rural | 1.03 | 1.13 | 0.82 | 0.364 | 2.79 (0.30 to 25.58) |
| Work status | Unemployed | Reference | | | | |
|  | Employed | -0.52 | 1.09 | 0.22 | 0.637 | 0.60 (0.07 to 5.10) |
| Pre-pregnancy sleep quality | Good | Reference | | | | |
|  | Poor | -0.80 | 0.63 | 1.60 | 0.206 | 0.45 (0.13 to 1.55) |
| Gestational age (weeks) | | 0.08 | 0.11 | 0.50 | 0.479 | 1.08 (0.87 to 1.33) |
| Gravidity | 1 | Reference | | | | |
|  | 2 | -1.63 | 1.05 | 2.42 | 0.120 | 0.20 (0.03 to 1.53) |
|  | ≥3 | -2.67 | 1.52 | 3.10 | 0.078 | 0.07 (0.00 to 1.35) |
| Parity | Primipara | Reference | | | | |
|  | Multipara | 1.73 | 1.14 | 2.28 | 0.131 | 5.61 (0.60 to 52.60) |
| Adverse obstetric history | No | Reference | | | | |
|  | Yes | 0.53 | 1.03 | 0.27 | 0.607 | 1.70 (0.23 to 12.79) |
| Pregnancy complications | Yes | Reference | | | | |
|  | No | 0.45 | 0.66 | 0.46 | 0.497 | 1.56 (0.43 to 5.68) |
| Planned pregnancy | Yes | Reference | | | | |
|  | No | -1.13 | 0.79 | 2.06 | 0.151 | 0.32 (0.07 to 1.51) |
| Insomnia symptom severity | | 0.11 | 0.11 | 1.04 | 0.308 | 1.12 (0.90 to 1.37) |

Note: BMI, body mass index. Due to the fact that only a small number of pregnant women belonged to minority groups and used assisted reproductive technologies, the logistic regression analysis did not include race and mode of pregnancy. Additionally, considering the high correlation between pre-pregnancy BMI and BMI at the time of participation, only the BMI at the time of participation was retained in the regression analysis.
