# Supplementary material for: Effectiveness and Mechanisms of a Digital Mindfulness–Based Intervention for Subthreshold to Clinical Insomnia Symptoms in Pregnant Women: Randomized Controlled Trial
Source: J Med Internet Res. 2025 May 5;27:e68084. doi: 10.2196/68084 (PMC12089866; doi:10.2196/68084)
Supplement: Multimedia Appendix 12 [file jmir_v27i1e68084_app12.doc]

Mixed-effects analysis of change in hypothesized mediators from baseline to the end of the intervention using imputed datasets

|  | Mean (SE) ^a^ | |  | within-group ^b^ | | between-group difference ^c^ | | |
| --- | --- | --- | --- | --- | --- | --- | --- | --- |
| Measure | Time 1 (baseline) | Time 2 (immediately after the intervention) |  | change in score | *P* value | *β* (95% *CI*) | *P* value | Adjusted *P* value ^d^ |
| **DISRS** | | | | | | | | |
| dMBI-PI+TAU | 35.60 (1.05) | 29.30 (1.08) |  | -6.27 | <0.001 | -1.84 (-4.69 to 1.01) | 0.205 | 0.205 |
| TAU | 37.80 (1.05) | 33.40 (1.07) |  | -4.43 | <0.001 |  |  |  |
| **APSQ** | | | | | | | | |
| dMBI-PI+TAU | 50.40 (2.51) | 32.50 (2.62) |  | -17.88 | <0.001 | -10.66 (-18.57 to -2.74) | 0.009 | 0.045 |
| TAU | 50.40 (2.51) | 43.20 (2.60) |  | -7.22 | 0.012 |  |  |  |
| **PSAS** | | | | | | | | |
| dMBI-PI+TAU | 30.20 (0.86) | 25.20 (0.90) |  | -4.95 | <0.001 | -2.60 (-4.86 to -0.35) | 0.024 | 0.040 |
| TAU | 30.80 (0.86) | 28.40 (0.89) |  | -2.35 | 0.005 |  |  |  |
| **SAMI-B** | | | | | | | | |
| dMBI-PI+TAU | 20.80 (0.62) | 16.40 (0.64) |  | -4.46 | <0.001 | -2.27 (-4.21 to -0.32) | 0.022 | 0.040 |
| TAU | 20.70 (0.62) | 18.50 (0.63) |  | -2.20 | 0.002 |  |  |  |
| **SRBQ** | | | | | | | | |
| dMBI-PI+TAU | 36.50 (2.06) | 29.10 (2.22) |  | -7.35 | 0.002 | -6.45 (-12.67 to -0.22) | 0.042 | 0.053 |
| TAU | 39.90 (2.06) | 39.00 (2.12) |  | -0.91 | 0.680 |  |  |  |

Abbreviations: dMBI-PI, digital mindfulness-based intervention for prenatal insomnia symptoms; TAU, treatment as usual; DISRS, Daytime Insomnia Symptom Response Scale; APSQ, Anxiety and Preoccupation about Sleep Questionnaire; PSAS, Pre-Sleep Arousal Scale; SAMI-B, Brief Version of the Sleep-Associated Monitoring Index; SRBQ, Sleep-Related Behaviors Questionnaire. ^a^ Mean (SE) presented is least squares mean (standard error) from mixed-effects linear regression model. ^b^ Estimated within-group change and *P* value from mixed-effects linear regression model. ^c^ Estimated between-group differences in changes in ISI scores over time (group × time interactions) from mixed-effects linear regression model. ^d^ *P* value after controlling for multiple testing due to multiple hypothesized mediators using the Benjamini-Hochberg (BH) false discovery rate correction.
