# Supplementary material for: Effectiveness and Mechanisms of a Digital Mindfulness–Based Intervention for Subthreshold to Clinical Insomnia Symptoms in Pregnant Women: Randomized Controlled Trial
Source: J Med Internet Res. 2025 May 5;27:e68084. doi: 10.2196/68084 (PMC12089866; doi:10.2196/68084)
Supplement: Multimedia Appendix 13 [file jmir_v27i1e68084_app13.doc]

Mixed-effects analysis of change in hypothesized mediators from baseline to the end of the intervention (complete cases analysis)

|  | Mean (SE) ^a^ | |  | within-group ^b^ | | between-group difference ^c^ | | |
| --- | --- | --- | --- | --- | --- | --- | --- | --- |
| Measure | Time 1 (baseline) | Time 2 (immediately after the intervention) |  | change in score | *P* value | *β* (95% *CI*) | *P* value | Adjusted *P* value ^d^ |
| **DISRS** | | | | | | | | |
| dMBI-PI+TAU | 36.40 (1.18) | 29.10 (1.18) |  | -7.37 | <0.001 | -3.06 (-6.16 to 0.03) | 0.055 | 0.055 |
| TAU | 38.30 (1.18) | 34.00 (1.18) |  | -4.31 | <0.001 |  |  |  |
| **APSQ** | | | | | | | | |
| dMBI-PI+TAU | 51.00 (2.82) | 31.60 (2.82) |  | -19.37 | <0.001 | -12.42 (-20.70 to -4.13) | 0.004 | 0.020 |
| TAU | 50.70 (2.82) | 43.80 (2.82) |  | -6.95 | 0.023 |  |  |  |
| **PSAS** | | | | | | | | |
| dMBI-PI+TAU | 30.00 (0.96) | 24.90 (0.96) |  | -5.14 | <0.001 | -3.00 (-5.44 to -0.56) | 0.018 | 0.045 |
| TAU | 30.80 (0.96) | 28.60 (0.96) |  | -2.14 | 0.018 |  |  |  |
| **SAMI-B** | | | | | | | | |
| dMBI-PI+TAU | 21.00 (0.70) | 16.30 (0.70) |  | -4.69 | <0.001 | -2.40 (-4.57 to -0.23) | 0.032 | 0.048 |
| TAU | 20.80 (0.70) | 18.50 (0.70) |  | -2.29 | 0.004 |  |  |  |
| **SRBQ** | | | | | | | | |
| dMBI-PI+TAU | 36.70 (2.28) | 28.00 (2.28) |  | -8.66 | <0.001 | -6.95 (-13.47 to -0.44) | 0.038 | 0.048 |
| TAU | 40.10 (2.28) | 38.40 (2.28) |  | -1.71 | 0.472 |  |  |  |

Abbreviations: dMBI-PI, digital mindfulness-based intervention for prenatal insomnia symptoms; TAU, treatment as usual; DISRS, Daytime Insomnia Symptom Response Scale; APSQ, Anxiety and Preoccupation about Sleep Questionnaire; PSAS, Pre-Sleep Arousal Scale; SAMI-B, Brief Version of the Sleep-Associated Monitoring Index; SRBQ, Sleep-Related Behaviors Questionnaire. In the complete cases analysis, 65 participants in the intervention group and 65 participants in the control group were included.^a^ Mean (SE) presented is least squares mean (standard error) from mixed-effects linear regression model. ^b^ Estimated within-group change and *P* value from mixed-effects linear regression model. ^c^ Estimated between-group differences in changes in ISI scores over time (group × time interactions) from mixed-effects linear regression model. ^d^ *P* value after controlling for multiple testing due to multiple hypothesized mediators using the Benjamini-Hochberg (BH) false discovery rate correction.
