# Supplementary material for: Effectiveness and Mechanisms of a Digital Mindfulness–Based Intervention for Subthreshold to Clinical Insomnia Symptoms in Pregnant Women: Randomized Controlled Trial
Source: J Med Internet Res. 2025 May 5;27:e68084. doi: 10.2196/68084 (PMC12089866; doi:10.2196/68084)
Supplement: Multimedia Appendix 14 [file jmir_v27i1e68084_app14.doc]

Mixed-effects analysis of change in hypothesized mediators from baseline to the end of the intervention (as-treated analysis)

|  | Mean (SE) ^a^ | |  | within-group ^b^ | | between-group difference ^c^ | | |
| --- | --- | --- | --- | --- | --- | --- | --- | --- |
| Measure | Time 1 (baseline) | Time 2 (immediately after intervention) |  | change in score | *P* value | *β* (95% *CI*) | *P* value | Adjusted *P* value ^d^ |
| **DISRS** | | | | | | | | |
| dMBI-PI+TAU | 35.00 (1.49) | 29.10 (1.49) |  | -5.92 | <0.001 | -1.67 (-4.95 to 1.62) | 0.322 | 0.322 |
| TAU | 37.80 (1.01) | 33.60 (1.04) |  | -4.25 | <0.001 |  |  |  |
| **APSQ** | | | | | | | | |
| dMBI-PI+TAU | 55.40 (3.71) | 33.40 (3.71) |  | -22.00 | <0.001 | -15.51 (-24.80 to -6.22) | 0.001 | 0.005 |
| TAU | 50.40 (2.52) | 43.90 (2.60) |  | -6.49 | 0.020 |  |  |  |
| **PSAS** | | | | | | | | |
| dMBI-PI+TAU | 29.80 (1.24) | 24.80 (1.24) |  | -4.95 | <0.001 | -2.59 (-5.48 to 0.30) | 0.081 | 0.160 |
| TAU | 30.80 (0.84) | 28.40 (0.87) |  | -2.35 | 0.007 |  |  |  |
| **SAMI-B** | | | | | | | | |
| dMBI-PI+TAU | 20.80 (0.86) | 16.80 (0.86) |  | -3.92 | <0.001 | -1.73 (-4.02 to 0.56) | 0.141 | 0.176 |
| TAU | 20.70 (0.59) | 18.50 (0.61) |  | -2.19 | 0.002 |  |  |  |
| **SRBQ** | | | | | | | | |
| dMBI-PI+TAU | 37.10 (2.96) | 30.50 (2.96) |  | -6.65 | 0.029 | -6.11 (-13.24 to 1.03) | 0.096 | 0.160 |
| TAU | 39.90 (2.01) | 39.40 (2.07) |  | -0.54 | 0.797 |  |  |  |

Abbreviations: dMBI-PI, digital mindfulness-based intervention for prenatal insomnia symptoms; TAU, treatment as usual; DISRS, Daytime Insomnia Symptom Response Scale; APSQ, Anxiety and Preoccupation about Sleep Questionnaire; PSAS, Pre-Sleep Arousal Scale; SAMI-B, Brief Version of the Sleep-Associated Monitoring Index; SRBQ, Sleep-Related Behaviors Questionnaire. In the as-treated analysis, 37 participants in the intervention group and 80 participants in the control group were included. ^a^ Mean (SE) presented is least squares mean (standard error) from mixed-effects linear regression model. ^b^ Estimated within-group change and *P* value from mixed-effects linear regression model. ^c^ Estimated between-group differences in changes in ISI scores over time (group × time interactions) from mixed-effects linear regression model. ^d^ *P* value after controlling for multiple testing due to multiple hypothesized mediators using the Benjamini-Hochberg (BH) false discovery rate correction.
