# Supplementary material for: Effectiveness and Mechanisms of a Digital Mindfulness–Based Intervention for Subthreshold to Clinical Insomnia Symptoms in Pregnant Women: Randomized Controlled Trial
Source: J Med Internet Res. 2025 May 5;27:e68084. doi: 10.2196/68084 (PMC12089866; doi:10.2196/68084)
Supplement: Multimedia Appendix 15 [file jmir_v27i1e68084_app15.doc]

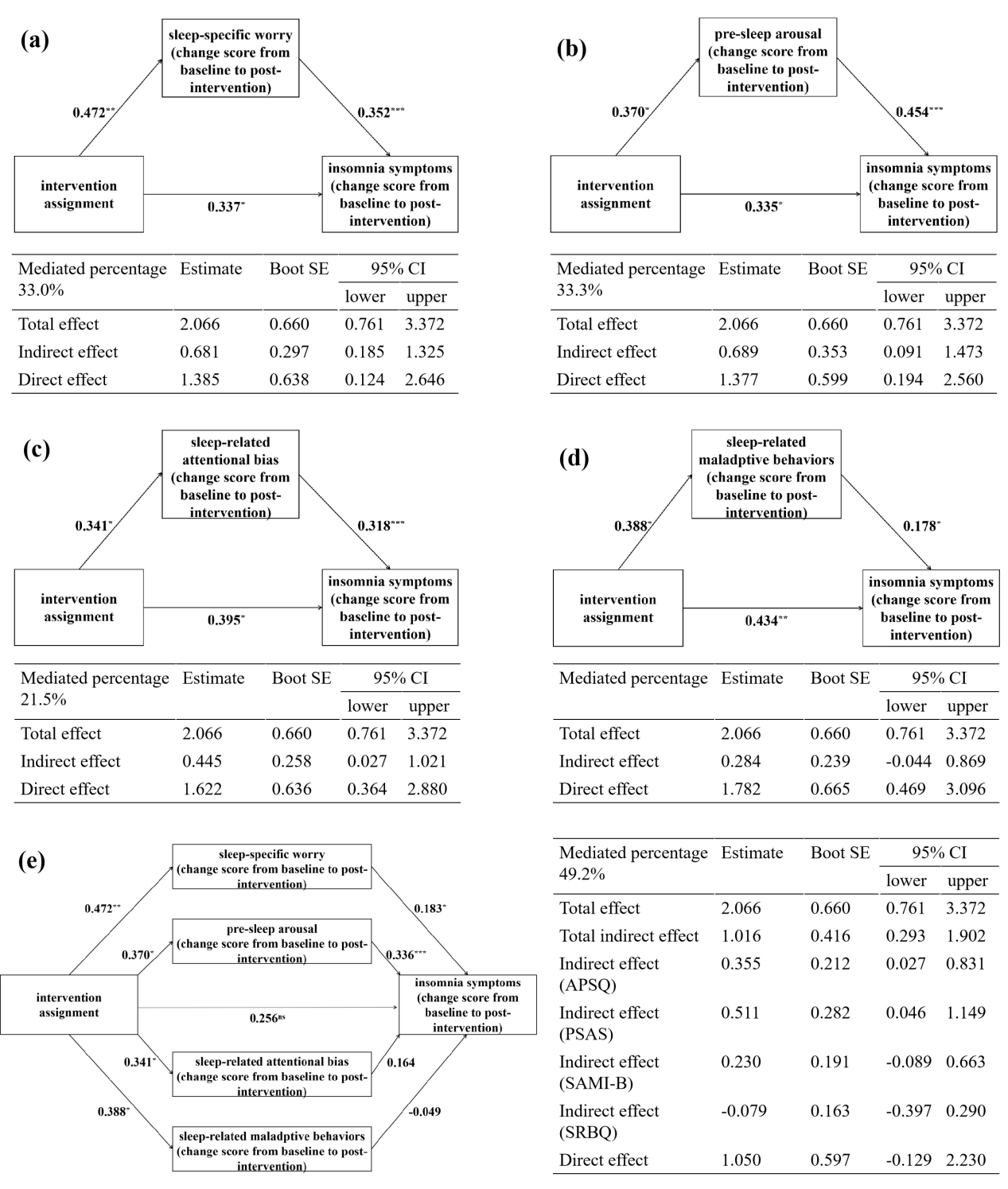


The mediation effects of adverse sleep-related cognitive and behavioral factors (raw difference scores from baseline to the end of the intervention) on the relationship between treatment assignment and raw difference scores in Insomnia Severity Index from baseline to the end of the intervention. APSQ indicates the Anxiety and Preoccupation about Sleep Questionnaire that used to assess sleep-specific worry. PSAS indicates the Pre-Sleep Arousal Scale that used to assess pre-sleep arousal. SAMI-B indicates the Brief Version of the Sleep-Associated Monitoring Index that used to assess sleep-related attentional bias. SRBQ indicates the Sleep-Related Behaviors Questionnaire that used to assess sleep-related maladptive behaviors. ^*^*p* <0.05; ^**^*p* <0.01; ^***^*p* <0.001; ^ns^ statistically not significant.
