# Supplementary material for: Effectiveness and Mechanisms of a Digital Mindfulness–Based Intervention for Subthreshold to Clinical Insomnia Symptoms in Pregnant Women: Randomized Controlled Trial
Source: J Med Internet Res. 2025 May 5;27:e68084. doi: 10.2196/68084 (PMC12089866; doi:10.2196/68084)
Supplement: Multimedia Appendix 16 [file jmir_v27i1e68084_app16.doc]

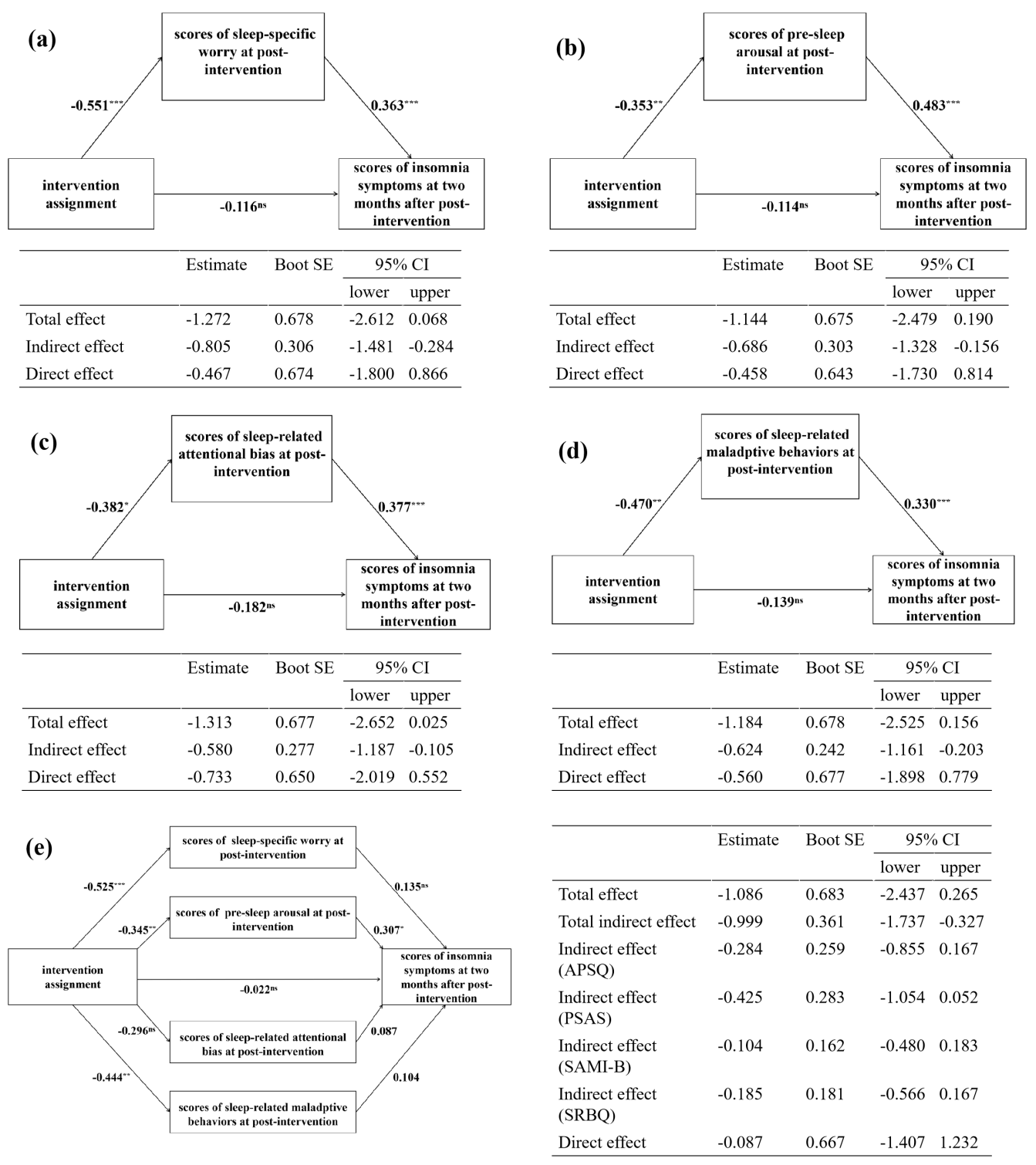


The mediation effects of adverse sleep-related cognitive and behavioral factors at the end of the intervention on the relationship between treatment assignment and insomnia symptoms at 2 months after the intervention. (a) Single mediation model controlled for baseline scores of insomnia symptoms and sleep-specific worry. (b) Single mediation model controlled for baseline scores of insomnia symptoms and pre-sleep arousal. (c) Single mediation model controlled for baseline scores of insomnia symptoms and sleep-related attentional bias. (d) Single mediation model controlled for baseline scores of insomnia symptoms and sleep-related maladptive behaviors. (e) Multiple mediation model controlled for baseline scores of insomnia symptoms and hypothesized mediators (including sleep-specific worry, pre-sleep arousal, sleep-related attentional bias and sleep-related maladptive behaviors). APSQ indicates the Anxiety and Preoccupation about Sleep Questionnaire that used to assess sleep-specific worry. PSAS indicates the Pre-Sleep Arousal Scale that used to assess pre-sleep arousal. SAMI-B indicates the Brief Version of the Sleep-Associated Monitoring Index that used to assess sleep-related attentional bias. SRBQ indicates the Sleep-Related Behaviors Questionnaire that used to assess sleep-related maladptive behaviors. ^*^*p* <0.05; ^**^*p* <0.01; ^***^*p* <0.001; ^ns^statistically not significant.
